# Surge-type and surge-modified glaciers in the Karakoram

**DOI:** 10.1038/s41598-017-15473-8

**Published:** 2017-11-13

**Authors:** R. Bhambri, K. Hewitt, P. Kawishwar, B. Pratap

**Affiliations:** 1 Centre for Glaciology, Wadia Institute of Himalayan Geology, 33 GMS Road, Dehradun, 248001 India; 2 Department of Geography and Environmental Studies, Wilfrid Laurier University, Waterloo, Canada; 3 Chhattisgarh Council of Science and Technology, Vigyan Bhavan, Vidhan Sabha Road, Daldal Seoni, Raipur (CG), 492014 India; 4 National Centre for Antarctic and Ocean Research, Vasco-da-Gama, Goa, 403 804 India

## Abstract

Glaciers in the Karakoram exhibit irregular behavior. Terminus fluctuations of individual glaciers lack consistency and, unlike other parts of the Himalaya, total ice mass remained stable or slightly increased since the 1970s. These seeming anomalies are addressed through a comprehensive mapping of surge-type glaciers and surge-related impacts, based on satellite images (Landsat and ASTER), ground observations, and archival material since the 1840s. Some 221 surge-type and surge-like glaciers are identified in six main classes. Their basins cover 7,734 ± 271 km^2^ or ~43% of the total Karakoram glacierised area. Active phases range from some months to over 15 years. Surge intervals are identified for 27 glaciers with two or more surges, including 9 not previously reported. Mini-surges and kinematic waves are documented and surface diagnostic features indicative of surging. Surge cycle timing, intervals and mass transfers are unique to each glacier and largely out-of-phase with climate. A broad class of surge-modified ice introduces indirect and post-surge effects that further complicate tracking of climate responses. Mass balance in surge-type and surge-modified glaciers differs from conventional, climate-sensitive profiles. New approaches are required to account for such differing responses of individual glaciers, and effectively project the fate of Karakoram ice during a warming climate.

## Introduction

Karakoram glaciers exhibit varied and irregular ice movements^1–3^. There is little or no synchrony of expansion or retreat for apparently similar or neighboring ice masses. Early reports suggested they are out of phase with climate fluctuations and trends observed elsewhere^3–6^. In recent years, the Karakoram has not undergone the substantial ice mass reductions or pervasive glacier retreat observed elsewhere in the Himalaya^6–12^. The region does have one of the world’s highest concentrations of surge-type glaciers^5,13–15^ (Supplementary Table S1), the focus of this paper.

*Surging* refers to episodes with a sudden, large increase in ice velocities, by an order of magnitude or more in some well-documented cases^16^. The shift into and out of fast flow can occur in a matter of days or weeks and it may persist from a few months to several years. In a few cases surging continues for more than a decade^16,17^. During the *active* or surge phase, large volumes of ice are transported from an upper *reservoir zone* into a lower, *receiving zone*. A wave of rapid thickening and thinning moves down-glacier, typically causing intense crevassing and over-riding of ice margin areas^16^. In many but not all cases, the terminus advances some kilometers in a few weeks or months^16,18^. Between active surging there is a *quiescent phase* lasting decades to centuries when the upper glacier rebuilds mass, the lower tongue thins and retreats, or becomes stagnant. In the Karakoram and some other regions up to six distinct phases have been reported^5^ based on terms used by Jiskoot (2011)^16^, they are:

1. *A build-up phase*: when the upper glacier is growing and the lower can be stagnant or seem to behave ‘normally’.
2. *A pre-surge phase*: when the glacier gradually speeds up and advances, possibly over several years.
3. *The surge or ‘active’ phase*: the period of fast flow.
4. *Post-surge deceleration and thinning*: the glacier slows gradually, crevassing and ablation zone ice levels decline. A slow advance may continue.
5. *Stagnation phase*: a substantial section of the ablation zone may detach and stagnate for decades usually under heavy supraglacial debris.
6. *Two or more active events*: rather than a single acceleration, two may occur separated by a few months or years. A short as well as much longer quiescent phase has been observed at Bualtar Glacier (ID 21).

**Figure 1:**
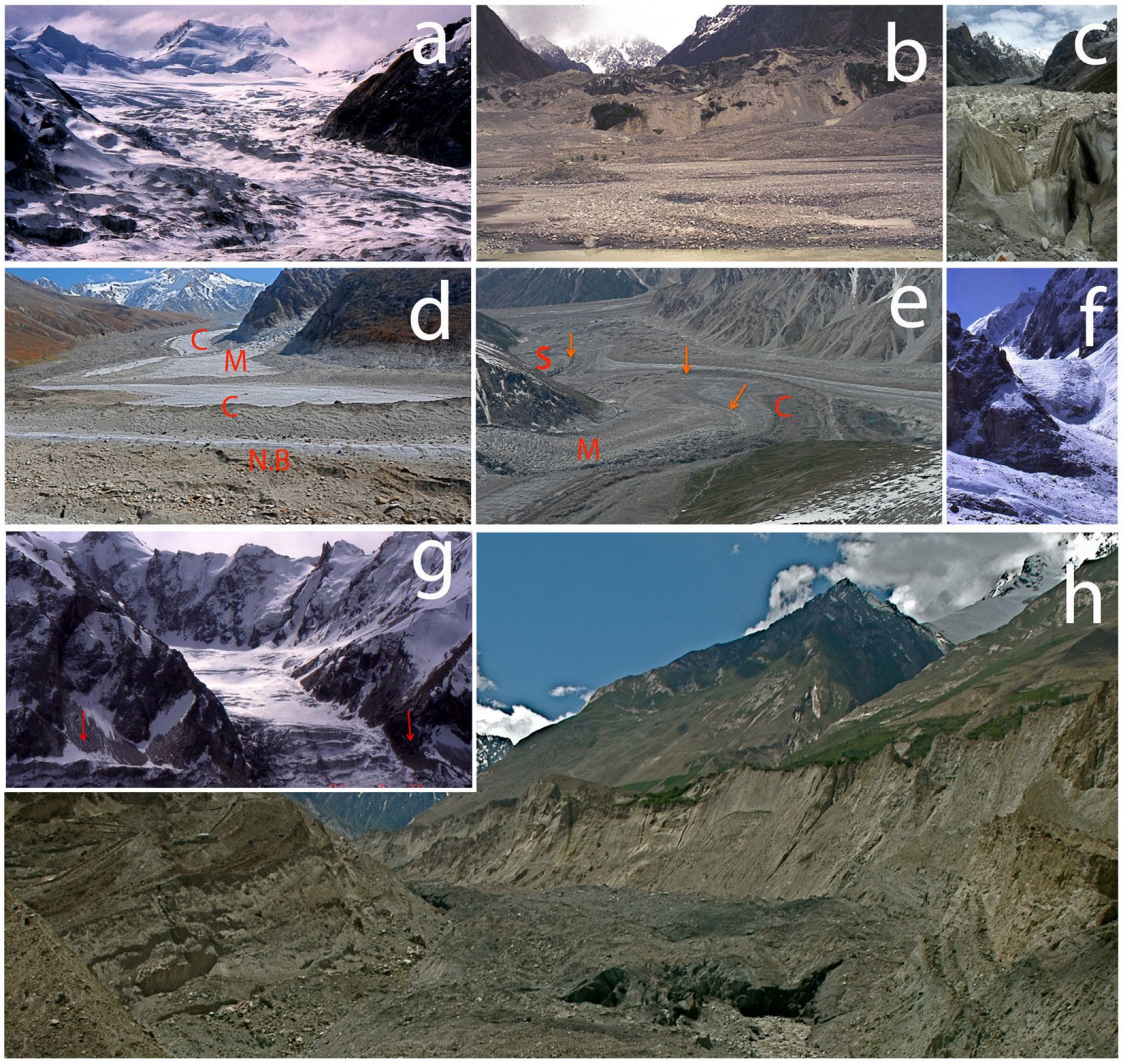
(**a**-**h**) Field observations of Karakoram surge events and features discussed in the text, including examples of what are discussed as ‘classic’ surge phenomena (**a**,**b**), tributary surges (**c**), surge-modified effects on main and tributary glaciers (**d**,**e**) and variants on surge-related behavior (**f**,**g**): (**a**) Upper Chiring Glacier showing collapse and heavy crevassing of ‘reservoir zone’, viewed one year after the 1994–95 active surge. Highest crevasses are around 5,100 m asl, at or near the firn limits and climatic snow lines (Photo June 1996), (**b**) Rapidly advancing terminus (7–10 m/day) of Karumbar Glacier during the 1993 active surge of over 3 km. Vast amounts of dead ice and proglacial sediment were reactivated at the steep surge front (photo June 1993), (**c**) Tributary surge of Shingchukpi Glacier, Panmah Basin, 2005. After three years, pre-surge episode of moderately accelerated flow, the main surge was ‘classic’ in form, with highly crevassed ice, raised and truncated marginal ice and moraine from passage of main wave. Velocities 10 m per day or more. However, after the 3.5 km advance, ice was stalled in the foreground at the main glacier, shown as severely compressed and crevassed (photo July 2005), (**d**) View over the main Nobande Sobande (N.S) Glacier showing complex distortion by ice lobes from the Chiring 1994 surge (C), in background and middle ground, and Maedan surge of 2002 (M).The main glacier is highly compressed towards its right flank, and continues to adjust as a *surge-modified* glacier (photo July 2012), (**e**) Nobande Sobonde main glacier showing modifications following surges of Chiring (1994), Maedan (2002, near ground) and active Shingchukpi (2005 left middle ground). Each maintains actuate pressure ridges^30^ (marked by orange arrows) at margins of surge ice. Photo with opposite line of sight to Fig. 1d (2005), (**f**) Maedan tributary of Panmah Glacier in 1993 that would gradually advance 3.5 km and thicken for nine years before the sudden active surge of 2002 (photo September 1992), (**g**) Unnamed tributary of Chiring Glacier showing its disturbed ice fall one year after passage of the active surge wave. Arrows show maximum surge height, and 80 m cliff of sheared-office (photo June 1996), (**h**) Gradual, apparently ‘normal’ terminus advance of Bualtar Glacier 15 years after active surges of 1987 and 2000 which did not reach the terminus. (Photos © Kenneth Hewitt).

Stages 1, 2, 4 and 5 can be treated as sub-phases of the whole ‘quiescent phase’. Surge events with the three stages 1, 3 and 4, and high velocities affecting much of the ice mass, are referred to as ‘classic’^5^. Most of the science of surging was developed in relation to them and, until recently, known Karakoram examples were restricted to such events^4,5^. However, some recent field surveys^1,5^ and remote sensing^14,19,20^ including our own, recognize a greater diversity of surge phases and behavior profiles as outlined above (Fig. 1a–c). Many surge events recently identified from satellite imagery are less dramatic than classic surges^5,19^. Accelerations are relatively modest but with doubling of velocities at least^5^. Extreme crevassing or rapid terminus advances may not occur. If a three-phase profile can still be identified, a case can be made to include these less dramatic events as surge-type. Less well-defined cases are termed *surge-like* events^5^. These differing cycles reveal a wide range of dynamic instability (Table 1). The point to emphasize is that the movements observed in differing classes and sub-classes of surge events nevertheless alter and complicate the significance of evidence used to track glacier and climate change. Also, surge-type glaciers are among major causes of ice dams and glacier lake outburst floods (GLOFs). These complicate terminus behavior and create extreme dangers for populated areas and down-country infrastructure^3,21,22^.

**Table 1:** Classification of surge-type and surge-like glaciers identified in the Karakoram. More details on sub classes and examples are presented in Supplementary Tables S2 and S3.

Main class	Sub class	Type	Description	Total number	% to total number	Total area (km^2^)	% to total area
1	1a-c	Surge-type	**‘Classic’** with full, three-phase surge cycle in main glacier. Three sub-types include ‘Alaska-type’ and ‘Svalbard-type’.	69	31.2	2349 ± 82	30.4
2	2a-c	Surge-type	**Amended ‘classic’** where one of three phases is absent, or additional one (s) occur. Three sub-types recognized.	19	8.6	861.8 ± 30	11.1
3	3 a-b	Surge-type	**Tributary surge-types**, which include four sub- types.	43	19.5	1379.8 ± 48	17.8
4	4 a-c	Surge-type	**Surge- diagnostic features** used to identify likely surge-type glaciers, including a sub-set of tributaries.	32	14.5	1679.1 ± 59	21.7
5	—	Surge-like	**Mini-surges**: local, partial surges or progressive accelerations moving down glacier but lacking ‘classic’ surge cycle.	1	0.5	114.9 ± 4	1.5
6	—	Surge-like	**Locally accelerated ice** or section, including kinematic waves. These may follow from external disturbances like heavy snowfalls and seasonal changes, or landslides onto the ice.	57	25.8	1349.4 ± 47	17.4
			Total	221		7734 ± 271	

Less than 1% of glaciers worldwide have been identified as surge-type^23^. Their global distribution is uneven but includes environments from polar to subtropical regions. They are found in continental interiors and extreme high mountains, such as the high Andes of Argentina, and in High Asia, notably the Karakoram and Pamir Ranges, or smaller concentrations in the north Caucasus and Kunlun Shan Ranges^24,25^. Others, as in Svalbard, Alaska, Greenland and southern Patagonia, involve tidewater glaciers^16^.

The concept of surging and surge mechanisms developed mainly since the 1960s, and through research largely outside the Karakoram^16,18^. Studies emphasize active surge dynamics and fast flow events. There is a consensus that surging is generated by conditions internal and intrinsic to the ice mass involved, not by external forcing^16–18^. Various models are proposed involving ice thermal instability, subglacial hydrological instability, failure in deformable subglacial sediments or some combination of these factors^26–28^. Two models linking proposed mechanisms to distinct surge cycle intervals, velocities and duration are the ‘Alaskan’ and ‘Svalbard’ types (ibid). Both appear to be present in the Karakoram^5,14,19^. An unresolved question is whether event differences, elsewhere and in our surveys (Fig. 1), reflect differing controls or a more or less continuous spectrum of the same instabilities^5,19^.

A few studies suggest that climate trends between surge events could affect their scale and duration, and that extreme weather may affect the timing of surge initiation^29^. Work in the Karakoram shows an absence of direct ties of surging to climate forcing^5,14,20^. Understanding has been limited by a lack of research and data for Karakoram glacier dynamics, especially the critical sub-glacial conditions.

The discovery of many surge-type *tributary* glaciers in the Karakoram adds further complications^30^. These glaciers tend to occur at higher elevations and in smaller, steeper ice masses compared to main glaciers (Fig. 1c,f). When surge ice reaches the main glacier it generally stalls, truncating *surge length* and further complicating long term mass transfers (Fig. 1d,e).

Reports of active surges in the Karakoram go back to the early 19^th^ century^31–33^. At first they were regarded as “accidental” events due to earthquakes or avalanches, and involving absent or delayed correlations with climate trends^33^. The first comprehensive survey^24^ based on satellite imagery greatly expanded the number of confirmed and suspected surge-type glaciers. Copland *et al*.^14^ reported 90 surge-type glaciers in the Karakoram and neighborhood areas (i.e. Aghil mountains and Chang Chenmo). More recently, Rankl *et al*.^34^ added 10 more. The diverse surface movement profiles and cycles include most types reported elsewhere^5,14^.

The active phase of a surging glacier can induce a variety of responses in adjacent, non-surging ice. These are not surge processes, but surge-related secondary disturbances or lag effects, here called *surge-modified* behavior^30^. Impacts may arise above, below or beside areas of active surging, and during or following after it. Velocities, surface morphologies and debris covers are modified to introduce a range of surface morphologies, redirections and lag times in ice not of surge-type or not actively surging at the time (Fig. 1d–f). As with other external forces, however, active surges in one ice mass are not known to, and probably cannot, trigger surges in others. They may lead to local accelerations or kinematic waves (Table 1). Tributary glacier surges identified here have not triggered surging in main glaciers, nor vice versa.

Surge-modified behavior can continue and evolve for years or decades after surging, as seen after each of the multiple, independent tributary surges at Panmah and Hispar^5,13,30^ (Fig. 1d,e). Because satellite coverage only applies to recent decades, and is constrained by the frequency and quality of overpasses, it can miss or underestimate the overall extent of surge-modified ice. A full sense of the scope of surge-modified phenomena requires long-term and repeated local observations, as has emerged at Panmah since 1990^30,35^.

In the Karakoram, glacier dynamics are further modified by large rockslides that descend onto the glaciers, with an average of almost two events per year reported in recent decades^36^. The landslide debris affects glacier movement and mass balance for several decades at least^5^. Some of the glaciers are also surge-type, including Bualtar (ID 21), Lokpar-Aling (ID 120), and Chillinji (ID 3)^36,37^(Supplementary Table S1).

A major challenge concerning Karakoram glaciers is the apparent absence of the large losses of ice reported elsewhere in the Himalaya and attributed to global climate change^6,7,38^. Some studies detect a recent increase in surge activity itself and attribute that to climate warming^5,20^. Our findings lead us to propose an alternative view, that *surge-type glaciers and surge-modified activity are critical, possibly decisive, factors in buffering and reconfiguring responses to climate change*. The implications of surge activity for mass balance are emphasized and addressed in following sections.

**Figure 2:**
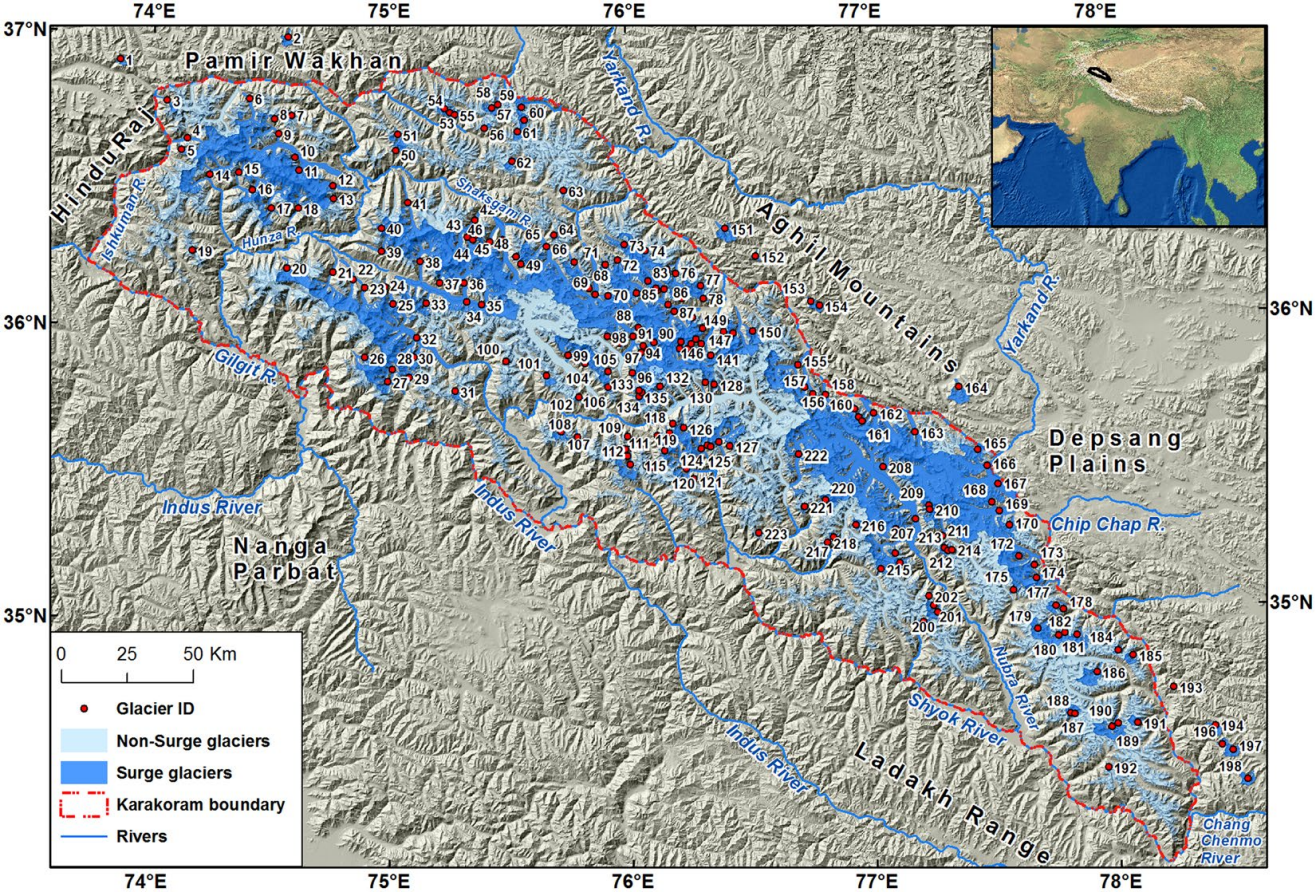
Overview of the surge-type glaciers in the Karakoram. The map of the Karakoram along with neighbouring mountain ranges (Hindu Raj, Pamir Wakhan, Aghil mountains, Depsang Plains, Ladakh and Nanga Parbat Himalaya) is generated by the open-source datasets using ArcGIS version 10.0 (http://www.esri.com/software/arcgis/arcgis-for-desktop). The hill-shaded background in this figure has been generated using the Shuttle Radar Topography Mission (SRTM) data set (90 × 90 m) provided by the CGIAR Consortium for Spatial Information (http://srtm.csi.cgiar.org/). The glacier outlines were modified (details given in ‘Methods’ section) from the Randolph Glacier Inventory (RGI 5.0) (Pfeffer *et al*.)^72^ (http://www.glims.org/RGI/00_rgi50_TechnicalNote.pdf). Details of surge-type glaciers are presented in Supplementary Table S1. The Karakoram boundary corresponds to the description given by Royal Geographical Society and Survey of India nomenclature^62^. Glacier IDs refer to glaciers shown in other figures and listed in Tables.

The aims of this study are, therefore, to extend the empirical basis for surge-type and surge-related phenomena in the study area including Karakoram and some neighboring parts of the Wakhan Pamir, Aghil and Chang Chenmo Mountains (Fig. 2) and, more specifically:

1. to provide an updated inventory of surge-type glaciers from remote sensing data, published papers and reports, ground-based observations and historical archives;
2. explore a sub-set in which active phase duration could be determined, mainly from data available since 1990;
3. assemble evidence of surge cycle length or recurrence intervals for glaciers with two or more established surges;
4. describe the range of surge-modified glacier behavior as introduced above.

## Results

In all, 223 surge related phenomena were identified and mapped in the study area (Fig. 2), comprising glacier basin areas of 8014 ± 280 km^2^. There are 163 surge-type glaciers (6269.7 ± 219 km^2^), 58 surge-like (1464.3 ± 51 km^2^), and 2 examples of surge-modified glaciers (280 ± 10 km^2^) (Supplementary Table S2). The evidence derives largely from ablation zone features, but will affect the stability and mass balance of the whole connected glacier system. In the Karakoram itself, the count was 210 in affected basin areas of 7836.0 ± 274 km^2^. In the immediate neighborhood (i.e. Pamir Wakhan, Aghil mountains and Chang Chenmo) were 13 more examples, covering 177.7 ± 6.2 km^2^. The inventory suggests these glaciers involve ~50% of the entire glacier area surveyed, or almost half the total perennial snow and ice cover of the Karakoram, variously estimated at between 18,000 and 20,000 km^2^ within the Indus and Yarkand basins^5,39^.

Our study increases known numbers and diversity of surge events, and surge-related features. It includes some 100 surge-type glaciers not previously reported, and refines current knowledge of their distribution and movement characteristics (Supplementary Table S1). In the western part of the Karakoram 13 surge-type glaciers terminate below 3000 m, the lowest, Bualtar (ID 21), at 2280 m. Conversely, 10 surge-type glaciers in the Shyok and Shaksgam valleys terminate above 5000 m. There, the entire landscape is above 3600 m elevation and smaller precipitation may be a factor^40^. The count of surge-type and surge-like tributary glaciers was 69 or ~31% of the total (Supplementary Table S1). Individual large glaciers involve multiple surge-type tributaries. Sarpo Laggo possibly has seven; Panmah has at least six, while combining satellite and historical records gives Skamri and Hispar five each^5^. Each of the tributaries has a distinct and independent surge cycle. Tributary examples are more likely to be underestimated given their smaller size, and difficulties of observation in higher and steeper catchments. They are likely to generate surge-modified behavior in main glaciers where they connect.

More than half of the entire ice cover seems affected, which may seem an exaggeration. Other surveys estimate as many as total 13,757 glaciers in the whole Karakoram^41^. However, the vast majority are small ice masses, and in lesser offshoots of the highest, Mustagh Karakoram. In the latter, 11 glaciers are ‘large’ (40–75 km long) and almost 50 are intermediate (20–40 km), together comprising over two-thirds of the Karakoram glacier ice^5^. While the ten largest glaciers have no record of a surging main glacier most have surge-type tributaries (ibid). The results also show and reinforce evidence for even greater heterogeneity in surface displacement of Karakoram surge-type glaciers^19^ and implied dynamic instabilities.

### Characteristics of Surge-type glaciers

The survey adds to known three-phase classic surge cycles with high velocities and massive surface disturbances^16^ (Table 1). Many other cases are identified having more than three phases (Supplementary Table S2 and S3). In some the quiescent phase lacks a “stagnation phase”, and behavior is not readily differentiated from ‘normal’ glaciers. There are cases with well-defined but modest terminus advances and retreats, and some without any. At Bualtar (ID 21), Momhil (ID 41) and Braldu (ID 69), the active phases did not reach the terminus. A delayed advance eventually affected the first, and may yet occur in the other two. As detached tributaries of Panmah Glacier, Maedan (ID 94) and Shingchukpi (ID 95) advanced ~3 km in the active phase before reaching and stalling at the main glacier (Fig. 1c and f; Supplementary Table S1). Other complications arise with surge-type tributaries (Table 1 and Supplementary Table S2). At Panmah, despite the massive inputs of surged ice into and thickening of the main glacier since 1994, the terminus (ID 91) continued to retreat through 2016. It has been out of phase with climate since the mid-19^th^ century^5^.

In part, the classes identified are constrained by limited observations available and exploratory methodologies used. Most classic surge events are only known from ground observations, but going back to the mid-19^th^ century^4^. Unlike the Cross Correlation Feature Tracking (CCFT) satellite evidence, the exceptionally high velocities reported for active surges are based on terminus change and usually derived from total distances advanced, more rarely observed speeds. The maximum advance reported was for Kutiah Glacier (ID 29) 1953 surge, with 12 km in ~3 months^42^. The second largest was for the Hassanabad (ID 18) 1903 advance of 11.5 km in 2.5 months^43^, its speed said to exceed 150 m d^−1^. The fastest on record was at Yengutz Har Glacier (ID 24) in 1901 observed to advance 3.2 km in eight days^44^. These estimates have been questioned and are not well-constrained^45^. However, being based on terminus advances, they may well be less than the highest velocities up-valley in the main active surge. The maximum advance identified in our remote sensing analyses (1972–2016) was of ~3.5 km by west Chamshen Glacier (ID 179) in its surge of 2007–2013 (Supplementary Fig. S1). Obviously it was much less than the advance of Kutiah (ID 29)^42^ and Hassanabad glaciers (ID 18) and some other historical events^43^.

**Figure 3:**
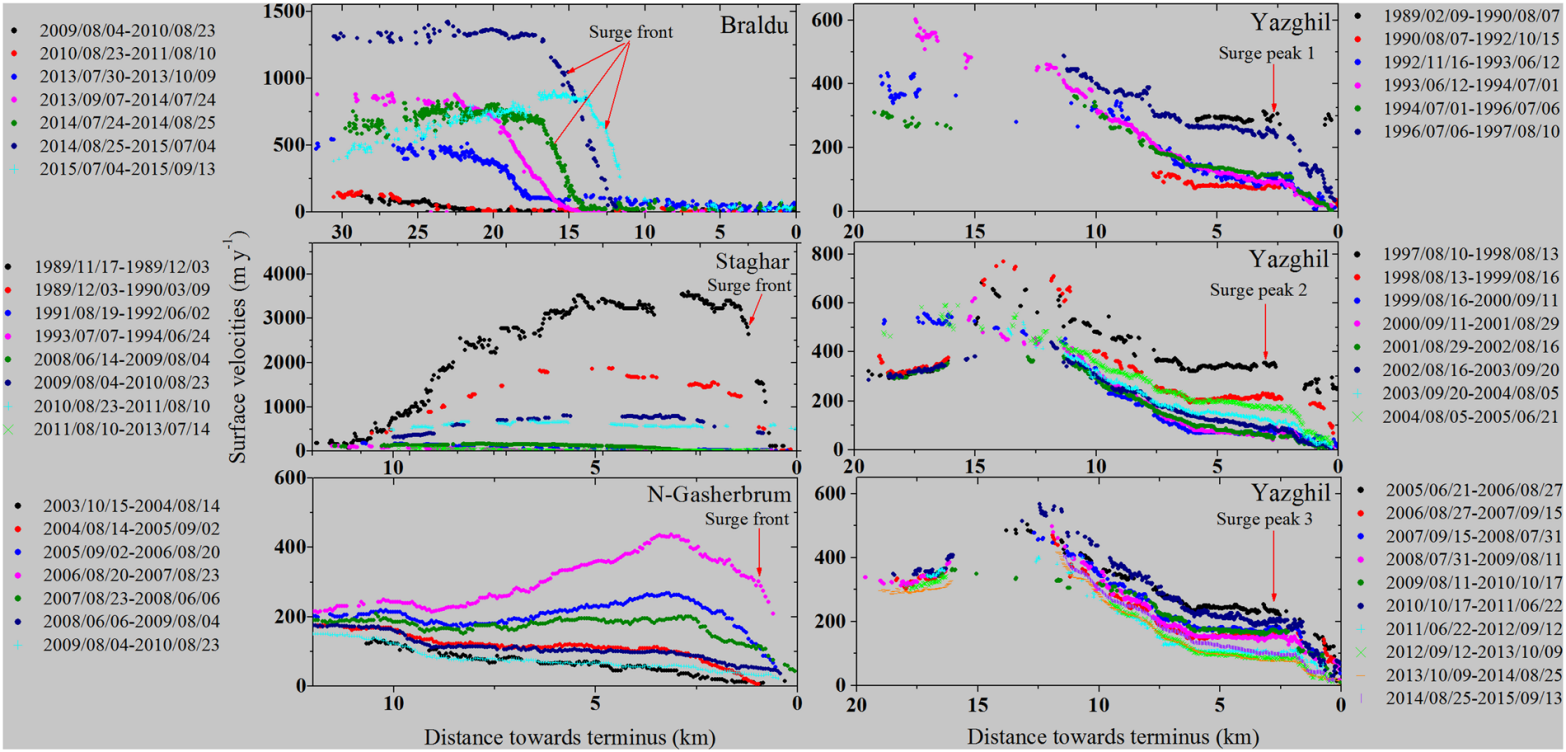
Surface displacement of four Karakoram surge glaciers (Braldu ID 69, N- Gasherbrum ID 155, Staghar ID 158 and Yazghil ID 42) based on Image correlation software (CIAS) (Kääb and Vollmer, 2000)^66^ and Heid and Kääb (2012)^68^ (http://www.mn.uio.no/geo/english/research/projects/icemass/cias/cias.sav). CIAS was originally written by M. Vollmer and A. Kääb (Kääb and Vollmer, 2000)^66^ and further developed by A. Kääb. Satellite image pairs used for automated feature tracking including the estimated uncertainty presented in Supplementary Table S8. Location of surface displacement of these glaciers in the Karakoram is presented in Fig. 2 (glacier ID).

CCFT has extended awareness of the extent, numbers and range of episodic, accelerated movements. They include velocity profiles and maxima much slower than the classic cases, and instabilities called surge-like behavior here. Velocities may be doubled or more, but not by one or two orders of magnitude^14,19^. There is a growing acceptance that such slower but sustained accelerations can be treated as surge-type or surge-like^5,16,17^. Thus, while markedly slower than classic surges, the events at Urdok (ID 157), Kyagar (ID 163) and Braldu (ID 69) were identified with an active surge front (Fig. 3 and Supplementary Figs S2 and S3), as earlier reports for the Kunyang (ID 38) tributary glacier^20^. Velocities in the movements of Little Chamshen (ID 181) and Dzingrulma (ID 204) were relatively low (0.1 km a^−1^), their active phases unusually long (~10 years) (Supplementary Fig. S4).

By comparison classic events now appear relatively infrequent, although they may be missed because surging is much faster and short-lived, especially where tributaries are involved. CCFT missed the highest velocities (10 m per day or more) observed on the ground during the Maedan, and Shingchukpi-Panmah tributary surges^30^. At Khurdopin the measured maximum CCFT velocities^46^ were significantly lower than short-term, main surge observations on the ground (K. Hewitt, unpublished field notes). This may arise through gaps without readily available imagery, or due to cloud and snow cover. The CCFT data may also be biased towards pre-, post-, and non-surge conditions but provide a much-expanded awareness of movement heterogeneity in the longer ‘quiescent phase’, largely neglected in the past.

Elsewhere, especially in Alaska, reports suggest active surges tend to commence in the winter months^47^. There are few reliable reports for the Karakoram, but the 1987 Bualtar (ID 21) surge started in January, the most recent Kichik Kumdan (ID 174) surge between December and April 1998. Older studies identified a winter surge in the latter, including a ~2.5 km advance between November 1935 and June 1936^48^.

**Figure 4:**
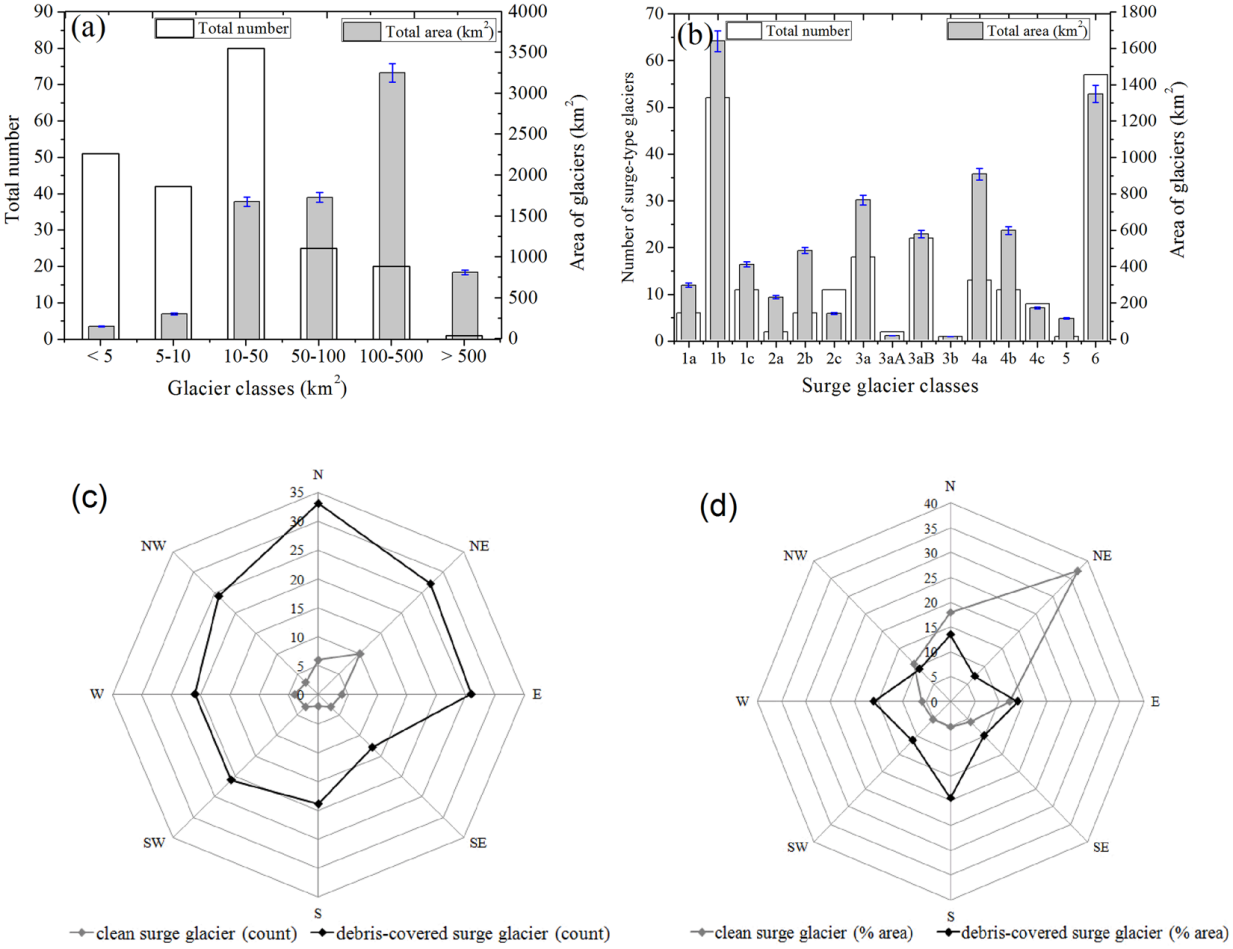
Surge-type glacier characteristics (**a**) Number and area of all glaciers assigned to  glacier classes, (**b**) fifteen surge glacier classes (see Supplementary Table S2) and distribution by numbers and area (km^2^),(**c**) orientation and numbers of clean and debris-covered surge glaciers and (**d**) orientation and area of clean and debris-covered surge glaciers (percent).

The largest surge-type glaciers with total basin areas of 2349 ± 82 km^2^ are found in the classic surge class ‘1’, with a maximum in sub-class Svalbard-type ‘1b’ (1639.5 ± 57 km^2^) (Table 1; Fig. 4; Supplementary Table S2). Some studies have suggested surge-type glaciers in the Karakoram region, as in Alaska and Svalbard, tend to be the longer and less steep ice masses^49^. Our work reveals no such correlation with glacier length or slope. The areas of some 50 surge-type glaciers are less than 5 km^2^ (Fig. 4).

Previous work has emphasized the importance of supraglacial debris covers, typical of the region’s glaciers^1,14,34,35^. Some 187 of the glaciers identified are heavily debris-covered, and 36 are relatively clean. In 94 cases, supraglacial debris is concentrated at medial, contorted and looped moraines. For all surge classes identified, the debris-covered area is 933 ± 30 km^2^ or ~11.6% of the total glacier area. Debris covers tend to be greatest in ablation zones of southerly oriented glaciers, or 19.5% of total glacier area. The least debris-covered (6.5%) are of northeast orientation (Fig. 4; Supplementary Fig. S5). In general, across the western Himalaya, debris-covered ice decreases from southwest to northeast^50^. Debris-cover also reflects the extent of ice-free and heavily avalanched headwalls, more common on south facing slopes. All surge-type glaciers, but also most others in the region, are predominantly avalanche-fed^5^. Among sub-regions of the Karakoram, the Hunza valley has extensive, heavy debris covers, the Shyok basin much smaller covers usually restricted to contorted and looped medial moraines. There are exceptions, however, like the largely clean Pasu and Mallangutti glaciers in Hunza and in the northeast, the heavily debris-covered Urdok in Shaksgam valley. Debris covers may be massively redistributed during and after an active surge. Otherwise, they seem mainly to reflect conditions affecting avalanche debris content.

### Surge phase duration

**Figure 5:**
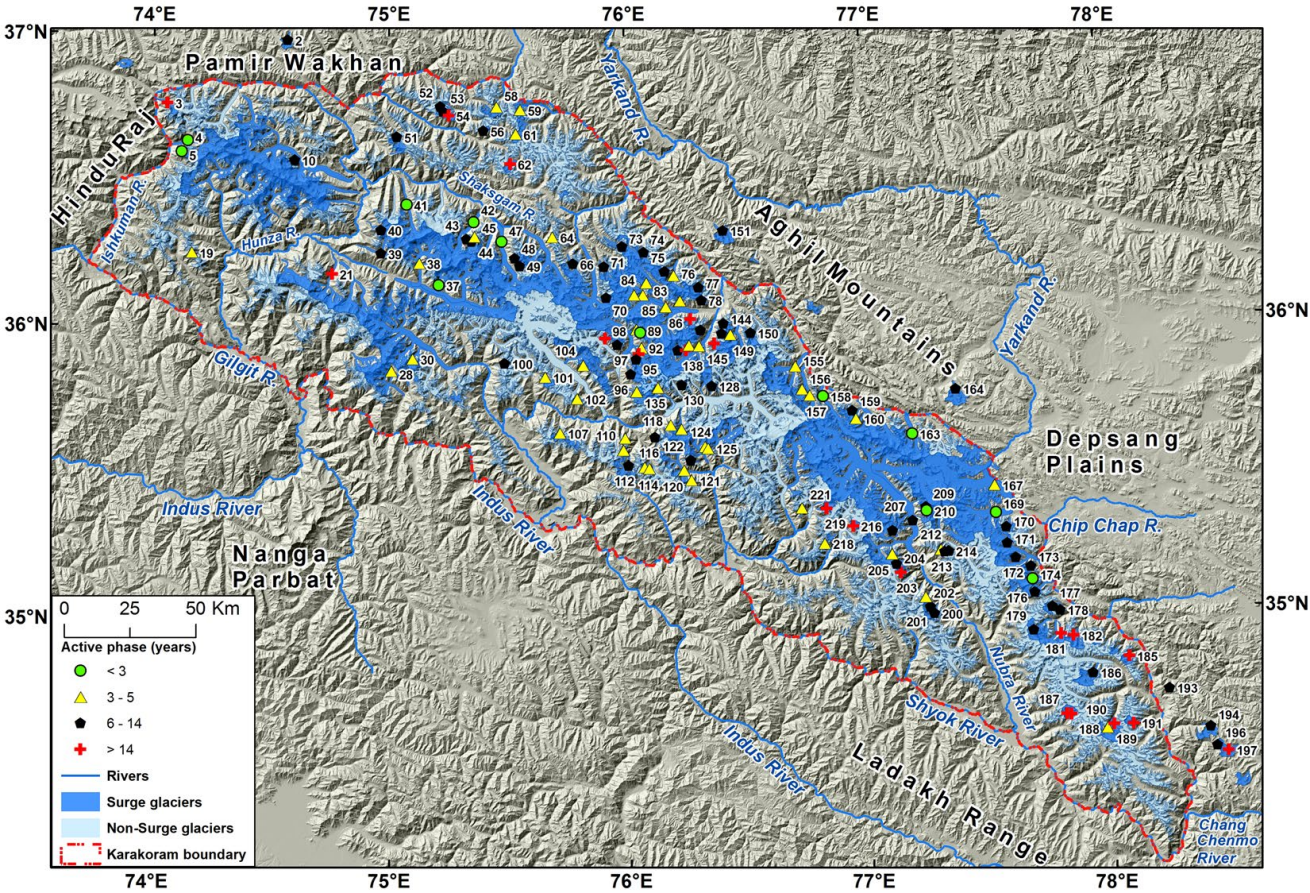
Duration of active phase (years) in the Karakoram and neighbourhood area. The map is generated by the open-source datasets using ArcGIS version 10.0 (http://www.esri.com/software/arcgis/arcgis-for-desktop). The hill-shaded background in this figure have been generated by using the Shuttle Radar Topography Mission (SRTM) data set (90 × 90 m) provided by the CGIAR Consortium for Spatial Information (http://srtm.csi.cgiar.org/). The glacier outlines were modified (details as given in methods) from the Randolph Glacier Inventory (RGI 5.0) (Pfeffer *et al*.)^72^ (http://www.glims.org/RGI/00_rgi50_TechnicalNote.pdf). Karakoram boundary is based on classical work by Royal Geographical Society and Survey of India^62^. See for more details (e.g. Ids) for active phase in Supplementary Table S1.

Active surge phases involve the largest, most concentrated transfers of ice mass, and are of special interest for fast flow dynamics. In line with other recent studies^35^ our data show they can last from months to over 15 years (Fig. 5). Older work based on ground observations only reported classic type surges with active phases of weeks to months. This suggests observers missed the slower and more drawn out cases evident from CCFT data^31–33,42–44,51^.

For 12 glaciers we generated 120 automatic surface displacement data sets at an annual scale and, for 13 others, manually measured velocities. Active phase movements varied from 4 km a^−1^ to 0.1 km a^−1^ (Fig. 3 and Supplementary Figs S2, S3 and S4). Many showed relatively high displacements (>1.0 km a^−1^). ‘Classic’ active phases in main glaciers generally lasted ≤2 years. In all, eight glaciers had active phase durations of ≤2 years, including Kichik Kumdan (ID 174), Hassanabad (ID 18), Karumbar (ID 4), Kutiah (ID 29) and South Rimo (ID 169). There were 37 lasting ≥10 years (Fig. 5; Supplementary Table S1). Peak velocities, where they could be separated out, occurred mostly in summer months^19^. However, in contrast to a previous study^19^, the highest displacement of Staghar (ID 158) Glacier occurred during winter months of 1989 (Fig. 3).

### Surge Cycle Recurrence Intervals

Where two or more active phases can be determined for a given glacier they offer a basis to establish recurrence intervals. If consistent between events they could help predict the timing of future surges.

**Figure 6:**
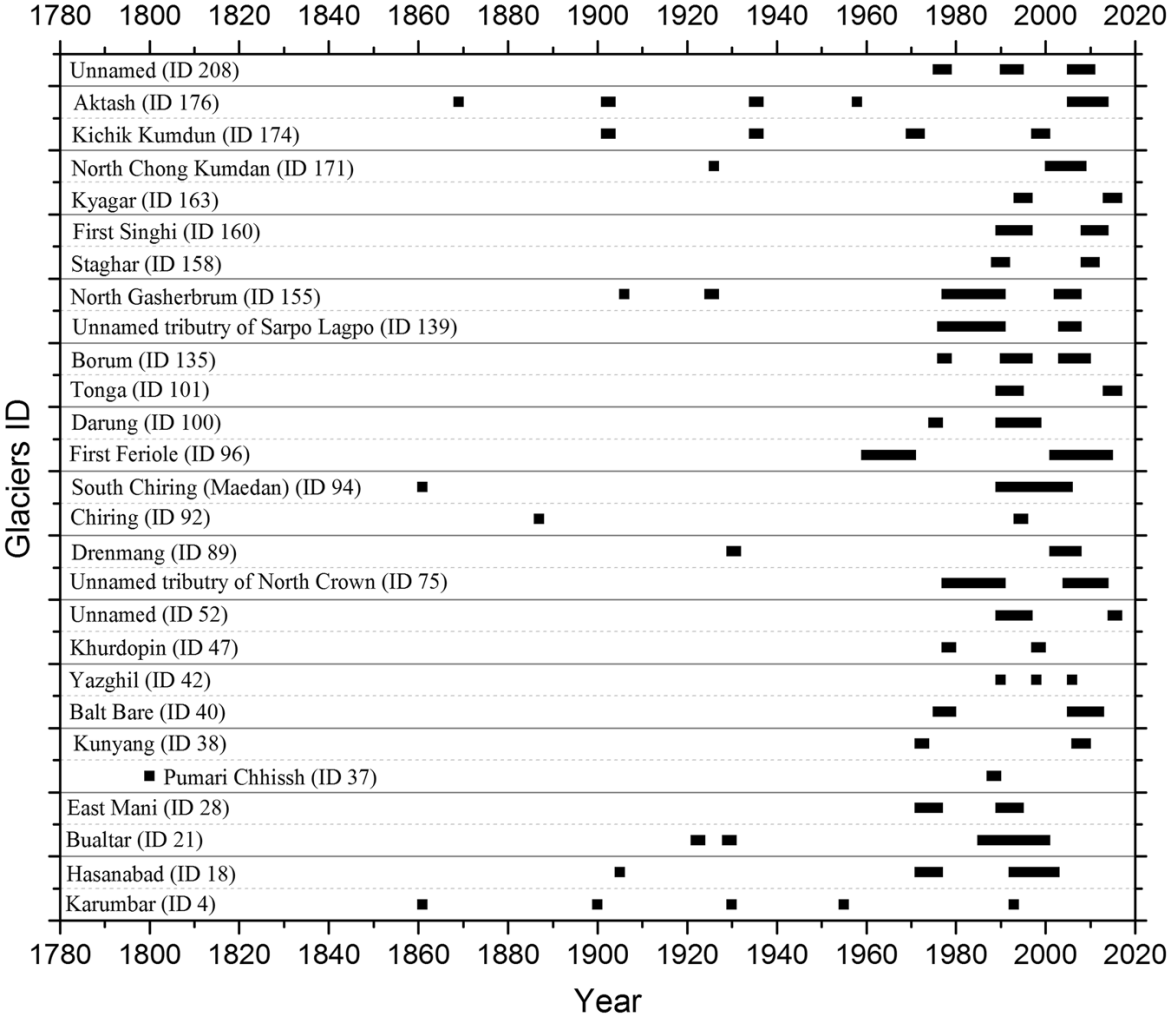
Repeat cycles of surge-type Karakoram glaciers. Length of dark black lines represents surge duration. Database of repeat surge of glaciers are based on satellite images (1972 onwards) and from the previous historical archives since 1840s (Supplementary Table S4). Location of glaciers (ID) is presented in Fig. 2.

By comparing earlier literature and satellite data from 1972 to 2016, we identified two or more surges for 27 glaciers, 9 not previously reported (Fig. 6; Supplementary Tables S4 and Fig. S6). The most extensive records of repeat cycles are for the Shyok and Ishkoman valleys, respectively in the far eastern and western parts of the Karakoram. The data suggest Aktash (ID 176) in Shyok valley, and Karumbar (ID 4) in Ishkoman valley have surged as much as five times in two centuries (Supplementary Table S4). Mason (1930)^3^ deduced what would now be termed surges and their recurrence for ten glaciers. We could confirm his dates for three cases but not the remainder (Fig. 6). During the 20^th^ century Kichik Kumdan Glacier (ID 174) surged 4 times, in 1902–1903; 1935–1936; 1970–1972 and 1998–2000. Three recurrence intervals cluster between 34 and 32 years, but the most recent was 26 years. Three surge peaks at Yazghil Glacier during 1990, 1998 and 2006 suggest a cycle or recurring instability threshold of 8 years (Fig. 3). However, an implied surge for 2014 has not occurred to the end of 2016.

Surge intervals established through earlier historical records are between 30 and 90 years^3^. Some newly discovered ones are shorter. Borum Glacier (Dumordo valley) underwent marked accelerations to active phases during 1991–1996 and 2004–2009. It also began to surge in 1977–1979 but the full extent is unknown due to lack of imagery until 1988. One unnamed glacier (ID 212) near the North Terong Glacier (ID 211) has surged three times between 1976 and 2015 (Supplementary Fig. S6 and S7). Two active phases of First Singhi Glacier (ID 160) were found during 1991–1996 and 2009–2013. The observations also suggest quiescent phases of around 13 years.

Bualtar (ID 21) is unusual in having pairs of active surges, in 1922–23 and 1929–30, and in 1987 and 1990. It creates a curious case of alternating short and long quiescent phases^5^. The explanation is unclear but comprises another glacier-determined rhythm that complicates tracking of climate relations.

In all, great variability is evident in surge cycle intervals, from as little as a decade to over a century. Given the patchy nature of visits and reportage, especially prior to the 1970s, an even more varied picture seems likely. To date, with possible exceptions for some classic surge glaciers like Karumbar (ID 4) and Kichik Kumdan (ID 174), the evidence seems unreliable for predicting the exact timing of future events, and does not preclude complications that could reflect as yet undetermined effects of climate change.

It should be noted that the largest glaciers (e.g. Rimo^52^) are all smaller now than their Little Ice Age maxima, but individual thicknesses and terminus fluctuations were out-of- phase then, and since^5^. For almost a decade, between 1989 and 1998, surface velocities at 12 km above the terminus of the largest Karakoram glacier, Siachen (ID 207), fluctuated from 350 m a^−1^ (1995–1996) to 120 m a^−1^ (1998–1999) (Supplementary Fig. S3). The terminus advanced by ~250 m. Possibly this was climate-driven^53^, but the passage of an active surge initiated before 1989 cannot be ruled out, perhaps a mini-surge, or a tributary surge missed due to gaps in satellite coverage.

### Surge Length and Peak Velocity

Surge length, the distance covered by an active surge, is another measure that may reveal movement heterogeneity. In all, the results show great variety and no consistency in surge lengths (Supplementary Fig. S6), including repeated surges (Fig. 6). Of eight glaciers, in five (ID 42, 75, 96, 135, 212) the most recent event had a shorter surge length (Supplementary Fig. S6). In three others recent surge length exceeded the earlier ones (ID 139, 158, 160). In seven cases (ID 4, 21, 47, 155, 171, 174, 176) latest surges were shown to be less than historical maxima^5^.

Where identified, peak flow velocities for given glaciers have varied between events. Those determined for Staghar Glacier (ID 158) in Yarkand basin were at least twice as large in the 1989–1990 surge, compared to 2009–2011. A similar reduction occurred in active surges of the Khurdopin Glacier (ID 47)^46^. Velocities at Yazghil Glacier (ID 42) were greater in 1997–1998 than 1989–90 and 2005–2006 (Fig. 3). However, in available satellite imagery the onset and termination of active surges and their maximum velocities may well be missed.

## Discussion

The evidence presented, although unlikely to identify all cases, adds considerably to the numbers of surge-type glaciers previously known^5,14,15,34,35,52^. What stands out is the diversity of surge-types, surge-like instabilities or surge-modified behavior (Supplementary Tables S2, S3 and S5). The latter also shows the need for greater attention to the quiescent phase, which the majority of surge-type glaciers are in at any given time. Uncertainties in parts of the data are acknowledged and further research will surely establish more precise estimates for surge dimensions. Nevertheless, the findings confirm that surging and related instabilities are pervasive, possibly dominant factors in the behavior of Karakoram ice. We suggest this has a unique bearing on efforts to identify how global climate change affects the region. Terminus advances and retreats were formerly the only evidence from the Karakoram and are still a basis for many claims about negative or positive mass balance^45,54^ (Supplementary Fig. S8). The glacier surges inventory generated in the present study reveal complications that put such evidence in doubt (Supplementary Table S1). Many of the Karakoram glaciers not identified with surging may well have greater and relatively direct responses to climate, but an unknown number of them may prove to be surge-type or affected by surges. Mainly we must stress how, and how far, the behavior of surge-type glaciers departs from climate-driven responses. Two main concerns arise; the range and classes of surge-related phenomena, and their implications for mass balance.

**Figure 7:**
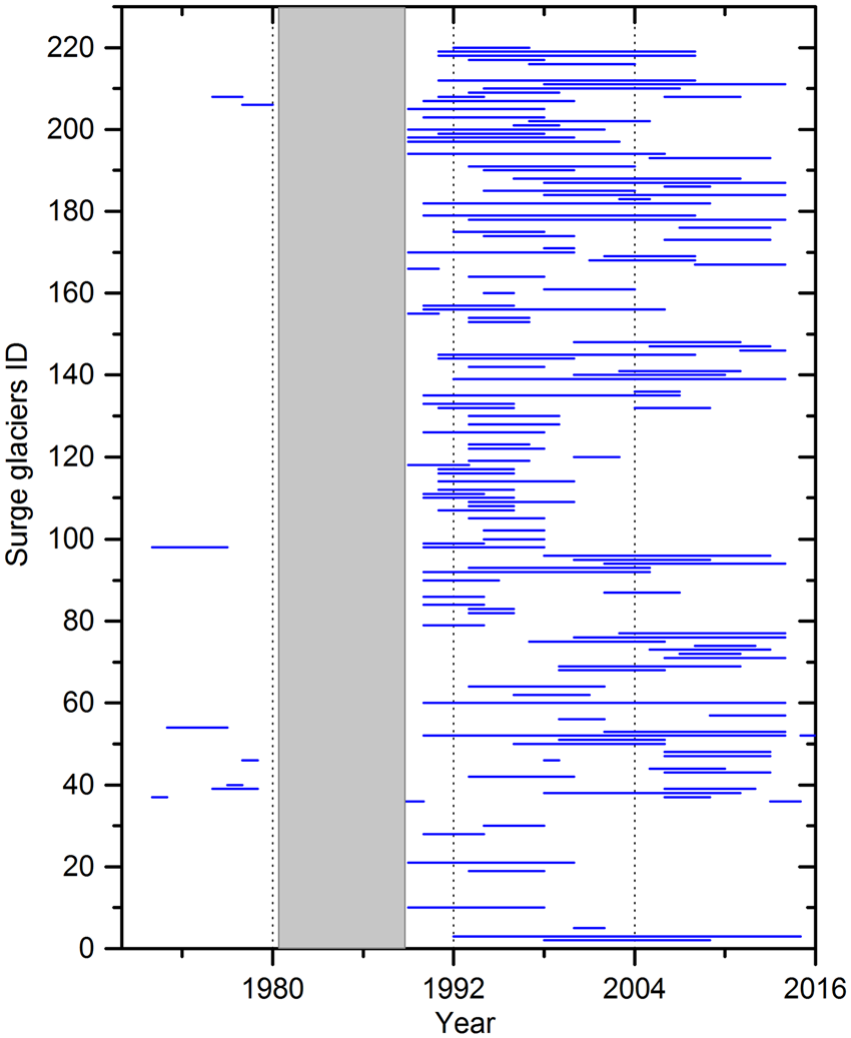
Duration of surge activity in the Karakoram and neighboring areas from 1972 to 2015 based on Landsat and ASTER satellite data. Length of blue lines represents duration of active surges. More details are presented in Supplementary Table S1 and location of glaciers ID is presented in Fig. 2. No suitable Landsat satellite images were available between 1981 and 1988 (gap area) for study area and presented by gray colour.

It seems useful to combine and compare our findings in a revised classification (Table 1). Some studies explain newly discovered surge events as results of climate warming^5,20^. That may yet prove to be so. However, our evidence highlights how surges and surge-related behavior intervene in glacier responses, with a large and varying potential to block, over-ride, or reconfigure fluctuations in climate trends, especially through mass balance^29,47^. If each surge-type glacier is indeed out-of-phase with others, this could substantially explain movement heterogeneity^19,20^. The timing and recurrence of surge activities, velocity fluctuations (Fig. 3), surge duration^55^ (Figs 5 and 7) and length (Supplementary Fig. S6) will be present in the observations used to track glacier change^16^. This emerges from examining how surging intervenes in glacier mass balance.

Mass balance is generally regarded as more reliable than terminus changes and more fundamental to glacier-climate relations^56^. Unfortunately, there are very few actual measurements of mass balance for Karakoram glaciers and none for surge-type^5^. Most of the studies in the Karakoram used multi-temporal DEMs to estimate geodetic mass balance^8–11^ along with point elevation changes derived from ICESat/GLAS data^12^. Yet, the basic principles on which mass balance studies rely make clear how surge-type and surge-modified (Supplementary Table S3, S5) behaviors depart significantly from conventional mass balance relations which have been established for non-surge glaciers^57–59^.

The ‘reservoir’ and ‘receiving’ zones that dominate the budgeting of inputs and outputs in surge-type glaciers are not synonymous with conventional accumulation and ablation zones^56^. The ‘reservoir’ as indicated by collapsed, crevassed and break-out areas during surging, involves only part of the upper glacier area (Fig. 1a). In many known cases, surge sources involve the upper ablation zone, as at Chiring (ID 92) Glacier in 1994. Some lie entirely within the ablation zone, as at Bualtar (ID 21) in 1986^37^. In these cases instabilities generated by the surge led to readjustments in the upper reservoir zones for years afterwards, a variety of post-surge, surge-modified effect on mass transfer in the early quiescent phase. Of course, the snowfall that feeds the build up, and ablation in the receiving zone, *are* driven by climate conditions. However, outcomes are not budgeted by the interplay between accumulation and ablation rates, but by internal glacier dynamics and the surge cycle.

Developments in the lower, receiving zone diverge from those in the reservoir zone. Most of the time they are of opposite sign. There is a relatively brief, catastrophic exchange, not a sequence of systematically budgeted adjustments. In the long quiescent phase, the reservoir and receiving zones are out-of-phase, hence the value of the three stage model introduced above. Except during and immediately after the active surge, the former grows, building towards the next surge while the receiving zone stagnates or retreats^16^. Surging carries large volumes of ice mass through the system in just a few months or years^16,18^. In classic events and some others, it causes large terminus advances. However, rather than evidence for positive mass balance these prefigure decades of enhanced ablation losses, again regardless of climatic trends.

Surge rhythms depart from those conventionally defined by systematic, up-glacier adjustments and the vertical mass balance profiles typical of mountain glaciers^5,56^. This extends the complications generally identified with non-steady flow in glaciers^58^. Important concepts such as balance velocity, mass balance gradients, and Equilibrium Line Altitudes (ELAs) are absent or unspecified^56,58^. Surge length and duration, or related glacier thickness, do not track climate influences. At any given time, and most of the time, these glaciers are in the ‘quiescent’ phase, typically neglected but also implying long term disconnects between climate influences, mass balance and terminus fluctuations. In the short and medium term–10 s to 100 s of years–the glaciers in quiescent phase will have quite limited sensitivity to climate fluctuations. Surge cycles remain out of phase with each other and climate trends. Longer term harmonization with secular climate fluctuations is conceivable, perhaps inevitable, but as a highly lagged, centuries-long statistical process. Then again, our data do not exclude the possibility that as much as half of the glacier covers may not comprise surge-types, and be responding normally to climate change^59^. However, the concentrations of surge-type glaciers are largely in the highest, Mustagh Karakoram, where advancing glaciers have been reported recently, and an apparent slight increase in total ice mass^1,5–15,34,35,52^. Research to assess such relations in the Karakoram remains to be done.

Finally, the presence of surge-type and surge-related phenomena depend in some way upon Karakoram climate and other conditions that appear exceptional within High Asia. The Mustagh Karakoram combines the greatest extent of extreme elevations and great relief, the greatest glacier cover and most of the largest valley glaciers outside higher latitudes^5,15^. Glacier basins have exceptional steepness and vertical range of rock walls, wind redistribution of snow and avalanche-nourished ice^5,60^. Unlike the rest of the Himalaya, or Hindu Kush and westwards, the Mustagh Karakoram has large and nearly equal inputs of snow in winter and summer, likely to complicate ice thermal regimes^5^. Avalanche nourishment indicates heavy debris loads within as well as on the surface of most glaciers, potentially a source of abundant deformable bed material.

The Karakoram glaciers are definitely not ‘disappearing’ at this time, which seems good news for the millions dependent on them. However, current developments are not without risks. None of this means the climate is not changing and in ways that can adversely affect the Karakoram cryosphere and those dependent upon it. Recent trends do not preclude future, perhaps catastrophic depletion of Karakoram ice in response to climate warming. Where they occur, surge events can adversely affect mountain communities. Surge-type glaciers are common causes of glaciers interfering with and damming of rivers^3,21,22,33^. Glacier lake outburst floods (GLOFs) create extreme dangers for downstream communities and infrastructure^3,21^. In all there is a need for innovative research and monitoring in the Karakoram to address the influences of so many surge-type glaciers and future roles of such diverse, surge-related behavior.

## Methods

### Satellite data

Multi-year and continuous observations are required for adequate monitoring of active-phase and repeat cycles of surge-type glaciers. In some cases surging may last just a few months, in others several years. Major impediments such as seasonal snow cover preclude continuous observations at a monthly scale for this region. Satellite images during August to November and with limited cloud cover were mainly used for feature identification and we report the lengths of active phases on an annual scale.

The longest program series, such as Landsat (sensors MSS, TM, ETM+ and OLI), available since the early 1970s, have proved suitable for mapping these glaciers^14,15,19,20,34,35,52^. We used Landsat MSS data from 1972 to 1980 (61 scenes) and Landsat TM, ETM+, and OLI (329 scenes) data from 1989 to 2016 (Supplementary Table S6). The scenes were obtained from USGS (United States Geological Survey; http://earthexplorer.usgs.gov/). High resolution satellite images from Google Earth were also consulted to help identification of surge-type glaciers and related features (e.g. potholes, intense crevasses). 3D visualisation of glaciers in Google Earth satellite images also helped to understand changes in reservoir and receiving zones when surrounding stable features carefully used as reference (e.g. lateral moraines, nunatak). The Landsat 7 ETM+ scenes are affected by scan line errors since 2003 except in middle portion of scenes (~22 km wide) due to permanent failure of the scan line corrector (SLC)^61^. Therefore, we used ASTER data (170 scenes) from 2000 to 2013 giving complete coverage at an annual scale (Supplementary Table S6). Many Landsat MSS scenes are affected by severe distortions like shifted lines in scenes. Therefore, MSS scenes were used mainly to compare surface morphology with 1990s TM scenes^14^ (e.g. Khurdopin Glacier^46^). Identification of repeat glacier surges combines Landsat scenes and historical sources.

Various studies have also covered a limited number of surge-type glaciers and sub-regions of the Karakoram^14,15,19,20,34,35,52^. Some include surrounding areas like the Aghil, Chang Chenmo, Nanga Parbat and Ladakh mountains in the Karakoram^6,14,34,52^. We utilize the Karakoram boundary based on the Survey of India definition^62^ which excluded these adjoining areas (Fig. 2). However, we also mapped surge-related phenomena in the Karakoram and some neighboring parts of the Wakhan Pamir, Aghil and Chang Chenmo Mountains to compare previous studies. Our verified Karakoram area covers ~44,500 km^2^ with an elevation range from ~1250 to the summit of K2 at 8611 m a.s.l.

### Surge-type glaciers inventory

We used glacier outlines from the Randolph Glacier Inventory (RGI 5.0) [www.glims.org/RGI/] as reference data for mapping surge-type glaciers^9^. The glacier outlines are in polygon shape file format and cover the whole Karakoram glacier region. The RGI global glacier inventory (RGI 5.0) has included glacier outlines with mapping uncertainty of < ± 3.5% for Shyok basin, eastern Karakoram^52^. We visually checked outlines of surge-type glaciers using recent Landsat OLI images (2013 to 2015). At many places glacier outlines were found to include seasonal snow cover and rocky outcrops. These were updated manually^9,52^. Outlines of debris-covered glacier fronts were also updated manually by visual interpretation of high resolution Google Earth satellite images^63^.

We mainly used four diagnostic criteria for identification of surge glaciers where no actual surge or only limited disturbances are recorded:

1. morphological and localised surface patterns such as moraine ‘loops’ and ‘tear-drop’ forms^16^; breach lobes, trim-lines and sheared-off tributaries^30^; heavy crevassing of formerly much smoother glacier; potholes and shear margins^14^;
2. Terminus advance^64^ and rapid retreat unrelated to surrounding glaciers^18^;
3. Terminus thickening with intense crevasses and bulbous terminus^14^ and
4. Acceleration of ice, confined here to at least a doubling pre-event velocities, affecting a given region of ice or moving progressively down-glacier^5^.

There may be evidence of local thickening and over-riding of ice-margins, or ‘surge bulge’^16^ (Fig. 1f).The criteria and features used depend partly on previous inventories but extend the numbers of surge-type glaciers recognized^14,15,19,20,34,35,49,52^ (Supplementary Table S7). We propose a three-part classification scheme and several sub-classes of recognized surge-types. Surge -type includes typical ‘classic’ main glacier and tributary surge activity, including Alaska- and Svalbard types and sub-types of ‘amended ‘classic’, with reduced or increased cycle phases. A further set is recognised only from ‘surge-diagnostic’ features outlined above (Supplementary Table S3).

Surge-modified refers to disturbances triggered by surging in adjacent non-surge ice, post-surge impacts on adjacent ice areas, or adjustments between tributary surge ice and main glacier ice^30^. These are observed to continue for years or decades after the surge event itself. They can be identified in surface ice patterns and morphology, debris cover, and a range of velocity disturbances. Such disturbances are initiated or driven by active surges, but occur in ice after or beyond actual surging.They became especially evident in recent recognition of surge-type tributaries, notably their impacts in large glaciers such as Panmah, and Hispar^5^. Adjustments of main glacier and/or tributary glacier ice were observed for more than two decades after the active surges, indirect consequences of surging that also do not reflect climate. On the one hand, surge-modified behavior does not create such sudden, rapid or extreme developments as active surges, certainly not compared to ‘classic’ surges. On the other hand, they can continue to affect as much or more ice, or for much longer periods and in ice otherwise exempt from surging, or that did not surge in the main event. More details on surge related classification and examples can be found in Supplementary Tables S2, S3 and S5.

### Active phase estimation

Surge phase duration was estimated from displacement of surface features on successive satellite images. Both manual and automatic surface feature tracking methods were used to compute surface flow velocity, and in several sequential images to counteract changes in snow extent, cloud cover and illumination^65,66^. A normalized cross-correlation (NCC) algorithm was used to drive multi-temporal surface flow velocity from two successive pairs of Landsat or ASTER images using Image correlation software (CIAS)^66–68^. USGS provided orthorectified Landsat TM/ETM+/OLI scenes. The planimetric shift found in all Landsat images at an individual glacier scale, was visually checked and coregistered where required, using the projective transformation algorithm of Erdas Imagine^52^. We used multiple satellite images covering both active and quiescent phase surface flow velocities. For this, search window size ranged from 30 × 30 to 250 × 250 and a reference windows size of 10 × 10. Filtering and cleaning removed spurious surface displacements. We excluded ≤ 0.6 correlation coefficient from the glacier flow data set, as suggested by Redpath *et al*.^67^. Directional filtering was also used to eliminate spurious displacements. Finally, velocity vectors were visually evaluated on satellite images and any remaining false displacements removed. All the accepted surface displacements were then converted to an annual scale^19,20^. The following equation, suggested by Quincey *et al*.^19^ was used to estimate uncertainty in glacier displacements

1 $$\sigma =365\frac{({{\rm{C}}}_{{\rm{pix}}}+{{\rm{C}}}_{{\rm{match}}})\,{\rm{\Delta }}{\rm{x}}}{{\rm{\Delta }}t}$$

where C_pix_ is the uncertainty in co-registration in pixels (p), C_match_ is the uncertainty in the matching algorithm in pixels (p), Δx is the image resolution in meters, and Δ*t* is the time interval between the image pair in days. We used 0.5 p values for C_pix_ and C_match_ as proposed by Quincey *et al*.^19^ (Supplementary Table S8).

Selection of surface features (e.g. looped and wave-like/folded moraines) was based on clarity in image pairs and distribution across ablation zones. A polyline was digitized using ESRI ArcGIS from the feature’s starting point on image 1 to the same point on image 2. The length of the polyline (i.e. feature displacement) was calculated for both active and quiescent phase velocities^65^.

Velocities could only be estimated for the snow-free ablation zone. Frequently snow-covered accumulation areas suffer a lack of distinctive or repeated surface features. Landsat Level 1T images have been reported to have one pixel accuracy and feature tracking mapping^65^. This was also carried out to an estimated accuracy of one pixel, thus resulting in a total maximum uncertainty of two pixels between image pairs^65^. However, in several satellite images seasonal snowfall and cloud cover hampered movement tracking of surface features even in ablation zones. Terminus advances, where present, were also used to help determine active phase duration^34,52^.

Glacier surge fronts are usually uneven and changes in terminus position irregular. Thus, glacier lengths were measured using a glacier length tool developed for the ArcGIS 10.0 software^69^. Since this tool only takes the frontal part of glacier as input, we mapped these from the Landsat and ASTER satellite images, not the entire glacier outline^69^. The glacier length tool divides the front into points spaced e.g. 15 m apart and calculates the mean distance to a reference point placed up glacier (Supplementary Fig. S9). Using the same reference point for all years enables a direct comparison of changes in front position. Some other characteristics such as supraglacial ponds or creeks helped determine the most likely position of the termini^52,70^.

We estimated the errors in length change based on an equation for multi-temporal length measures of the glacier front position proposed by Hall *et al*.^71^.

2 $$e=\sqrt{{({\rm{a}}1)}^{2}+{({\rm{a}}2)}^{2}}+{{\rm{E}}}_{{\rm{reg}}}$$

where; e = error in length change, a1 = pixel resolution of imagery 1, a2 = pixel resolution of imagery 2, E_reg_ = horizontal shift

We employed at least half pixel as horizontal shift between pair of satellite images^70^. Consequently, the error was estimated for Landsat TM, ETM+ and OLI images as follows:

$${\rm{e}}=\surd [{(30)}^{2}+{(30)}^{2}]+15=57\,{\rm{m}}$$

The uncertainty was 29 m for a pair of ASTER images, 57 m for pair of Landsat TM, ETM+ and OLI, 152 m for pair of Landsat MSS, and 124 m in the case of length estimation from Landsat MSS and TM images. These uncertainties are within the range of previous estimates (Hall *et al*.)^71^. Glacier length change was computed for 111 surge-type glaciers in the study area. Out of this, only seven were considered for length change from Landsat MSS images, mainly to study repeat cycle of surges.

## Electronic supplementary material

Supplementary information
