# Supplementary material for: Surge-type and surge-modified glaciers in the Karakoram
Source: Sci Rep. 2017 Nov 13;7:15391. doi: 10.1038/s41598-017-15473-8 (PMC5684366; doi:10.1038/s41598-017-15473-8)
Supplement: Supplementary file 1 — Supplementary information [file 41598_2017_15473_MOESM1_ESM.docx]

**Supplementary Information**

**Surge-type and surge-modified glaciers in the Karakoram**

**R. Bhambri^1*^, K. Hewitt^2^, P. Kawishwar^3^, and B. Pratap^4^**

[1] {Centre for Glaciology, Wadia Institute of Himalayan Geology, 33 GMS Road, Dehradun- 248001, India}

[2] {Department of Geography and Environmental Studies, Wilfrid Laurier University, Waterloo, Canada}

[3] {Chhattisgarh Council of Science and Technology, Vigyan Bhavan, Vidhan Sabha Road, Daldal Seoni, Raipur (CG) 492014, India}

[4] {National Centre for Antarctic and Ocean Research, Vasco-da-Gama, Goa 403 804, India}

**^*^**Correspondence to: Rakesh Bhambri ([rakeshbhambri@gmail.com](mailto:khewitt@wlu.ca))

In this document, we provide information on the satellite data used in the present study and surge glaciers inventory, surface displacement, cumulative length change of surge-type glaciers during the repeated surge, surge-type glaciers classification scheme with selected examples as well as details on repeated cycles of surge glaciers. We also provide the error estimation of glacier surface displacement.

**1. Supplementary Tables**

Supplementary Table S1 Surge glaciers inventory of the Karakoram. Details on types of ^1^Surge type glaciers are presented in Supplementary Table S2, S3 and S5. L1 and L2 represent Latitude and Longitude respectively. M1 and M2 represent minimum and maximum elevation respectively.

| ID | GLIMS ID | Name | Glacier type | Region | L1 | L2 | Length (Km) | Area (km)^2^ | M1 (m) | M2 (m) | ^1^Surge type | Surge years | Surge period | Debris  (%) | Debris type | Front advance (m) | Surge characteristics | References |
| --- | --- | --- | --- | --- | --- | --- | --- | --- | --- | --- | --- | --- | --- | --- | --- | --- | --- | --- |
| 1 | G073855E36900N | Unnamed | Main | Hindu Kush Range | 36.900 | 73.855 | 8.0 | 12.0 | 4359 | 5850 | 4a | - | - | 8.4 | Moraine | - | Looped moraines and crevasses. | Present study |
| 2 | G074567E36979N | Hark | Main | Panjshir, Afghanistan | 36.979 | 74.567 | 6.7 | 12.5 | 4733 | 5793 | 1b | 11 | 1998-2009 | 0.0 | Clean ice | - | High surface velocity, ~1.5 km advance in glacier terminus during 1998-2013, crevasses and deformed moraines. | Present study |
| 3 | G074057E36762N | Chillinji | Main | Karakoram | 36.762 | 74.057 | 10.9 | 14.0 | 3316 | 6602 | 6 | 23 | 1992-2015 | 19.1 | Ablation zone | - | ~0.3 km advance in glacier terminus sometime between 1993 and 2015, heavily crevasses, thickening of terminus and deformed moraines. | Present study |
| 4 | G074144E36634N | Karumbar | Main | Karakoram | 36.634 | 74.144 | 20.7 | 82.0 | 2874 | 7109 | 1a | 1 | 1993 | 8.8 | Ablation zone | - | Surge blocked river in 1955. Surge which started in April 1993 caused glacier advance of 7-10 m per day by June 1993 (Ref. 25). | 9, 21, 23, 24, 25, 30, 34 |
| 5 | G074119E36595N | West Karumbar | Tributary | Karakoram | 36.595 | 74.119 | 6.5 | 5.7 | 3351 | 6079 | 3aA | 2 | 2000-2002 | 20.1 | Ablation zone | 580±57 | ~0.6 km advance in glacier terminus during 2000-2002, crevasses and lobe-shaped terminus. | Present study |
| 6 | G074404E36770N | Kuk-KI Jerab | Main | Karakoram | 36.770 | 74.404 | 17.8 | 41.4 | 3610 | 6880 | 4a | - | - | 37.9 | Ablation zone | - | Looped moraine across ablation area. | 9 |
| 7 | G074583E36713N | Unnamed | Main | Karakoram | 36.713 | 74.583 | 4.0 | 3.4 | 4446 | 5858 | 6 | - | - | 5.6 | Moraine | 510±57 | ~0.5 km advance in glacier terminus during 1998-2014, heavily crevasses and thickening of terminus. | Present study |
| 8 | G074511E36700N | Unnamed | Main | Karakoram | 36.700 | 74.511 | 4.3 | 2.4 | 4582 | 5819 | 6 | - | Between 2002-2014 | 5.3 | Moraine | 439±57 | ~0.5 km advance in glacier terminus during 2002-2014 and heavily crevasses. | Present study |
| 9 | G074528E36650N | Yoksugoz Ice Flow | Tributary | Karakoram | 36.650 | 74.528 | 14.8 | 25.6 | 3744 | 6918 | 4a | - | - | 29.8 | Ablation zone | - | Crevasses, potholes, deformed and looped moraines. | Present study |
| 10 | G074599E36569N | Sarbzea | Tributary | Karakoram | 36.569 | 74.599 | 8.4 | 15.1 | 3402 | 6387 | 3a | 9 | 1989-1998 | 15.1 | Ablation zone | - | ~1.1 km advance in glacier terminus during 1990-2000,crevasses, shear margin and ogives. | Present study |
| 11 | G074615E36526N | Batura First | Tributary | Karakoram | 36.526 | 74.615 | 28.6 | 272.1 | 2614 | 7778 | 3a | - | - | 21.5 | Ablation zone | - | High surface velocity, crevasses, ogives and looped moraines. | Present study |
| 12 | G074758E36474N | Pasu | Main | Karakoram | 36.474 | 74.758 | 26.7 | 62.6 | 2571 | 7569 | 1c | - | - | 2.9 | Moraine | - | Rapid advance of 1200 m between 1907 and 1913. ogives and crevasses. | 24, 35, 52 |
| 13 | G074761E36429N | Ghulkin | Main | Karakoram | 36.429 | 74.761 | 17.4 | 30.8 | 2497 | 7294 | 6 | - | - | 21.5 | Ablation zone | - | Advanced between 1913–25 and 1966–78 (Ref. 52). Ogives, potholes and heavily crevasses. During 1990-2013 Ghulkin Glacier did not show much variation in surface flow. | 24, 47, 52, Modified by present study |
| 14 | G074240E36509N | East Kukuar | Main | Karakoram | 36.509 | 74.240 | 19.2 | 123.3 | 3005 | 6902 | 1c | - | - | 12.2 | Ablation zone | - | Rapid advance of ~10 km by 1915 to join Baltar Glacier, compared to retreated position in 1830s (Ref. 9). Retreat of ~2.7±0.12 km between 1979 and 2014 (Present study). | 9, 24, 26, 41, 49, Modified by present study |
| 15 | G074361E36517N | Toltar | Tributary | Karakoram | 36.517 | 74.361 | 12.0 | 18.7 | 3265 | 6891 | 1c | - | - | 41.6 | Ablation zone | - | Potholes and heavily crevasses. | 24 |
| 16 | G074418E36459N | Baltar | Main | Karakoram | 36.459 | 74.418 | 19.5 | 71.9 | 3015 | 7707 | 1a | - | - | 21.0 | Ablation zone | - | Rapid advance of ~10 km by 1915 to join Kukuar Glacier (Ref.49), compared to retreated position in 1830s. Retreat of ~8 km between 1930s and today (Ref. 9). | 1, 9, 39, 41, 49 |
| 17 | G074499E36397N | Muchuhar | Main | Karakoram | 36.397 | 74.499 | 18.2 | 87.6 | 2917 | 7752 | 4c | - | - | 14.8 | Ablation zone | - | Advanced between 1970 and 1976? and ~5.1±0.12 km retreat in glacier terminus from 1979 to 2014. | Present study |
| 18 | G074614E36396N | Hassanabad | Main | Karakoram | 36.396 | 74.614 | 17.8 | 51.8 | 2500 | 7586 | 1a | - | - | 12.7 | Ablation zone | 315±57 | Advanced 9.7 km within 2.5 months in 1904-1905 (Ref. 20). Had broken into two tributaries Shispare and Muchuhar by 1954 due to 7 km retreat (Ref. 41). Again meet both tributaries before 1972 due to advance of Muchuhar (Present study). Shispare surged during 1972-76 and again from 1993 to 2002 (present study). However, Muchuhar could not meet with Shispare due to retreat of ~4 km. | 1, 9, 20, 23, 25, 27, 29, 38, 40, 41, 58, Modified by present study |
| 19 | G074166E36251N | Unnamed | Main | Karakoram | 36.251 | 74.166 | 3.2 | 2.0 | 4271 | 5535 | 6 | 5 | 1993-1998 | 0.0 | Clean ice | 383±57 | ~0.4 km advance in glacier terminus during 1989-2000 and ~0.4 km retreat between 2001 and 2016 and heavily crevasses. | Present study |
| 20 | G074565E36192N | Minapin | Main | Karakoram | 36.192 | 74.565 | 17.3 | 56.5 | 2539 | 7228 | 1c | - | - | 10.4 | Ablation zone | - | Ogives, crevasses, shear margin and 1.3 km advance during 1892-1893. | 1, 9, 24, 25, 35, 36, 38 |
| 21 | G074759E36179N | Bualtar | Main | Karakoram | 36.179 | 74.759 | 23.0 | 68.8 | 2280 | 7245 | 2a | ~15 | 1989-1990-2000? | 6.6 | Ablation zone | - | Surge began 1986-1987 after landslides onto glacier (Ref.16), ~2 km advance 1989-1990 (Ref. 25), ~5 km advance 1990-2000 (possibly continuation of 1989-1990 surge?), crevasses, ogives and deformed moraine. | 1, 9, 16, 24, 25, 34 |
| 22 | G074845E36152N | Barpu | Main | Karakoram | 36.152 | 74.845 | 24.2 | 51.3 | 2831 | 6809 | 4a | - | - | 10.9 | Ablation zone | - | Ogives, potholes and heavily crevasses. | 8, 24 |
| 23 | G074892E36124N | Sumaiyar Bur | Tributary | Karakoram | 36.124 | 74.892 | 19.0 | 54.2 | 3167 | 7304 | 1c | - | - | 22.7 | Ablation zone | - | Heavily crevasses, ogives, shear margin and deformed moraines. Advanced ~9 km to join Bualtar in late 1800s. Sudden, massive thickening reported in 1992. | 1, 8, 9, 25 |
| 24 | G074979E36128N | Yengutz Har | Main | Karakoram | 36.128 | 74.979 | 8.0 | 9.0 | 3503 | 6235 | 1a | - | - | 35.8 | Ablation zone | - | Report of 2 mile advance in 8 days between 1902 and 1903, 2.6 km advance in spring. | 1, 9, 23, 24, 25, 27, 29, 34, 38 |
| 25 | G075012E36069N | Garumbar | Main | Karakoram | 36.069 | 75.012 | 9.1 | 18.2 | 3845 | 6624 | 1c | - | - | 40.5 | Ablation zone | - | Report of 2.5 km advance sometime between 1892 and 1925. Potholes and heavily crevasses. | 1, 9, 23, 24, 34, 38 |
| 26 | G074894E35888N | Mani | Main | Karakoram | 35.888 | 74.894 | 16.0 | 37.3 | 2694 | 7329 | 4a | - | - | 22.0 | Ablation zone | - | Crevasses and ogives. | 1, 9 |
| 27 | G075081E35819N | Kuliah (Goropha) | Main | Karakoram | 35.819 | 75.081 | 18.6 | 51.3 | 2851 | 7310 | 4a | - | - | 12.6 | Ablation zone | - | High surface velocity, heavily crevasses, shear margin and ogives. | Present study |
| 28 | G075010E35846N | East Mani | Tributary | Karakoram | 35.846 | 75.010 | 8.7 | 12.7 | 3595 | 6598 | 3aB | ~4 | Sometime between 1972-1977 and 1990-1994 | 37.0 | Ablation zone | - | High surface velocity, potholes, crevasses and ogives. | Present study |
| 29 | G074989E35805N | Kutiah (Khotia Lungma) | Main | Karakoram | 35.805 | 74.989 | 9.4 | 18.2 | 3597 | 5987 | 1a | - | - | 22.0 | Ablation zone | - | Rapid 12 km advance in 3 months during March-May 1953. Potholes, crevasses and ogives. | 9, 10, 11, 23, 24, 25, 29, 38 |
| 30 | G075099E35888N | West Marpoh | Tributary | Karakoram | 35.888 | 75.099 | 12.4 | 20.5 | 3619 | 6188 | 3a | 4 | 1994-1998 | 12.9 | Ablation zone | - | High surface velocity during 1994-1998, crevasses and ogives. | 24 |
| 31 | G075272E35771N | Niamur Gans | Main | Karakoram | 35.771 | 75.272 | 5.5 | 7.9 | 4078 | 5451 | 1c | - | - | 7.8 | Moraine | - | Rapid advance of several miles in 1902-1903. Potholes and crevasses. | 9, 23, 25, 57 |
| 32 | G075111E35954N | Chogo Lungma | Main | Karakoram | 35.954 | 75.111 | 44.7 | 275.0 | 2757 | 7168 | 4a | - | - | 20.0 | Ablation zone | - | High surface velocity, crevasses, potholes, and shear margin. | Present study |
| 33 | G075153E36073N | East Makrong | Tributary | Karakoram | 36.073 | 75.153 | 9.5 | 16.6 | 3777 | 5992 | 4b | - | - | 39.5 | Ablation zone | - | Terminus advance ~0.6 km between 1990 and 2000 (Ref. 9). | 9 |
| 34 | G075323E36076N | Hispar | Main | Karakoram | 36.076 | 75.323 | 50.0 | 162.1 | 3106 | 7306 | - | - | - | 27.6 | Ablation zone | - | Extensive looped and folded moraines in upper ablation area. | 1, 9 |
| 35 | G075384E36067N | Khani Basa | Tributary of Hispar | Karakoram | 36.067 | 75.384 | 25.0 | 69.9 | 4208 | 7730 | 3aB | - | - | 18.2 | Ablation zone | - | Extensive crevasses and potholes , deformed moraines in upper ablation area. Surge started between 2014 and 2015 and continue. | Present study |
| 36 | G075312E36140N | Yutmaru Glacier | Tributary of Hispar | Karakoram | 36.140 | 75.312 | 34.3 | 124.0 | 3678 | 6226 | 3aB | - | - | 33.9 | Ablation zone | - | Extensive crevasses and potholes , deformed moraines in upper ablation area. Surge started between 2014 and 2015 and continue. | Present study |
| 37 | G075209E36140N | Pumari Chhissh | Tributary of Hispar | Karakoram | 36.140 | 75.209 | 7.9 | 14.4 | 3971 | 7703 | 3aA | 2 | 1988-1990 | 18.5 | Ablation zone | - | Surge in late 1800s?, looped moraines, shear margin and crevasses and rapid ~1 km advance and 20 m thickening between 1988-1989 and continued till 1990 (Present study). | 1, 8, 9, 24, 25, 55 |
| 38 | G075127E36215N | Kunyang | Tributary of Hispar | Karakoram | 36.215 | 75.127 | 26.0 | 111.3 | 3475 | 7850 | 3aB | 3 | 1972-1973; 2006-2009 | 19.5 | Ablation zone | - | High surface velocity, advance in glacier terminus during 1972-1973 and 2006-2009, looped moraines, and intense crevasses. | 24, 45 |
| 39 | G074965E36249N | Gharesa (Trivor) | Main | Karakoram | 36.249 | 74.965 | 23.0 | 80.3 | 3301 | 7591 | 1b | 12 | 1998-2011 | 12.0 | Ablation zone | 2944±57 | High surface velocity during 1998-2011, intense crevasses and deformed moraines. | 9 |
| 40 | G074965E36329N | Balt Bare | Main | Karakoram | 36.329 | 74.965 | 11.0 | 15.6 | 3170 | 7111 | 1b | 6 | 1976-1979 and probably continue; 2006-2012; | 10.9 | Ablation zone | - | High surface velocity, ~1.0 km advance in glacier terminus during 2006-2012 and crevasses. | 9, 24, 25, 56, 61 |
| 41 | G075074E36416N | Momhil | Main | Karakoram | 36.416 | 75.074 | 29.0 | 73.7 | 2906 | 7661 | 2b | 1 | 1977-1978 | 16.5 | Ablation zone | - | High surface velocity during 1977-1978, crevasses, ice pinnacles and deformed moraines. | Present study |
| 42 | G075358E36355N | Yazghil | Main | Karakoram | 36.355 | 75.358 | 28.0 | 114.9 | 3242 | 7810 | 5 | 1 | 1990, 1998 and 2006? | 4.6 | Moraine | 186±57 | Front advanced in 1990, 1998 and 2006 (3 times), high surface velocity, ogives, potholes, crevasses, looped moraine and shear margin. | Present study |
| 43 | G075327E36297N | Skirish | Tributary | Karakoram | 36.297 | 75.327 | 9.4 | 14.6 | 4066 | 7389 | 3a | 7 | 1993-2000 | 16.8 | Ablation zone | - | High surface velocity during 1993-2000, crevasses, deformed moraines and potholes. | Present study |
| 44 | G075349E36289N | Unnamed tributary of Skirish | Tributary | Karakoram | 36.289 | 75.349 | 4.7 | 3.6 | 4288 | 6466 | 3aB | 7 | 2006-2013 | 6.5 | Moraine | 954±57 | ~1.0 km advance in glacier terminus during 2000-2014, high surface velocity, crevasses and thickening of terminus area. | Present study |
| 45 | G075362E36305N | Unnamed | Main | Karakoram | 36.305 | 75.362 | 4.0 | 2.0 | 4511 | 6463 | 6 | 5 | 2005-2010 | 2.4 | Moraine | 226±57 | ~0.3 km advance in glacier terminus during 2005-2010 and crevasses. | Present study |
| 46 | G075419E36281N | Yukshin Gardan | Tributary | Karakoram | 36.281 | 75.419 | 15.5 | 41.4 | 3484 | 7733 | 4b | - | - | 18.3 | Ablation zone | - | High surface velocity, crevasses, ogives, potholes and shear margin. | Present study |
| 47 | G075475E36288N | Khurdopin | Main | Karakoram | 36.288 | 75.475 | 38.0 | 163.0 | 3311 | 7707 | 2a | 1 | 1978-1979, 1998-1999 (peak) | 16.0 | Ablation zone | - | High surface velocity during 1978-1979, 1998-1999 (peak), crevasses, ice pinnacles, shear margin, extensive looped and folded moraines. | 9, 24, 26, 28, 43 |
| 48 | G075530E36231N | Unnamed | Main | Karakoram | 36.231 | 75.530 | 6.8 | 6.5 | 4331 | 6370 | 6 | 7 | 2006-2013 | 18.3 | Ablation zone | 516±57 | ~0.5 km advance in glacier terminus during 2004-2014, potholes and crevasses. | Present study |
| 49 | G075551E36205N | Unnamed | Main | Karakoram | 36.205 | 75.551 | 7.4 | 9.8 | 4556 | 6391 | 1b | 7 | 2006-2013 | 7.4 | Moraine | 675±57 | High surface velocity, ~0.7 km advance in glacier terminus, potholes and crevasses. | Present study |
| 50 | G075024E36592N | Murkhun | Main | Karakoram | 36.592 | 75.024 | 6.2 | 6.1 | 4579 | 6190 | 6 | - | - | 6.3 | Moraine | 485±57 | ~0.5 km advance in glacier terminus during 1993-2014, crevasses, looped shaped terminus and thickening of terminus area. | 24 |
| 51 | G075033E36648N | North Karun Koh | Main | Karakoram | 36.648 | 75.033 | 8.5 | 14.0 | 4235 | 6706 | 2c | 10 | 1996-2006 | 19.8 | Ablation zone | 594±57 | About 0.6 km advance in glacier terminus during 1998-2006, thickening of ablation area and crevasses. | Present study |
| 52 | G075218E36752N | Unnamed | Main | Karakoram | 36.752 | 75.218 | 6.7 | 9.8 | 4798 | 6132 | 1b | 6 | 1990-1996 and 2015 and continue | 0.0 | Clean ice | - | A large amount of ice mass transferred from the reservoir zone to receiving zone during 1990-1996 and again in 2015 and continue. | Present study |
| 53 | G075230E36735N | Unnamed | Main | Karakoram | 36.735 | 75.230 | 7.0 | 9.3 | 4646 | 6096 | 1b | 7 | 1999-2006 | 5.9 | Moraine | 894±57 | ~0.9 km advance in glacier terminus during 2003-2006, crevasses and looped moraines. | 46 |
| 54 | G075253E36721N | Unnamed | Main | Karakoram | 36.721 | 75.253 | 4.6 | 4.4 | 4680 | 5859 | 2c | 18 | 1996-2014 | 3.7 | Moraine | 723±57 | ~0.7 km slow advance in glacier terminus during 1996-2014 and advancement is still continue in 2016 satellite image, crevasses and potholes. | Present study |
| 55 | G075272E36714N | Unnamed | Main | Karakoram | 36.714 | 75.272 | 4.9 | 5.6 | 4686 | 5918 | 6 | - | - | 6.0 | Moraine | 262±57 | ~0.3 km advance in glacier terminus during 2004-2014 and crevasses. | Present study |
| 56 | G075400E36668N | Unnamed | Main | Karakoram | 36.668 | 75.400 | 4.5 | 2.8 | 4703 | 5904 | 2c | 12 | 2002-2014 and probably continue | 0.0 | Clean ice | 791±57 | ~0.7 km advance in glacier terminus during 1994-2014, potholes and crevasses. | Present study |
| 57 | G075431E36737N | Shop Dur | Main | Karakoram | 36.737 | 75.431 | 6.4 | 10.8 | 4697 | 6050 | 4c | - | - | 7.1 | Ablation zone | - | Continuous retreat of ~1.2 km from 1979 to 2014, crevasses and potholes. | Present study |
| 58 | G075457E36749N | Unnamed | Main | Karakoram | 36.749 | 75.457 | 6.7 | 6.4 | 4707 | 5956 | 1b | 4 | 1973-1977 | 5.5 | Moraine | - | ~1.0 km advance in glacier terminus during 1973-1977, crevasses and potholes. | Present study |
| 59 | G075557E36739N | Unnamed | Main | Karakoram | 36.739 | 75.557 | 7.2 | 14.4 | 4691 | 6072 | 1b | 3 | 1999-2002 | 2.5 | Moraine | 958±57 | High surface velocity, ~1.0 km advance in glacier terminus during 1999-2002, crevasses, potholes and deformed moraines. | Present study |
| 60 | G075568E36695N | Unnamed | Main | Karakoram | 36.695 | 75.568 | 5.0 | 10.1 | 4905 | 6034 | 4c | - | - | 7.8 | Ablation zone | - | Continuous retreat of ~0.7 km from 1979 to 2014, crevasses, potholes and deformed moraines. | Present study |
| 61 | G075538E36656N | Unnamed | Main | Karakoram | 36.656 | 75.538 | 6.0 | 13.3 | 4732 | 6065 | 1b | 5 | 2009-2014 and probably continue | 1.3 | Moraine | - | High surface velocity, lobe-shaped terminus, crevasses, potholes and deformed moraines. | Present study |
| 62 | G075514E36554N | Unnamed | Main | Karakoram | 36.554 | 75.514 | 7.4 | 9.5 | 4537 | 5986 | 1b | ~20 | 1990-2014 | 0.4 | Moraine | 1906±57 | High surface velocity, ~2.0 km advance in glacier terminus during 1990-2014, crevasses, potholes and deformed moraines. | Present study |
| 63 | G075733E36454N | Unnamed tributary of Shuwert-Yaz | Tributary | Karakoram | 36.454 | 75.733 | 4.7 | 4.1 | 4855 | 6102 | 4b | - | - | 2.4 | Moraine | - | ~0.3 km advance in glacier terminus during 2006-2013 when surrounding glaciers receding, potholes and intense crevasses. | Present study |
| 64 | G075691E36303N | Unnamed | Main | Karakoram | 36.303 | 75.691 | 3.1 | 2.8 | 4774 | 5937 | 6 | 5 | 1996-2001 | 2.0 | Moraine | 258±57 | About 0.3 km slow advance in glacier terminus during 1998-2011, deformed moraines and crevasses. | Present study |
| 65 | G075660E36264N | Virjerab | Main | Karakoram | 36.264 | 75.660 | 38.0 | 168.2 | 3585 | 6479 | 4a | - | - | 13.9 | Ablation zone | - | Intense crevasses, potholes, ice pinnacles, shear margin, extensive looped and folded moraines. | 9, 25 |
| 66 | G075776E36211N | Skorga | Main | Karakoram | 36.211 | 75.776 | 14.1 | 36.7 | 4372 | 6300 | 1b | 9 | 1993-2002 | 4.3 | Moraine | 1140±57 | Terminus retreat of ~1.4±0.12 km during 1979-1990 and terminus advance (by~1.0±0.06 km) in 1999-2006, high surface velocity, intense crevasses and potholes. | 9 |
| 67 | G075822E36140N | Unnamed | Tributary of Braldu | Karakoram | 36.140 | 75.822 | 4.3 | 4.4 | 4660 | 6045 | 4c | - | - | 0.0 | Clean ice | - | Fan shaped terminus in 1977 and retreat of ~0.5±0.12 km during 1977-1990 and retreat of ~0.8±0.06 km in 1990-2014. | Present study |
| 68 | G075836E36121N | Unnamed | Tributary of Braldu | Karakoram | 36.121 | 75.836 | 5.2 | 5.7 | 4473 | 5932 | 3aB | - | - | 6.8 | Moraine | - | Fan shaped terminus in 1977 and retreat of ~0.4±0.12 km during 1977-1990 and advance of ~0.6 km±0.06 in 2002-2013, crevasses and deformed moraines. | Present study |
| 69 | G075862E36098N | Braldu | Main | Karakoram | 36.098 | 75.862 | 32.2 | 162.9 | 3979 | 6528 | 2b | - | - | 12.3 | Ablation zone | - | Surge begin during ~2013 to 2016 and possibly continue. Intense crevasses, shear margin, potholes, deformed moraines. | 1, 9, 24, 25, 52 |
| 70 | G075916E36094N | East lll Braldu | Tributary of Braldu | Karakoram | 36.094 | 75.916 | 6.7 | 8.0 | 4676 | 6324 | 3aB | 7 | 1999-2006 | 0.0 | Clean ice | 1076±57 | Terminus advance of ~1.1±0.06 km during 2001-2010, high surface velocity, crevasses and potholes. | Present study |
| 71 | G075907E36200N | Unnamed | Main | Karakoram | 36.200 | 75.907 | 10.5 | 23.1 | 4481 | 6516 | 1b | 12 | 1999-2011 | 8.0 | Ablation zone | 856±57 | Front advance of ~0.9 km during 2005-2014, high surface velocity, intense crevasses, deformed moraines and potholes. | Present study |
| 72 | G075958E36215N | Wosmi Yaz | Main | Karakoram | 36.215 | 75.958 | 8.7 | 20.8 | 4477 | 6271 | 4a | - | - | 5.7 | Ablation zone | - | Crevasses, shear margin, deformed moraines, ice pinnacles and potholes. | Present study |
| 73 | G075985E36269N | Shahim-Dur (Saxinitulu) | Main | Karakoram | 36.269 | 75.985 | 16.5 | 41.0 | 4561 | 6511 | 1b | 8 | 2006-2014 | 5.4 | Moraine | 1204±57 | High surface velocity, ~1.2 km advance in glacier terminus during 2010-2014, crevasses and deformed moraines. | 46 |
| 74 | G076075E36248N | Unnamed | Main | Karakoram | 36.248 | 76.075 | 7.6 | 13.1 | 4738 | 6182 | 6 | 9 | 2007-2016 | 3.5 | Moraine | 602±57 | Terminus advance of ~0.6 km during 2007-2016, high surface velocity and crevasses. | Present study |
| 75 | G076163E36182N | Unnamed tributary of North Crown | Tributary | Karakoram | 36.182 | 76.163 | 9.8 | 22.8 | 4563 | 6460 | 3aB | 8 | 2005-2013 | 0.0 | Clean ice | 1025±57 | Terminus advance of ~3.2±0.12 km from 1978 to 1990, advanced again ~1.0±0.06 km during 2005-2013, high surface velocity and crevasses. | 9, 27, Modified by present study |
| 76 | G076201E36168N | North Crown | Main | Karakoram | 36.168 | 76.201 | 16.3 | 34.5 | 4261 | 6890 | 1b | 4 | 2008-2012 | 12.1 | Moraine | 1140±57 | Terminus advance of ~1.0 km from 2008 to 2012, high surface velocity, and crevasses. | 24, 46, 52 |
| 77 | G076306E36126N | Unnamed | Main | Karakoram | 36.126 | 76.306 | 7.9 | 14.8 | 4612 | 6478 | 1b | 9 | 1997-2006 | 10.5 | Ablation zone | 1254±57 | Terminus advance of ~1.3 km from 1997 to 2006, high surface velocity and crevasses. | 24, 46 |
| 78 | G076317E36082N | Unnamed | Main | Karakoram | 36.082 | 76.317 | 9.8 | 10.9 | 4645 | 6538 | 1b | 14 | 2000-2014 | 0.0 | Clean ice | 1428±57 | Terminus advance of ~1.4 km during 2000-2014, high surface velocity, potholes and intense crevasses. | 24, 46 |
| 79 | G076226E36081N | Unnamed | Main | Karakoram | 36.081 | 76.226 | 5.7 | 11.8 | 4447 | 7200 | 1b | 4 | 2010-2014 | 3.5 | Ablation zone | 1085±57 | Terminus advance of ~1.1 km during 2010-2014, high surface velocity and looped moraine. | 46 |
| 80 | G076153E36114N | North Skamri | Tributary | Karakoram | 36.114 | 76.153 | 21.9 | 65.8 | 4582 | 7202 | 4b | - | - | 9.9 | Moraine | - | Intense crevasses, potholes and deformed moraines. | 1, 9 |
| 81 | G076124E36111N | Unnamed tributary of North Skamri | Tributary | Karakoram | 36.111 | 76.124 | 3.3 | 2.3 | 4914 | 5889 | 3a | - | - | 0.0 | Clean ice | - | Terminus advance of ~1 km during 1990-1998 due to pushed by advanced trunk glacier and looped terminal moraine. | 9, Modified by present study |
| 82 | G076117E36118N | Unnamed tributary of North Skamri | Tributary | Karakoram | 36.118 | 76.117 | 4.3 | 4.3 | 4952 | 6260 | 3a | - | - | 0.0 | Clean ice | - | Terminus advance of ~1 km during 1990-1998 due to pushed by advanced trunk glacier and looped terminal moraine. | 9, Modified by present study |
| 83 | G076085E36143N | Unnamed tributary of North Skamri | Tributary | Karakoram | 36.143 | 76.085 | 14.4 | 19.5 | 4967 | 6401 | 3a | 4 | 1990-1994 | 5.1 | Moraine | - | Terminus advance of ~1.3 km during 1990-1994, high surface velocity and looped moraine. | 9, Modified by present study |
| 84 | G076071E36102N | Unnamed tributary of Skamri | Tributary | Karakoram | 36.102 | 76.071 | 16.6 | 76.6 | 4048 | 6502 | 3a | 3 | 1993-1996 | 29.9 | Ablation zone | - | Folded and distorted moraines, front advanced from 1990 to 2000, crevasses, shear margin and looped moraine. | 9, Modified by present study |
| 85 | G076034E36102N | Unnamed | Tributary | Karakoram | 36.102 | 76.034 | 6.7 | 6.3 | 4886 | 6054 | 3a | 3 | 1993-1996 | 0.0 | Clean ice | - | Terminus advance ~1.4 km into tributary during 1993-1996, potholes and distortion of moraines. | 9, Modified by present study |
| 86 | G076167E36061N | Skamri | Main | Karakoram | 36.061 | 76.167 | 28.5 | 108.9 | 4426 | 6538 | 2b | 4 | 1990-1994 | 3.8 | Ablation zone | - | Surge between 1990 and 1994. Extensive folded moraines, crevasses, shear margin, ice pinnacle and potholes. | 9, 23, 24, Modified by present study |
| 87 | G076193E36038N | South Skamri | Tributary | Karakoram | 36.038 | 76.193 | 17.5 | 105.4 | 4374 | 7019 | 3a | - | - | 3.5 | Moraine | - | Terminus advance during 1978-1990 (by ~0.7±0.12 km) and 1990-2013 (by ~1.5±0.06 km), looped and distorted terminal moraine. | 9, Modified by present study |
| 88 | G076042E35985N | Little Skamri | Tributary | Karakoram | 35.985 | 76.042 | 9.1 | 13.3 | 4321 | 6544 | 3a | 4 | 1990-1994 | 18.6 | Ablation zone | - | Rapid terminus advance (4.8±0.12 km in late 1970s) observed in satellite images, terminus advance ~0.5 km 1990-1994, increase in surface crevassing and lobe-shaped terminus (Ref.9). | 1, 9, 25 |
| 89 | G076057E35973N | Drenmang | Tributary | Karakoram | 35.973 | 76.057 | 12.1 | 19.9 | 4271 | 6544 | 3aB | 2 | 2005-2007 | 8.5 | Ablation zone | - | Previous surge in 1930-1931 (and adjacent Little Skamri in 1977-1978) (H98). Rapid terminus advance during 2005-2007, looped moraines and heavily crevassed surface. | 1, 9, 24, 25, 27, 51 |
| 90 | G076107E35933N | Unnamed tributary of Chiring | Tributary | Karakoram | 35.933 | 76.107 | 5.5 | 8.0 | 4743 | 6556 | 3a | - | - | 9.9 | Ablation zone | - | Terminus advance of ~0.6 km between 1991 and 2013, potholes and lobe-shaped terminus. | Present study |
| 91 | G076018E35955N | Nobande Sobonde (Panmah) | Tributary | Karakoram | 35.955 | 76.018 | 31.5 | 117.9 | 3941 | 6351 | - | - | - | 13.4 | Ablation zone | - | Extensive deformed and looped moraines in ablation zone and potholes. | 1, 9 |
| 92 | G076062E35921N | Chiring | Tributary | Karakoram | 35.921 | 76.062 | 15.2 | 53.7 | 4265 | 6805 | 3a | 5 | 1990-1995 | 4.5 | Ablation zone | - | Front advance of ~2.0 km during 1990-1995, looped and folded moraines, potholes and intense crevasses. | 1, 9, 20, 24, 25, 27, 59, 60 |
| 93 | G076077E35906N | Nera | Tributary | Karakoram | 35.906 | 76.077 | 3.7 | 4.0 | 4587 | 5976 | 4b | - | - | 0.0 | Clean ice | - | Glacier terminus advance (~0.2 km), potholes, fan-shaped terminus and intense crevasses.. | 24 |
| 94 | G076051E35900N | South Chiring (Maedan) | Tributary | Karakoram | 35.900 | 76.051 | 10.6 | 14.9 | 4188 | 6036 | 2c | ~15 | 1990-2005 | 5.3 | Moraine | - | Front advance of ~2.3 km during 1990-2006, looped and folded moraines and potholes and intense crevasses. | 1, 9, 24, 18, 20, 25, 48, Modified by present study |
| 95 | G076039E35882N | Second Feriole (Shingchukpi) | Tributary | Karakoram | 35.882 | 76.039 | 10.5 | 15.1 | 4090 | 6035 | 2c | 12 | 1993-2005 | 3.3 | Moraine | 3381±57 | Rapid terminus advance ~3.3 km during 1993-2005, increase in surface crevassing, high surface velocity and lobed-shaped terminus. | 1, 9, 24, 27, Modified by present study |
| 96 | G076016E35831N | First Feriole | Main | Karakoram | 35.831 | 76.016 | 10.0 | 14.1 | 4011 | 6208 | 2c | 12 | Surge before 1972 probably in last of 1960s & retreat1977-1990 & surge 2002-2014 | 0.0 | Clean ice | 2405±57 | Glacier in forward (surged) position in 1977, rapid retreat of 2.8±0.12 km during 1977-1990 when nearby glaciers advancing (Ref.9), rapid terminus advance ~2.4±0.06 km during 2002-2014 (Present study). | 9, 24, Modified by present study |
| 97 | G075961E35932N | Unnamed | Main | Karakoram | 35.932 | 75.961 | 4.4 | 7.1 | 4468 | 5676 | 4c | 9 | 2000-2009 | 0.0 | Clean ice | 276±57 | ~0.8 km retreated during 1977-1990 and ~0.3 km slow advance in glacier terminus during 2001-2011 and intense crevasses. | Present study |
| 98 | G075910E35955N | Unnamed tributary of Choktoi | Main | Karakoram | 35.955 | 75.910 | 3.8 | 3.1 | 4502 | 5839 | 6 | 15 | 1998-2013 | 7.1 | Moraine | - | ~0.5 km slow advance in glacier terminus during 1998-2013, crevasses and elevation loss in accumulation zone and ice gain in ablation zone based on 2006 and 2015 Google earth satellite images. | Present study |
| 99 | G075747E35893N | Uzun Brakk | Tributary | Karakoram | 35.893 | 75.747 | 11.1 | 31.4 | 4097 | 7057 | 4b | - | - | 8.1 | Ablation zone | - | Advance of ~1 km between 1990 and 2001 into Biafo Glacier. | 9 |
| 100 | G075486E35872N | Darung | Main | Karakoram | 35.872 | 75.486 | 2.4 | 2.7 | 4561 | 5463 | 1b | 8 | Before 1977; 1990-1998 | 3.1 | Moraine | 935±57 | ~1.0 km retreated during 1977-1990. ~1.0 km sudden advance in glacier terminus during 1990-1999, potholes and crevasses. | Present study |
| 101 | G075656E35824N | Tonga | Main | Karakoram | 35.824 | 75.656 | 6.6 | 11.1 | 4129 | 5870 | 1b | 4 | 1990-1994; 2014-2016 and possibly continue | 5.1 | Moraine | 235±57 | High surface velocity, crevasses and increase of terminus thickness and advance. | 9, Modified by present study |
| 102 | G075790E35749N | Unnamed | Main | Karakoram | 35.749 | 75.790 | 3.3 | 5.6 | 4398 | 5712 | 6 | 4 | 1994-1998 | 5.9 | Moraine | 1595±57 | ~1.5 km sudden advance in glacier terminus during 1990-1998 and crevasses. | Present study |
| 103 | G075792E35889N | Baintha Lukpar | Tributary | Karakoram | 35.889 | 75.792 | 11.9 | 25.9 | 4073 | 6906 | 4b | - | - | 14.4 | Ablation zone | - | High surface velocity during 1990-1999, increase in surface crevassing and ice thickening in ablation zone. | Present study |
| 104 | G075817E35863N | Unnamed | Main | Karakoram | 35.863 | 75.817 | 2.7 | 2.6 | 4522 | 5761 | 6 | 4 | 1994-1998 | 6.6 | Ablation zone | - | Terminus advance of ~0.3 km between 1991 and 1999, and ice thickening in ablation zone, potholes and crevasses. | Present study |
| 105 | G075913E35836N | Unnamed tributary of Dumulter Gans | Tributary | Karakoram | 35.836 | 75.913 | 5.7 | 36.3 | 3773 | 6354 | 3a | - | - | 7.9 | Ablation zone | - | Terminus advance of ~1 km into tributary between 1990 and 2000. | 9 |
| 106 | G075912E35783N | Tsa Gans | Main | Karakoram | 35.783 | 75.912 | 5.8 | 8.2 | 3954 | 5837 | 6 | - | - | 9.7 | Ablation zone | 435±57 | ~0.5 km advance in glacier terminus during 1996-2005, potholes and crevasses. | Present study |
| 107 | G075717E35632N | Pakore | Main | Karakoram | 35.632 | 75.717 | 6.0 | 11.5 | 4019 | 5791 | 1b | 5 | 1993-1998 | 7.1 | Moraine | 1926±57 | Advance of ~2.0 km between 1990 and 2000, high surface velocity and crevasses. | Present study |
| 108 | G075782E35614N | Nang Brok | Main | Karakoram | 35.614 | 75.782 | 7.2 | 11.5 | 3678 | 5690 | 2b | - | - | 20.2 | Ablation zone | - | Upper part of glacier advance ~0.6 km during 1990-2000 but could not reach at terminus and increase in surface crevassing. | 9, Modified by present study |
| 109 | G075991E35613N | Unnamed | Main | Karakoram | 35.613 | 75.991 | 2.0 | 0.4 | 4092 | 4920 | 6 | 5 | 1991-1996 | 68.4 | Ablation zone | 362±57 | ~0.4 km advance in glacier terminus during 1990-1998, ice thickening in ablation zone and crevasses. | Present study |
| 110 | G075983E35571N | Unnamed | Main | Karakoram | 35.571 | 75.983 | 2.2 | 0.9 | 4535 | 5679 | 6 | 3 | 1993-1996 | 0.0 | Clean ice | 504±57 | ~0.5 km advance in glacier terminus during 1990-1999, ice thickening in ablation zone and crevasses. | Present study |
| 111 | G075990E35548N | Unnamed | Tributary | Karakoram | 35.548 | 75.990 | 3.5 | 1.6 | 4141 | 5726 | 6 | - | 1993-2000 | 17.1 | Moraine | 532±57 | Terminus advance of ~0.6 km during 1993-2000 and bulbous terminus. | 9 |
| 112 | G076003E35520N | West Ching Kang | Main | Karakoram | 35.520 | 76.003 | 10.0 | 30.4 | 3932 | 5887 | 1b | 6 | 1990-1996 | 11.9 | Ablation zone | 536±57 | Advance of white ice areas by up to 2 km 1990-2000 and folded moraines across terminus. | 9, Modified by present study |
| 113 | G076071E35514N | Unnamed | Main | Karakoram | 35.514 | 76.071 | 4.5 | 3.4 | 4203 | 5918 | 1b | 4 | 1990-1994 | 27.3 | Ablation zone | 1826±57 | ~1.8 km advance in glacier terminus, and crevasses. | Present study |
| 114 | G076091E35509N | Unnamed | Main | Karakoram | 35.509 | 76.091 | 2.1 | 1.0 | 4368 | 5617 | 6 | 5 | 1991-1996 | 13.0 | Ablation zone | - | ~0.2 km sudden advance in glacier terminus (1991-1996) and intense crevasses. | Present study |
| 115 | G076146E35564N | Unnamed | Main | Karakoram | 35.564 | 76.146 | 7.6 | 8.2 | 4611 | 6449 | 1b | - | Sometime between 2000 and 2013 | 9.9 | Ablation zone | 624±57 | ~0.6 km advance in glacier terminus during 2003-2013 and intense crevasses. | Present study |
| 116 | G076115E35615N | Unnamed | Main | Karakoram | 35.615 | 76.115 | 3.0 | 3.3 | 4614 | 5759 | 1b | 9 | 1991-2000 | 5.7 | Moraine | 1177±57 | ~1.2 km advance in glacier terminus during 1991-2001 and intense crevasses. | Present study |
| 117 | G076167E35624N | Unnamed | Main | Karakoram | 35.624 | 76.167 | 4.8 | 5.0 | 4255 | 6327 | 6 | - | Sometime between 2006 and 2014 | 10.8 | Moraine | 465±57 | ~0.5 km advance in glacier terminus sometime between 2006 and 2014, intense crevasses and bulbous terminus. | Present study |
| 118 | G076184E35653N | Unnamed | Main | Karakoram | 35.653 | 76.184 | 3.6 | 1.2 | 4056 | 6190 | 6 | 5 | 1991-1996 | 36.2 | Ablation zone | 630±57 | ~0.6 km advance in glacier terminus during 1991-1998 and intense crevasses. | Present study |
| 119 | G076227E35642N | Liligo | Tributary | Karakoram | 35.642 | 76.227 | 15.1 | 32.7 | 3809 | 7201 | 3aB | 5 | 1991-1996 | 9.5 | Ablation zone | 1395±57 | High surface velocity, ~1.4 km advance in glacier terminus during 1991-1996, crevasses and deformed moraines. | 2, 9, 14, 24, 25, 42 |
| 120 | G076236E35502N | Tributary of Lokpar | Tributary | Karakoram | 35.487 | 76.246 | 6.0 | 56.5 | 3627 | 6452 | 6 | 4 | 1989-1993 | 22.0 | Ablation zone | - | Surge of Lokpar tributary followed by steepening and ~1.5 km advance of main terminus (Ref.9). | 9 |
| 121 | G076267E35468N | Unnamed | Main | Karakoram | 35.468 | 76.267 | 1.9 | 0.7 | 4345 | 4940 | 6 | 4 | 1993-1997 | 13.4 | Moraine | - | ~0.3 km advance in glacier terminus during 1993-1997, crevasses and ice thickening in ablation zone. | Present study |
| 122 | G076265E35535N | Unnamed | Main | Karakoram | 35.535 | 76.265 | 5.6 | 4.1 | 4174 | 6168 | 1b | 9 | 1997-2006 | 9.2 | Ablation zone | 1534±57 | High surface velocity, ~1.5 km advance in glacier terminus during 1997-2006, ice thickening in ablation zone and crevasses. | 24, Modified by present study |
| 123 | G076300E35570N | Masherbrum | Main | Karakoram | 35.570 | 76.300 | 15.7 | 33.0 | 3589 | 7740 | 2b | - | - | 26.5 | Ablation zone | - | Thickening and surge-like behavior of upper glacier, although no rapid terminus advance observed. | 9, 25 |
| 124 | G076325E35581N | Unnamed | Main | Karakoram | 35.581 | 76.325 | 2.9 | 1.7 | 4403 | 5715 | 6 | 5 | 1993-1998 | 5.6 | Moraine | - | ~0.3±0.12 km retreated between sometime 1977 and 1990. ~0.3±0.06 km advance in glacier terminus during 1993-1998, crevasses and ice thickening in ablation zone. | Present study |
| 125 | G076338E35575N | Unnamed | Main | Karakoram | 35.575 | 76.338 | 2.7 | 1.4 | 4398 | 5916 | 6 | 4 | 1993-1997 | 9.2 | Moraine | - | ~0.3 km retreated between sometime 1977 and 1990. ~0.3 km advance in glacier terminus during 1993-1997, crevasses and lobe-shaped terminus. | Present study |
| 126 | G076373E35591N | Ghondokhoro (Gondokoro) | Main | Karakoram | 35.591 | 76.373 | 20.8 | 66.4 | 3467 | 7022 | 6 | - | Between 1990-2000 | 16.8 | Ablation zone | - | Advance of white ice areas by up to 1 km 1990-2000, thickening across terminus and terminus advance of ~0.5 km. | 9 |
| 127 | G076418E35577N | Unnamed | Main | Karakoram | 35.577 | 76.418 | 4.1 | 3.9 | 4378 | 5967 | 6 | - | Sometime between 1994 and 2002 | 9.0 | Moraine | 695±57 | ~0.7 km advance in glacier terminus during 1993-2003, crevasses and ice thickening in ablation zone. | Present study |
| 128 | G076356E35788N | Lungka | Main | Karakoram | 35.788 | 76.356 | 5.4 | 4.7 | 4362 | 6110 | 1b | 8 | 1990-1998 | 11.6 | Moraine | 1292±57 | ~1.3 km advance in glacier terminus during 1990-1998 and ice thickening in ablation zone. ~0.9 km retreat between 2001 and 2015. | Present study |
| 129 | G076321E35796N | Chagaran | Tributary | Karakoram | 35.796 | 76.321 | 9.5 | 14.0 | 4160 | 6290 | 3aB | - | - | 8.4 | Ablation zone | - | Folded and looping of terminal moraines, potholes and crevasses. ~1.6 km advance in glacier terminus during 1990-2015. | 9, Modified by present study |
| 130 | G076230E35793N | Unnamed | Main | Karakoram | 35.793 | 76.230 | 2.6 | 1.7 | 4563 | 6459 | 6 | 6 | 1993-1999 | 6.6 | Moraine | - | ~0.3 km advance in glacier terminus during 1993-1999 and retreated ~0.2 km till 2013 and after that ice thickening in terminus till 2016. | Present study |
| 131 | G076112E35753N | South Hainablak (Hind Hainabrakk) | Tributary | Karakoram | 35.753 | 76.112 | 9.4 | 18.9 | 3898 | 6553 | 3a | - | - | 4.3 | Ablation zone | - | Rapid advance 1990-2000 as it merges with Trango Glacier, distorted moraines, change in surface from uncrevassed (1990) to crevassed (2000). | 9 |
| 132 | G076130E35784N | Trango | Tributary | Karakoram | 35.784 | 76.130 |  | 67.6 | 3694 | 5840 | 3aB | 5 | 2011-2016 | 12.7 | Ablation zone | - | High surface velocity, crevasses, deformed moraines and ice thickening in ablation zone. | Present study |
| 133 | G076043E35749N | North Choricho | Main | Karakoram | 35.749 | 76.043 | 7.1 | 9.7 | 4018 | 6435 | 6 | - | 1993-1999 | 13.7 | Ablation zone | 346±57 | ~0.3 km advance in glacier terminus during 1993-1999 and crevasses. | Present study |
| 134 | G076054E35762N | Unnamed tributary of North Choricho | Tributary | Karakoram | 35.762 | 76.054 | 4.4 | 2.2 | 4669 | 6147 | 6 | - | Sometime between 2004 and 2013 | 0.0 | Clean ice | 199±57 | ~0.2 km advance in glacier terminus, crevasses, ice thickening in ablation zone and fan-shaped terminus. | Present study |
| 135 | G076041E35771N | Borum | Main | Karakoram | 35.771 | 76.041 | 7.5 | 6.2 | 3816 | 6169 | 1b | 5 | 1991-1996; 2004-2009 | 0.0 | Clean ice | 1276±57 | ~1.3 km advance in glacier terminus during 1991-1996 and again advance of ~0.7 km in 2004-2009, lobe-shaped terminus and crevasses. | Present study |
| 136 | G076215E35911N | West Chang Tok | Tributary | Karakoram | 35.911 | 76.215 | 8.3 | 8.7 | 4850 | 6881 | 3aB | 6 | 1990-1996 | 7.8 | Moraine | - | High surface velocity, rapid advance of front into trunk Sarpo Laggo (by ~1.5 km) during 1990-1998, shear margin, crevasses and deformed moraines. | 9, Modified by present study |
| 137 | G076220E35935N | East Chang Tok | Tributary | Karakoram | 35.935 | 76.220 | 6.9 | 5.3 | 4805 | 6738 | 4b | - | - | 14.1 | Moraine | - | Distortion of medial moraine (Ref.9). | 9 |
| 138 | G076249E35911N | Unnamed tributary of Sarpo Laggo | Tributary | Karakoram | 35.911 | 76.249 | 6.6 | 5.5 | 4724 | 6516 | 3aB | 17 | 1990-2007 | 6.0 | Moraine | 1815±57 | Terminus advance of ~1.8 km between 1990 and 2007, crevasses and looped moraine. | 9 |
| 139 | G076263E35928N | Unnamed tributary of Sarpo Laggo | Tributary | Karakoram | 35.928 | 76.263 | 5.7 | 4.2 | 4624 | 6478 | 3aB | 3 | Sometime between 1977-1990 and 2004-2007 | 3.9 | Moraine | 1245±57 | Two rapid terminus advances of ~1.1±0.12 km each between 1977-1990 and 2004-2007, crevasses and looped moraine. | 9 |
| 140 | G076304E35885N | Moni | Tributary | Karakoram | 35.885 | 76.304 | 12.6 | 28.5 | 4488 | 6965 | 1c | - | 1980s | 8.5 | Ablation zone | - | Rapid advance of terminus into tributary Sarpo Laggo (by ~2.2±0.12 km) begin between sometime 1977 and 1990 and continue till 2000. | 9, 24, 25, Modified by present study |
| 141 | G076345E35887N | South Chongtar | Main | Karakoram | 35.887 | 76.345 | 14.0 | 41.2 | 4387 | 7337 | 4c | - | - | 8.9 | Ablation zone | - | Potholes, crevasses, deformed moraine, receded ~2.4 km between 1990 and 2014. | 9, Modified by present study |
| 142 | G076369E35933N | North Chongtar | Main | Karakoram | 35.933 | 76.369 | 6.0 | 11.4 | 4781 | 7233 | 2c | 22 | 1992-2014 | 3.2 | Moraine | 894±57 (1992-2014) | Very slow ~1.2 km advance in glacier terminus during 1992-2016 but in recent years (2014-2016) glacier advanced ~0.3 km, intense crevasses and ice thickening at snout area. | 46, Modified by present study |
| 143 | G076400E35967N | Unnamed | Main | Karakoram | 35.967 | 76.400 | 5.0 | 3.4 | 4429 | 6383 | 6 | 10 | 2000-2010 | 3.7 | Moraine | 567±57 | High surface velocity, about 0.5 km advance in glacier terminus during 2000-2010, crevasses and potholes. | 46, Modified by present study |
| 144 | G076409E36001N | Unnamed | Main | Karakoram | 36.001 | 76.409 | 6.1 | 4.1 | 4098 | 6467 | 1b | 8 | 2003-2011 | 0.0 | Clean ice | 1277±57 | High surface velocity, about 1.3 km advance in glacier terminus during 2003-2011 and crevasses. | 46, Modified by present study |
| 145 | G076305E35926N | Sarpo Laggo | Main | Karakoram | 35.926 | 76.305 | 25.5 | 98.3 | 4175 | 7013 | 2b | 5 | 1993-1998 | 13.9 | Ablation zone | - | High surface velocity, shear margin, crevasses and deformed moraines. | 9, Modified by present study |
| 146 | G076283E35944N | Unnamed tributary of Sarpo Laggo | Tributary | Karakoram | 35.944 | 76.283 | 3.7 | 6.0 | 4919 | 6466 | 4c | - | 1970s | 2.7 | Moraine | - | Glacier in forward position in 1977, with some folding of terminal moraines; rapid retreat of ~2±0.12 km between 1977 and 1990 when nearby glaciers advancing. Ice thickening at snout area observed from 2013 to 2016. | 9, Modified by present study |
| 147 | G076312E35979N | Unnamed | Tributary | Karakoram | 35.979 | 76.312 | 5.9 | 10.3 | 4689 | 6462 | 2c | 9 | 1991-2000 | 6.9 | Moraine | 2425±57 | Terminus advance of 2.4 km between 1991 and 2000, crevasses and looped moraine. | 9, 25, 27, Modified by present study |
| 148 | G076270E36018N | Unnamed | Main | Karakoram | 36.018 | 76.270 | 6.1 | 7.8 | 4156 | 6290 | 2c | 17 | 1991-2008 | 5.1 | Moraine | 2157±57 | Terminus advance of ~2.1 km between 1991 and 2008, crevasses and ice thickening at snout area. | 46, Modified by present study |
| 149 | G076438E35963N | North East Chongtar | Tributary | Karakoram | 35.963 | 76.438 | 9.4 | 76.0 | 4142 | 8554 | 3a | 3 | 2011-2014 | 11.2 | Ablation zone | - | High surface velocity, advance of ~0.8 km in glacier front during 2002-2013, crevasses, deformed moraines and shear margin. | 9, Modified by present study |
| 150 | G076522E35968N | North West Skyang (Sughet) | Main | Karakoram | 35.968 | 76.522 | 10.5 | 18.1 | 4668 | 7486 | 1b | 8 | 2005-2013 | 3.6 | Moraine | 2219±57 | Advanced position and folded moraines in 1971 and retreated by 1979. About 2.2 km advance in glacier terminus during 2005-2013 and crevasses. | 9, Modified by present study |
| 151 | G076411E36320N | Tatulu Guo | Main | Aghil Range | 36.320 | 76.411 | 10.4 | 30.5 | 4714 | 6712 | 1b | 11 | 2000-2011 | 1.3 | Moraine | - | ~1.5 km advance in glacier terminus (2000-2014), crevasses and deformed moraines. | 24, 45, Modified by present study |
| 152 | G076538E36224N | Unnamed | Main | Aghil Range | 36.224 | 76.538 | 5.0 | 3.0 | 4235 | 5931 | 1b | - | Sometime between 2000 and 2014 | 7.4 | Moraine | - | Terminus advance of ~1.0 km between 1991 and 2013, crevasses and lobe-shaped terminus. | Present study |
| 153 | G076768E36067N | Unnamed | Main | Aghil Range | 36.067 | 76.768 | 5.6 | 6.1 | 4847 | 6558 | 1b | - | Sometime between 1993 and 2013 | 4.8 | Ablation zone | - | ~1.0 km advance in glacier terminus sometime between 1993 and 2013, and intense crevasses. | Present study |
| 154 | G076803E36051N | Unnamed | Main | Aghil Range | 36.051 | 76.803 | 4.3 | 6.6 | 4938 | 6224 | 1b | - | Sometime between 2004 and 2014 | 4.0 | Moraine | - | High surface velocity, ~1.0 km advance in glacier terminus during 2004-2014, crevasses and ice thickening at snout area. | Present study |
| 155 | G076709E35851N | North Gasherbrum | Main | Karakoram | 35.851 | 76.709 | 23.8 | 108.5 | 3908 | 7976 | 1b | 5 | Sometime between 1978 and 1990, again surged during 2004-2009 | 8.8 | Ablation zone | 226±57 | Surged sometime between 1978 and 1990, again surged during 2004-2009 (Present study) and folded moraines in ablation area, thickening and surge-like behavior of upper glacier. | 9, 24, 25, 37, 45, Modified by present study |
| 156 | G076735E35773N | Sagan | Tributary | Karakoram | 35.773 | 76.735 | 10.2 | 14.2 | 4548 | 6133 | 3a | 4 | 1993-1997 | 4.2 | Moraine | 486±57 | High surface velocity during 1993-1997, advance in glacier terminus, crevasses and folded moraine. | 9, Modified by present study |
| 157 | G076768E35751N | Urdok | Main | Karakoram | 35.751 | 76.768 | 26.0 | 67.2 | 4254 | 7994 | 1b | 4 | 1993-1997 | 26.8 | Ablation zone | - | High surface velocity, advance in glacier terminus, crevasses, ice pinnacles and folded moraine. | 24, 52 |
| 158 | G076822E35747N | Staghar | Main | Karakoram | 35.747 | 76.822 | 26.0 | 77.0 | 4377 | 7135 | 1b | 2 | 1989-1991, 2009-2011 | 4.1 | Moraine | 474±57 | High surface velocity during 1989-1991 and 2009-2011, advance in glacier terminus, crevasses, and ice pinnacles. | Present study |
| 159 | G076944E35697N | Unnamed | Main | Karakoram | 35.697 | 76.944 | 8.0 | 12.5 | 4606 | 6262 | 1b | 6 | 1990-2006 | 7.1 | Moraine | 1562±57 | Front advanced in 1978, retreated 1.5±0.12 km by 1990, advanced again 1.3 km from 1990 to 1996 and crevasses. | 9, 25, Modified by present study |
| 160 | G076959E35669N | First Singhi | Tributary | Karakoram | 35.669 | 76.959 | 8.4 | 8.5 | 4671 | 6429 | 3aB | 4 | 1990-1996; 2009-2013 | 6.1 | Moraine | 1056±57 | High surface velocity, rapid front advance of ~1 km during 1991-1996 and again advanced ~0.4 during 2009-2013 and crevasses. | Present study |
| 161 | G076972E35655N | Second Singhi | Tributary | Karakoram | 35.655 | 76.972 | 7.3 | 6.0 | 4742 | 6393 | 3aB | - | - | 0.0 | Clean ice | - | Terminus advance of ~1.1 km between 1990 and 2000. | 9 |
| 162 | G077021E35681N | Singhi | Main | Karakoram | 35.681 | 77.021 | 25.0 | 109.9 | 4507 | 7428 | 4a | - | - | 6.3 | Moraine | - | Ice pinnacles, crevasses, shear margin and deformed medial moraine. | Present study |
| 163 | G077193E35613N | Kyagar | Main | Karakoram | 35.613 | 77.193 | 18.0 | 95.5 | 4753 | 7165 | 1b | 2 | 1994-1996; 2014-2016 | 2.1 | Moraine | - | Sudden advancement of terminus (by 0.3 km) during 1994-1996, ice pinnacles and deformed medial moraine. Recently again surged during 2014-2016. | Present study |
| 164 | G077380E35764N | Unnamed | Main | Aghil Range | 35.764 | 77.380 | 10.3 | 33.8 | 5111 | 6821 | 1b | 11 | 1998-2009 | 0.0 | Clean ice | - | High surface velocity during 1998-2009, ~1.0 km advance in glacier terminus, crevasses and potholes. | Present study |
| 165 | G077452E35549N | Upper Shaksgam | Main | Karakoram | 35.549 | 77.452 | 8.9 | 31.3 | 5301 | 6267 | 4a | - | - | 1.3 | Moraine | - | Folding of moraines over ablation area, shear margin and crevasses. | 9 |
| 166 | G077491E35495N | North Rimo | Main | Karakoram | 35.495 | 77.491 | 20.3 | 66.1 | 5211 | 6534 | 4a | - | - | 0.0 | Clean ice | - | Folding of moraines over ablation area, shear margin and crevasses. | 9 |
| 167 | G077533E35433N | Central Rimo | Main | Karakoram | 35.433 | 77.533 | 41.7 | 254.4 | 4959 | 7057 | 1b | 5 | 1993-1998 | 3.8 | Moraine | 888±57 | High surface velocity, about 0.9 km advance in glacier terminus during 1993-1998, crevasses, ice pinnacles and shear margin. | 9, Modified by present study |
| 168 | G077507E35371N | Unnamed tributary of South Rimo | Tributary | Karakoram | 35.371 | 77.507 | 7.0 | 15.6 | 5165 | 6184 | 3b | - | Sometime between 1979 and 1989 | 0.0 | Clean ice | - | Front advance ~2 km during 1979-1989, distortion of medial moraine and crevasses. | 9 |
| 169 | G077536E35339N | South Rimo | Main | Karakoram | 35.339 | 77.536 | 23.6 | 171.0 | 4949 | 7236 | 1b | 2 | 1989-1991 | 2.8 | Moraine | 443±57 | High surface velocity during 1989-1991, about 0.5 km advance in glacier terminus, crevasses, ice pinnacles and folded moraines. | 9, Modified by present study |
| 170 | G077578E35290N | Unnamed | Main | Karakoram | 35.290 | 77.578 | 6.7 | 17.1 | 5055 | 6427 | 1b | 6 | 2008-2014 | 0.0 | Clean ice | 1242±57 | About 1.2 km advance in glacier terminus during 2008-2014 and crevasses. | Present study |
| 171 | G077581E35234N | North Chong Kumdan | Main | Karakoram | 35.234 | 77.581 | 20.8 | 108.6 | 4668 | 7348 | 1b | 7 | 2001-2008 | 4.9 | Moraine | 1705±57 | High surface velocity during 2001-2008, about 1.7 km advance in glacier terminus, crevasses and ice pinnacles . | 9, 22, 24, Modified by present study |
| 172 | G077614E35184N | Unnamed tributary of Chong Kumdan | Tributary | Karakoram | 35.184 | 77.614 | 5.4 | 3.6 | 5094 | 6518 | 6 | 6 | 2002-2008 | 5.0 | Moraine | 294±57 | About 0.3 km advance in glacier terminus during 2003-2012, crevasses and potholes. | Present study |
| 173 | G077677E35153N | Unnamed | Main | Karakoram | 35.153 | 77.677 | 3.2 | 2.4 | 4770 | 6115 | 6 | 11 | 1989-2000 | 3.0 | Moraine | 514±57 | About 0.5 km slow advance in glacier terminus during 1989-2000, ice pinnacles and crevasses. | Present study |
| 174 | G077682E35109N | Kichik Kumdun | Main | Karakoram | 35.109 | 77.682 | 20.3 | 65.0 | 4684 | 7414 | 1a | 2 | 1998-2000 | 3.0 | Moraine | - | Reports of advances in 1920s. Terminus advance of 2.4 km in 7 months in 1935-1936. About 1.2 km advance in glacier terminus during 1998-2000, crevasses, ice pinnacles and shear margin. | 3, 9, 33, 38, 53, Modified by present study |
| 175 | G077588E35070N | Tributary of Mamostang Glacier | Tributary | Karakoram | 35.070 | 77.588 | 6.0 | 9.0 | 4797 | 6217 | 3a | - | - | 3.8 | Moraine | - | Slow terminus advance of ~0.5 km between 1990 and 2014, crevasses, deformed moraines and potholes. | Present study |
| 176 | G077690E35064N | Aktash | Main | Karakoram | 35.064 | 77.690 | 14.3 | 26.0 | 4530 | 6942 | 2c | 7 | 2006-2013 | 5.9 | Moraine | 760±57 | Previous reports of surges: 1.6 km in 3 months (1868-1869); sudden, rapid advance (1902-1903); 2.5 km in 7 months (1935-1936); rapid advance (1958); ~ 0.5 km advance in glacier terminus (2007-2013). | 9, 23, 24, 25, 31, 32, 50, Modified by present study |
| 177 | G077761E35013N | Unnamed | Main | Karakoram | 35.013 | 77.761 | 8.0 | 14.4 | 4854 | 6291 | 1b | 6 | 1994-2000 | 2.6 | Moraine | 1713±57 | Rapid terminus advance of 1.7 km between 1994 and 2003, crevasses, deformed moraines and potholes. | 9 |
| 178 | G077793E35000N | Unnamed | Tributary | Karakoram | 35.000 | 77.793 | 3.1 | 2.3 | 5099 | 6096 | 3aB | 6 | 1992-1998 | 0.0 | Clean ice | 525±57 | About 0.5 km advance in glacier terminus during 1992-1998, crevasses and potholes. | Present study |
| 179 | G077682E34935N | West Chamshen | Main | Karakoram | 34.935 | 77.682 | 12.2 | 36.5 | 4762 | 6415 | 1b | 6 | 2007-2013 | 0.0 | Clean ice | 3383±57 | Rapid terminus advance by ~3.4 km between 2007 and 2013 while surrounding glaciers stable or advancing, distorted moraines and crevasses. | 9, Modified by present study |
| 180 | G077768E34911N | East Chamshen | Main | Karakoram | 34.911 | 77.768 | 13.9 | 29.9 | 4678 | 7645 | 1c | - | 1980s | 8.5 | Moraine | - | About 1.5 km terminus advance during 1978-1989, heavily crevassed surface, shear margin and potholes. | 9, Modified by present study |
| 181 | G077794E34919N | Little Chamshen | Main | Karakoram | 34.919 | 77.794 | 9.4 | 8.8 | 4749 | 6953 | 6 | 20 | 1993-2014 | 0.5 | Moraine | 425±57 | ~0.5 km advance in glacier terminus during 1993-2014, intense crevasses, ice pinnacles and potholes. | Present study |
| 182 | G077846E34912N | Tughmo Zarpo (West and East) | Main | Karakoram | 34.912 | 77.846 | 8.8 | 27.2 | 4948 | 6851 | 1b | 18 | 1990-2008 | 2.2 | Moraine | 1162±57 | ~1.2 km advance in glacier terminus during 1990-2008, intense crevasses, ice pinnacles, shear margin and potholes. | 3, 9 |
| 183 | G077962E34894N | Sultan Chhusshko | Main | Karakoram | 34.894 | 77.962 | 6.9 | 10.4 | 5359 | 6588 | 1c | - | - | 1.3 | Moraine | - | Report of enormous push forward.. 200-300 million meters of ice. Only 184±57 m advance from 2002 to 2014. | 9, 23, 24, 54 |
| 184 | G078014E34855N | Unnamed | Main | Karakoram | 34.855 | 78.014 | 4.4 | 6.1 | 4976 | 6358 | 6 | - | Between sometime 1993-2009 | 5.0 | Moraine | 393±57 | Slow advance in glacier terminus (by ~0.4 km) during 1993-2009, crevasses and potholes. | Present study |
| 185 | G078075E34836N | Unnamed | Main | Karakoram | 34.836 | 78.075 | 6.5 | 10.5 | 4914 | 6489 | 6 | 19 | 1990-2009 | 2.5 | Moraine | 671±57 | About 700 m slow advancement in glacier terminus from 1990 to 2005, crevasses, shear margin and potholes. | Present study |
| 186 | G077923E34783N | Unnamed tributary of North Shukpa Kunchang | Tributary | Karakoram | 34.783 | 77.923 | 12.4 | 35.3 | 4991 | 6762 | 3aB | 7 | 2001-2009 | 1.8 | Moraine | 1468±57 | ~1.5 km advance in glacier terminus during 2001-2009, intense crevasses and potholes. | 9, Modified by present study |
| 187 | G077810E34645N | North Tirit | Main | Karakoram | 34.645 | 77.810 | 5.3 | 12.6 | 5201 | 6399 | 6 | 16 | 1998-2014 | 1.4 | Moraine | 422±57 | ~0.5 km advance in glacier terminus, crevasses and elevation loss in accumulation zone and ice gain in ablation zone based on 2003 and 2011 Google earth satellite images. | Present study |
| 188 | G077828E34643N | Tributary of North Tirit | Tributary | Karakoram | 34.643 | 77.828 | 4.5 | 3.3 | 5239 | 6353 | 4b | 20 | 1994-2004 | 4.2 | Moraine | 387±57 | About 0.4 km advance in glacier terminus during 1994-2014, crevasses and elevation loss in accumulation zone and ice gain in ablation zone based on Google earth satellite images. | Present study |
| 189 | G077978E34594N | South East Shukpa Kunchang | Main | Karakoram | 34.594 | 77.978 | 15.6 | 48.5 | 4744 | 6659 | 1b | 3 | 2006-2009 | 2.5 | Moraine | 2008±57 | High surface velocity during 2006-2009, ~2 km advance in glacier terminus, potholes, crevasses and shear margin. | 3, 44 |
| 190 | G078004E34605N | Unnamed tributary of South East Shukpa Kuchang | Tributary | Karakoram | 34.605 | 78.004 | 5.0 | 3.2 | 4912 | 6265 | 3aB | 16 | 1998-2014 | 0.0 | Clean ice | 546±57 | About 0.5 km advance in glacier terminus during 1998-2014, crevasses and potholes. | Present study |
| 191 | G078085E34606N | Unnamed | Main | Karakoram | 34.606 | 78.085 | 8.3 | 12.3 | 5101 | 6764 | 6 | 15 | 1996-2011 | 2.2 | Moraine | - | ~0.8 km advance in glacier terminus during 1996-2011, crevasses and deformed moraine. | Present study |
| 192 | G077959E34457N | Unnamed | Main | Karakoram | 34.457 | 77.959 | 3.6 | 1.9 | 4879 | 6222 | 6 | - | - | 14.0 | Moraine | 441±57 | About 0.5 km advance in glacier terminus during 1993-2014 and crevasses. | Present study |
| 193 | G078238E34723N | Unnamed | Main | Chang Chenmo | 34.723 | 78.238 | 2.0 | 2.3 | 5000 | 6209 | 6 | 6 | 1994-2000 | 0.0 | Clean ice | 581±57 | Sudden advance (by 560) in glacier terminus during 1993-2000. | 3 |
| 194 | G078404E34588N | Unnamed | Main | Chang Chenmo | 34.588 | 78.404 | 9.0 | 20.7 | 5169 | 6557 | 1b | 9 | 1993-2004 | 1.1 | Moraine | 1449±57 | ~1.4 km glacier terminus advance during 1993-2004, crevasses, looped moraines, shear margin and potholes. | 3, 9 |
| 195 | G078351E34555N | Unnamed | Main | Chang Chenmo | 34.555 | 78.351 | 4.5 | 5.4 | 5055 | 6316 | 4c | - | - | 0.0 | Clean ice | - | Terminus retreat of ~2.9 km between 1990 and 2000 while surrounding glaciers stable or advancing. | 3, 9 |
| 196 | G078429E34523N | Unnamed | Tributary | Chang Chenmo | 34.523 | 78.429 | 6.6 | 5.9 | 5325 | 6492 | 3aB | 8 | 2005-2013 | 0.0 | Clean ice | - | High surface velocity during 2005-2013, crevasses and deformed moraines. | 3 |
| 197 | G078474E34504N | Unnamed | Main | Chang Chenmo | 34.504 | 78.474 | 8.0 | 20.9 | 5251 | 6414 | 2c | 17 | 1989-2006 | 1.4 | Moraine | 384±57 | Slow advance (by ~0.4 km) in terminus during 1989-2006, crevasses, looped moraines and shear margin. | 3, 9 |
| 198 | G078530E34403N | Unnamed | Main | Chang Chenmo | 34.403 | 78.530 | 6.6 | 18.0 | 5286 | 6321 | 4a | - | - | 0.0 | Clean ice | - | Folding of moraines over ablation area. | 3, 9 |
| 199 | G077211E34968N | Unnamed | Main | Karakoram | 34.968 | 77.211 | 4.0 | 4.1 | 4860 | 6416 | 6 | - | Sometime between 1994 and 2013 | 12.0 | Ablation zone | 341±57 | ~0.3 km advance in glacier terminus during 1994-2013, and crevasses. | Present study |
| 200 | G077271E34999N | South Kailas | Main | Karakoram | 34.999 | 77.271 | 8.6 | 12.3 | 4345 | 6558 | 6 | 14 | 1989-2003 | 8.2 | Moraine | 667±57 | About 0.7 km advance in glacier terminus during 1996-2003, crevasses and potholes. During surging glacier mass overridden the lateral moraines in receiving zone. | Present study |
| 201 | G077254E35022N | North Kailas | Main | Karakoram | 35.022 | 77.254 | 4.8 | 7.5 | 4599 | 6275 | 6 | 11 | 1989-2000 | 14.6 | Ablation zone | 362±57 | About 0.4 km slow advance in glacier terminus 1993-2009, and crevasses. | Present study |
| 202 | G077236E35055N | Shosholing | Main | Karakoram | 35.055 | 77.236 | 3.7 | 1.4 | 4216 | 6319 | 1c | 3 | 1900s | 25.0 | Ablation zone | - | Shosholing Glacier advanced rapidly for 3 years during 1900s in the Nubra valley but during 1990-2015 (slightly advance hardly ~50 m from 1996 to 1998) | 31, Modified by present study |
| 203 | G077134E35140N | Unnamed | Main | Karakoram | 35.140 | 77.134 | 5.6 | 6.7 | 4767 | 6230 | 6 | 17 | 1995-2013 | 4.9 | Ablation zone | 421±57 | ~0.4 km advance in glacier terminus from 1995 to 2013 and crevasses and potholes. | Present study |
| 204 | G077117E35171N | Dzingrulma | Main | Karakoram | 35.171 | 77.117 | 6.5 | 11.1 | 4394 | 6275 | 6 | 13 | 1989-2002 | 5.1 | Ablation zone | 529±57 | About 0.5 km advance in glacier terminus during 1989-2002, crevasses and potholes. | Present study |
| 205 | G077099E35204N | Unnamed tributary of South Hasrhat | Tributary | Karakoram | 35.204 | 77.099 | 5.6 | 6.2 | 4287 | 5934 | 3aB | 3 | 1996-1999 | 0.0 | Clean ice | 549±57 | High surface velocity during 1996-1999, about 0.6 km advance in glacier terminus and crevasses. | 9, Modified by present study |
| 206 | G077103E35285N | Unnamed | Main | Karakoram | 35.285 | 77.103 | 7.2 | 9.4 | 4701 | 6327 | 6 | 8 | 1997-2005 | 0.0 | Clean ice | 519±57 | About 0.5 km advance in glacier terminus during 1995-2006, crevasses and potholes. | Present study |
| 207 | G077188E35318N | Siachen | Main | Karakoram | 35.318 | 77.188 | 70.0 | 807.3 | 3550 | 7697 | 6 | 8 | 1990-1998 | 12.9 | Ablation zone | 217±57 | High surface velocity during 1990-1998, about 0.2 km advance in glacier terminus, crevasses, ice pinnacles, elevation gain near the terminus and shear margin. | Present study |
| 208 | G077056E35499N | Teram Shehr | Tributary | Karakoram | 35.499 | 77.056 | 43.0 | 265.6 | 4526 | 7379 | 4b | - | - | 5.7 | Moraine | - | crevasses, shear margin, looped moraines and potholes. | 9 |
| 209 | G077245E35364N | Unnamed | Main | Karakoram | 35.364 | 77.245 | 4.4 | 6.5 | 4811 | 6820 | 1b | 10 | 1989-1998 | 1.5 | Moraine | 1731±57 | High surface velocity, about 1.7 km advance in glacier terminus during 1989-1998 and 1.5 km retreated from 2001 to 2013. | Present study |
| 210 | G077247E35350N | Unnamed | Main | Karakoram | 35.350 | 77.247 | 6.0 | 4.8 | 4894 | 6548 | 1b | 2 | 1978-1980 | 1.1 | Moraine | - | About 1.0±0.12 km advance in glacier terminus during 1976-1980 and possibly continuously advance and crevasses. | Present study |
| 211 | G077297E35260N | North Terong | Main | Karakoram | 35.260 | 77.297 | 21.5 | 116.2 | 3971 | 7237 | 6 | - | - | 9.0 | Ablation zone | - | High surface velocity between 1990 and 2000, potholes, shear margin and looped moraine. | Present study |
| 212 | G077304E35217N | Unnamed | Tributary | Karakoram | 35.217 | 77.304 | 5.0 | 5.5 | 4383 | 5958 | 1b | 5 | 1976-1978;1991-1994; 2006-2011 | 6.5 | Moraine | 1525±57 | High surface velocity, about 1.5 km sudden advance in glacier terminus during 2006-2011 and crevasses. | 24, 52 |
| 213 | G077319E35209N | Unnamed | Main | Karakoram | 35.209 | 77.319 | 5.8 | 4.7 | 4459 | 6104 | 6 | 6 | 1993-1999 | 0.0 | Clean ice | 433±57 | About 0.3 km advance in glacier terminus during 1993-1999, crevasses and potholes. | Present study |
| 214 | G077335E35211N | Unnamed | Main | Karakoram | 35.211 | 77.335 | 3.6 | 1.7 | 4564 | 6024 | 6 | 13 | 1994-2007 | 0.0 | Clean ice | 328±57 | ~0.3 km slow advance in glacier terminus from 1994 to 2007, crevasses and potholes. | Present study |
| 215 | G077040E35151N | Milzakaphal | Main | Karakoram | 35.151 | 77.040 | 7.6 | 22.1 | 4508 | 6492 | 1b | - | Sometime between 1998 and 2014 | 4.9 | Ablation zone | 800±57 | ~0.8 km advance in glacier terminus during 1998-2013, crevasses and potholes. | Present study |
| 216 | G076940E35303N | Unnamed | Main | Karakoram | 35.303 | 76.940 | 5.0 | 6.8 | 4502 | 6235 | 6 | 17 | 1991-2008 | 0.0 | Clean ice | 564±57 | Slow terminus advance ~0.6 km during 1991-2008, increase in crevassing and bulbous terminus. | 24 |
| 217 | G076844E35262N | Unnamed | Main | Karakoram | 35.262 | 76.844 | 4.4 | 4.6 | 4355 | 5780 | 6 | - | Between 1997-2004 | 3.1 | Moraine | 426±57 | ~0.5 km advance in glacier terminus during 1996-2004, crevasses and lobe-shaped terminus. | Present study |
| 218 | G076820E35245N | Unnamed | Main | Karakoram | 35.245 | 76.820 | 3.1 | 3.1 | 4490 | 5586 | 6 | 5 | 1993-1998 | 0.0 | Clean ice | 243±57 | ~0.3 km advance in glacier terminus during 1990-1999, intense crevasses, potholes and lobe-shaped terminus. | Present study |
| 219 | G076829E35364N | Lica | Main | Karakoram | 35.364 | 76.829 | 7.9 | 13.5 | 4265 | 7709 | 6 | 17 | 1991-2008 | 8.2 | Ablation zone | 420±57 | Slow terminus advance ~0.5 km during 1991-2008, increase in crevassing and bulbous terminus. | 9 |
| 220 | G076815E35390N | Dong Dong | Main | Karakoram | 35.390 | 76.815 | 7.6 | 8.3 | 4131 | 7717 | 6 | - | Sometime between 1991 and 2008 | 13.5 | Ablation zone | 362±57 | ~0.4 km advance in glacier terminus sometime between 1991 and 2008, crevasses and bulbous terminus. | Present study |
| 221 | G076727E35367N | Unnamed | Main | Karakoram | 35.367 | 76.727 | 3.0 | 4.6 | 4174 | 5555 | 6 | 5 | 1992-1997 | 6.5 | Moraine | 399±57 | ~0.4 km advance in glacier terminus during 1992-1997, crevasses and bulbous terminus. | Present study |
| 222 | G076705E35546N | Kondus | Tributary | Karakoram | 35.546 | 76.705 | 27.7 | 134.9 | 3794 | 7364 | 4b | - | - | 20.5 | Ablation zone | - | High surface velocity, crevasses and deformed moraines. | 24 |
| 223 | G076534E35280N | Unnamed | Main | Karakoram | 35.280 | 76.534 | 2.6 | 1.1 | 4217 | 5218 | 6 | - | - | 15.3 | Moraine | 227±57 | Terminus advance ~0.8 km during 1990-2000 (Ref.9), but we could only found 227 m advance during similar period. Change from debris-covered terminus to bare ice. | 9, Modified by present study |

Supplementary Table S2 Surge glaciers (i.e. Surge-type, Surge like) classification and Surge-modified glaciers with selected examples. Glacier IDs are presented in Fig. 1 and Supplementary Table S1. Extended classification scheme for surge-type glaciers, surge-like accelerations, and indirect diagnostic features. Surge-type and Surge-modified glaciers are presented in Supplementary Table S3 and S5.

| **S. No.** | **Surge glaciers type** | **Selected examples** | **Total number** | **% to total number** | **Total area (km^2^)** | **% to total area** |
| --- | --- | --- | --- | --- | --- | --- |
| 1 | 1a | Hassanabad (ID 18), Karumbar (ID 4), Kutiah (ID 29), Baltar (ID 16), Yengutz Har (ID 24), Kichik Kumdun (ID 174). | 6 | 2.7 | 297.9 | 3.9 |
| 2 | 1b | Balt Bare (ID 40), Gharesa (Trivor) (ID 39), Hark (ID 2), Shahim-Dur (Saxinitulu) (ID 73), Skorga (ID 66) | 52 | 23.5 | 1639.5 | 21.2 |
| 3 | 1c | Sultan Chhusshko (ID 183), Pasu (ID 12), Niamur Gans (ID 31), Garumbar (ID 25), Sumaiyar Bur (ID 23), Minapin (ID 20). | 11 | 5.0 | 411.6 | 5.3 |
| 4 | 2a | Bualtar (ID 21), Khurdopin (ID 47). | 2 | 0.9 | 231.8 | 3.0 |
| 5 | 2b | Braldu (ID 69), Masherbrum (ID 123), Sarpo Laggo (ID 145), Momhil (ID 41). | 6 | 2.7 | 488.3 | 6.3 |
| 6 | 2c | South Chiring (Maedan) (ID 94), Shingchukpi while detached from main glacier (ID 95), North Karun Koh (ID 51), Aktash (ID 176). | 11 | 5.0 | 141.7 | 1.8 |
| 7 | 3a | Sarbzea (ID 10), Unnamed tributary of Chiring (ID 90), Skirish (ID 43), Little Skamri (ID 88), Sagan (ID 156), South Skamri (ID 87), Unnamed tributary of Dumulter Gans (ID 105), West Marpoh (ID 30), South Hainablak (ID 131), North East Chongtar (ID 149), Batura First (ID 11). | 18 | 8.1 | 766.1 | 9.9 |
| 8 | 3aA | Pumari Chhissh (ID 37), West Karumbar (ID 5). | 2 | 0.9 | 20.1 | 0.3 |
| 9 | 3aB | Liligo (ID 119)-Baltoro, Unnamed tributary of North Shukpa Kunchang (ID 186), First Singhi (ID 160) - Singhi, East lll Braldu (ID 70)- Braldu, West Chang Tok (ID 136)- Sarpo Laggo, East Mani (ID 28)- Mani, Unnamed tributary of South Hasrhat (ID 205), Unnamed tributary of North Crown (ID 75), Unnamed tributary of Sarpo Laggo (ID 138), Unnamed tributary of Skirish (ID 44), Khani Basa (ID 35), Kunyang (ID 38). | 22 | 10.0 | 578 | 7.5 |
| 10 | 3b | Unnamed tributary of South Rimo (ID 168) | 1 | 0.5 | 15.6 | 0.2 |
| 11 | 4a | Barpu (ID 22), Kuliah (Goropha) (ID 27), Kuk-KI Jerab (ID 6), Mani (ID 26), North Rimo (ID 166), Singhi (ID 162), Upper Shaksgam (ID 165), Virjerab (ID 65), Wosmi Yaz (ID 72). | 13 | 5.9 | 908.2 | 11.7 |
| 12 | 4b | Kondus (ID 222), Tributary of North Tirit (ID 188), Yukshin Gardan (ID 46), Baintha Lukpar (ID 103), Nera (ID 93), East Chang Tok (ID 137), Teram Shehr (ID 208), East Makrong (ID 33). | 11 | 5.0 | 598.3 | 7.7 |
| 13 | 4c | Muchuhar (ID 17), Shop Dur (ID 57), South Chongtar (ID 141). | 8 | 3.6 | 172.6 | 2.2 |
| 14 | 5 | Yazghil (ID 42) | 1 | 0.5 | 114.9 | 1.5 |
| 15 | 6 | Chillinji (ID 3), North Terong (ID 211), Ghondokhoro (ID 126), Siachen (ID 207), Tributary of Lokpar (ID 120). | 57 | 25.8 | 1349.4 | 17.4 |
|  |  | Total of Surge-type | 221 |  | 7734 |  |
|  |  | **Examples of Surge-modified glaciers** | **Total number** | **% to total number** | **Total area (km^2^)** | **% to total area** |
|  |  | Nobande Sobonde (Panmah) (ID 91), Hispar (ID 34) | 2 | - | 280 | - |
|  |  | Grand total of Surge-type and Surge-modified glaciers | 223 |  | 8014 |  |

Supplementary Table S3. Extended classification scheme for surge-type glaciers, surge-like accelerations, and indirect diagnostic features surge-type ice streams. The emphasis is on visible behavior and, likely to intervene in responses to or tracking of climatic inputs.

a. *Surge -type* involves well-defined surge activity of main and tributary glaciers.

b. *Surge-diagnostic* features refer to morphological and localised surface patterns identified with or thought to indicate surge activity. They include moraine ‘loops’ and ‘tear-drop’ forms (Jiskoot, 2011)^62^; breach lobes, trim-lines and sheared-off tributaries (ibid, Hewitt 2007^27^); or heavy crevassing of formerly much smoother glacier. Other potential indicators like potholes and shear margins, were also included in this survey.

c. *Surge-like* refers to marked disturbance and acceleration of ice, confined here to at least a doubling pre-event velocities, possibly affecting just a certain region of ice, or moving progressively down-glacier. They may include evidence of local thickening and over-riding of ice- margins or ‘surge bulge’ (Fig.1 f; Jiskoot. 2011)^62^.

**1. ‘Classic’ three-phase surge cycle of main glacier** and b)

**1a.** 3-phase, with build-up, sudden rapid acceleration to fast flow of relatively short duration (months), and sudden stalling or stagnation. Fast flow is one to two orders of magnitude greater than non-surge velocities in the region (>10 m d^-1^). Movement profile resembles ‘Alaska-type’,(e.g. Hassanabad, Karumbar, Kutiah). There is a massive, well-defined collapse of ice in the ‘reservoir zone’ (Fig. 1a), extreme crevassing through the fast flow area and vertical or overhanging, dirty ice at the glacier margins (Fig. 1c), large, rapid (10 m d^-1^) terminal advance (Fig 1b). Release of large quantities of turbid water have been reported (Bualtar in 1987, Karumbar in 1993).

**1b.** 3-phase with many similarities to 1a, but relatively slower acceleration to fast flow and longer duration surge (years). The quiescent phases may also be more drawn out. Resembles ‘Svalbard-type” (e.g. Balt Bare, Central Rimo*)*.

**1c.** Confirmed ‘classic’ fast flow episode of main glacier surge cycle, but insufficient satellite or ground observations to characterize build up and depletion phase (e.g. Sultan Chhushko, Pasu).

**2. Amended ‘classic’, with either one of three phases absent, or additional phase(s)** (Fig. 1.f)

**2a**. Absent: lower tongue does not stagnate or become significantly wasted during quiescent phase, and ‘normal’ flow rates (0.3-1.5 m d^-1^) may persist. There may even be short advances or retreats without apparent surging *(*e.g. Bualtar, Khurdopin).

**2b.** Absent; when a classic active fast flow surge stalls before reaching or affecting the terminus. This quiescence phase in the receiving zone tends to be complicated, with ‘noraml’ flows resumed after surging and a surge-related lagged and seemingly ‘normal’ terminus advance years later (e.g. Skamri, Braldu).

**2c.** Additional: acceleration and advance below the reservoir zone commencing some years before, and slower than the main active surge (e.g. Aktash, and Panmah-Maedan and Panmah-Shingchukpi tributaries when still detached from the main glacier).

**3. Tributary surge-types** (Fig.1.c, d)

Note: Cases of detached tributaries where surging does not reach the main glacier can be treated like ‘main glacier’ surge-types of Classes 1 or 2 (First Fareole-Panmah 2010-16).

**3a.** Cases where the active surge reaches and interacts with, usually stalling in, main glacier ice (Fig. 1c). The depletion phase involves a long drawn-out interaction of stalled but still mobile surge ice into and with the main glacier (Fig.1d). The post-surge developments overlap with and may become distinguishable from “surge-modified” features (e.g. Sarbzea, West Marpoh). A majority of these surges stall before reaching or affecting the terminus (e.g. Batura First). Where the surging tributary enters close to the main terminus it may directly cause it to advance (Lokpar-Aling).

Prior to strong interactions with the main glacier, tributary surge-types appear to behave as:

**3aA.** Classic (‘Alaska’) (e.g. Hispar-Pumari Chhissh*,* Karumbar-West Karumbar)

**3aB.** Classic (‘Svalbard’) (e.g. Baltoro-Liligo, Unnamed tributary of Shukpa Kunchang)

**3b.** Confirmed ‘classic’ active surge but insufficient information on cycle from satellite data (e.g. unnamed tributary of South Rimo).

**4. *Surge-diagnostic,* morphological and surface pattern evidence widely identified with surge activity such as episodes of pervasive and chaotic crevasse patterns (‘Crevasse/Skamri); looped and contorted medial moraines, with sub-classes:**

**4a.** Main and tributary glaciers surge-type based on well-defined surface features, although no active phase (yet) observed (e.g. Barpu, Khotia Lungma).

**4b.** No rapid movement observed, but classified as surge-type based on surface features and independent (delayed?) terminus advance (e.g. Kondus, East Makrong).

**4c** Extensive stagnation and/or retreat of ~1 km where surrounding glaciers are stable or advancing (e.g. Muchuhar, and Shop Dur).

**5. Surge-like 1: Mini-surges**

Local, partial surges or progressive accelerations, adopted when there is at least a doubling of pre-disturbance ice velocities or having post-disturbance and the acceleration affects much or the entire ablation zone. A 3-phase surge cycle may be observed, in some cases, with absent or additional elements (e.g. Bualtar in 1976), or it may not be observed (e.g. Yazghil).

**6. Surge-like 2: locally accelerated region of ice moving progressively down-glacier including kinematic waves;**

These are generally not of the higher velocities observed in classic surges and, unlike typical surges, have been observed to follow various kinds of external disturbances including heavy snowfalls, seasonal changes (BIG, 1979)^63^, and landslides onto the ice (Deline et al. 2014^64^; e.g. Chillinji, North Terong).

More examples of potentially surge-type, surge-modified glaciers or related disturbances can be found in Supplementary Table S2.

Supplementary Table S4 Repeat cycles of Surge glaciers in the Karakoram. Glaciers ID are presented in Fig. 1 and Supplementary Table S1.

| **Name** | **ID** | **GLIMS ID** | | **Glacier type** | **Region** | | **Latitude** | **Longitude** | | | **Length (Km)** | **Area (km)^2^** | | **Surge period** | | **Surge features** | **References** |
| --- | --- | --- | --- | --- | --- | --- | --- | --- | --- | --- | --- | --- | --- | --- | --- | --- | --- |
| Yazghil | 42 | G075358E36355N | | Main | Karakoram | | 36.355 | 75.358 | | | 28 | 115 | | 1990, 1998 and 2006 | | Front advanced in 1990, 1998 and 2006 (3 times), high surface velocity, ogives, potholes, crevasses, looped moraine and shear margin. | Present study |
| Kunyang | 38 | G075127E36215N | | Tributary | Karakoram | | 36.215 | 75.127 | | | 25 | 111 | | 1972-1973; 2007-2009 | | High surface velocity, advance in glacier terminus during 1972-1973 and 2007-2009, looped moraines, shear margin and crevasses. | 24, 45 |
| Unnamed ID 212 | 212 | G077304E35217N | | Tributary | Karakoram | | 35.217 | 77.304 | | | 6 | 5 | | 1976-1978;1991-1994; 2006-2010 | | High surface velocity, about 1.5 km sudden advance in glacier terminus during 2006-2011 and crevasses. | 24, 52 |
| First Feriole | 96 | G076016E35831N | | Main | Karakoram | | 35.831 | 76.016 | | | 10 | 14 | | Sometime between 1977-1990 and 2002-2014 | | Glacier in forward (surged) position in 1977, rapid retreat of 2.8 km during 1977-1990 when nearby glaciers advancing (9), rapid terminus advance ~2.4 km during 2002-2014 (Present study). | 9, 24 |
| Unnamed tributary of North Crown | 75 | G076163E36182N | | Tributary | Karakoram | | 36.182 | 76.163 | | | 10 | 23 | | Sometime between 1978-1990 and 2005-2013 | | Terminus advance of ~3.2 km from 1978 to 1990, advanced again ~1.0 km during 2005-2013, high surface velocity and crevasses. | 9, 27 |
| Drenmang | 89 | G076057E35973N | | Tributary | Karakoram | | 35.973 | 76.057 | | | 12 | 20 | | 1930-1931; 2005-2007 | | Previous surge in 1930-1931 (and adjacent Little Skamri in 1977-1978) (H98). Rapid terminus advance during 2002-2007 (Present study), looped moraines and heavily crevassed surface. | 1, 9, 24, 25, 27, 51 |
| South Chiring (Maedan) | 94 | G076051E35900N | | Tributary | Karakoram | | 35.900 | 76.051 | | | 11 | 15 | | 1860-1861; 1990-2005 | | Front advance of ~2.3 km during 1990-2006, looped and folded moraines and crevasses. | 1, 9, 18, 20, 24, 25, 48 |
| Unnamed tributary of Sarpo Laggo | 139 | G076263E35928N | | Tributary | Karakoram | | 35.928 | 76.263 | | | 6 | 4 | | Sometime between 1977-1990 and 2004-2007 | | Two rapid terminus advances of ~1.1 km each between 1977-1990 and 2004-2007, crevasses and looped moraine. | 9 |
| First Singhi | 160 | G076959E35669N | | Tributary | Karakoram | | 35.669 | 76.959 | | | 8 | 9 | | 1990-1996;2009-2013 | | High surface velocity, rapid front advance of ~1 km during 1990-1996 and again advanced ~0.4 during 2011-2013 and crevasses. | Present study |
| Aktash | 176 | G077690E35064N | | Main | Karakoram | | 35.064 | 77.690 | | | 12 | 26 | | 1868-1869; 1902-1903; 1935-1936; 1958?; 2006-2013 | | Previous reports of surges: 1.6 km in 3 months (1868-1869); sudden, rapid advance (1902-1903); 2.5 km in 7 months (1935-1936); rapid advance (1958); ~ 0.5 km advance in glacier terminus (2007-2013). | 9, 23, 24, 25, 31, 32, 50 |
| North Chong Kumdan | 171 | G077581E35234N | | Main | Karakoram | | 35.234 | 77.581 | | | 21 | 109 | | 1926; 2001-2008 | | High surface velocity during 2001-2008, about 1.7 km advance in glacier terminus, crevasses, ice pinnacles and shear margin. | 9, 22, 24 |
| Staghar | 158 | G076822E35747N | | Main | Karakoram | | 35.747 | 76.822 | | | 26 | 77 | | 1989-1991, 2009-2011 | | High surface velocity during 1989-1991 and 2009-2011, advance in glacier terminus, crevasses, ice pinnacles and folded moraine. | Present study |
| North Gasherbrum | 155 | G076709E35851N | | Main | Karakoram | | 35.851 | 76.709 | | | 23 | 108 | | Sometime between 1889&1929, Sometime between 1978 and 1990, again surged during 2003-2007 | | Surged sometime between 1978 and 1990, again surged during 2003-2007 (37) and folded moraines in ablation area, thickening and surge-like behavior of upper glacier (H98). | 11, 25, 45, 9, 37, 24 |
| Bualtar | 21 | G074759E36179N | | Main | Karakoram | | 36.179 | 74.759 | | | 23 | 69 | | 1922-1923; 1929-1930; 1986-87; 1989-1990? Continue till 2000? | | Surge began 1986-1987 after landslides onto glacier (G90), ~2 km advance 1989-1990 (H98), ~5 km advance 1990-2000 (possibly continuation of 1989-1990 surge?), crevasses, ogives and deformed moraine | 1, 9, 16, 24, 25, 35 |
| Khurdopin | 47 | G075475E36288N | | Main | Karakoram | | 36.288 | 75.475 | | | 38 | 163 | | 1978-1979, 1998-1999 (peak) | | High surface velocity during 1978-1979, 1998-1999 (peak), crevasses, ice pinnacles, shear margin, extensive looped and folded moraines. | 9, 24, 26, 28, 43 |
| Borum | 135 | G076041E35771N | | Main | Karakoram | | 35.771 | 76.041 | | | 8 | 6 | | 1977-1979 and continue but no satellite data after 79; 1991-1996; 2004-2009 | | ~1.3 km advance in glacier terminus during 1991-1996 and again advance of ~0.7 km in 2004-2009, lobe-shaped terminus and crevasses. | Present study |
| Balt Bare | 40 | G074965E36329N | | Main | Karakoram | | 36.329 | 74.965 | | | 11 | 16 | | 1976-1979 and probably continue; 2006-2012; | | High surface velocity, ~1.0 km advance in glacier terminus during 2006-2012 and crevasses. | 9, 24, 25, 56, 61 |
| Karumbar | 4 | G074144E36634N | | Main | Karakoram | | 36.634 | 74.144 | | | 21 | 82 | | 1860-1861; 1895-1905; 1930; 1955; 1993 | | Surge blocked river in 1955. Surge which started in April 1993 caused glacier advance of 7-10 m per day by June 1993 (H98). | 9, 21, 23, 24, 25, 30, 35 |
| Hassanabad | 18 | G074614E36396N | | Main | Karakoram | | 36.396 | 74.614 | | | 16 | 52 | | 1904-1905, 1972-76, 1993-2002 | | Advanced 9.7 km within 2.5 months in 1904-1905 (H07). Had broken into two tributaries Shispare and Muchuhar by 1954 due to 7 km retreat (P56). Again meet both tributaries before 1972 due to advance of Muchuhar & 1993-2002 (Present study). Shispare surged during 1972-76 and again from 1993 to 2002 (present study). However, Muchuhar could not meet with Shispare due to retreat of ~4 km. | 1, 9, 20, 23, 24, 25, 27, 29, 38, 40, 41, 58 |
| Pumari Chhissh | 37 | G075209E36140N | | Tributary of Hispar | Karakoram | | 36.140 | 75.209 | | | 8 | 14 | | 1800s; 1988-1990 | | Surge in late 1800s? (C94), looped moraines, shear margin and crevasses and rapid ~1 km advance and 20 m thickening 1988û1989 (W93) and continued till 1990 (Present study). | 1, 8, 9, 24, 25, 55 |
| Chiring | 92 | G076062E35921N | | Tributary | Karakoram | | 35.921 | 76.062 | | | 15 | 54 | | 1886-1887; 1994-1995 | | Front advance of ~2.0 km during 1990-1995, looped and folded moraines and crevasses. | 1, 9, 20, 24, 25, 27, 59, 60 |
| Kichik Kumdun | 174 | G077682E35109N | | Main | Karakoram | | 35.109 | 77.682 | | | 20 | 65 | | 1902-1903; 1935-1936; 1970-1972; 1998-2000 | | Reports of advances in 1920s (S29). Terminus advance of 2.4 km in 7 months in 1935-1936 (M40). About 1.2 km advance in glacier terminus during 1998-2000, crevasses, ice pinnacles and shear margin. | 9, 3, 33, 38, 53 |
| East Mani | 28 | G075010E35846N | | Tributary | Karakoram | | 35.846 | 75.010 | | | 9 | 13 | | Surged sometime between 1972-1976 and 1990-1994 | | High surface velocity, crevasses and ogives. | Present study |
| Darung | 100 | | G075486E35872N | Main | Karakoram | 35.871 | | | 75.486 | 2.4 | | 2.6 | Before 1977 ; 1990-1998 | | ~1.0 km retreated during 1977-1990 but probably advance began before the 1977. ~1.0 km sudden advance in glacier terminus during 1990-1999, potholes and crevasses. | | Present study |
| Unnamed ID 52 | 52 | | G075218E36752N | Main | Karakoram | 36.752 | | | 75.218 | 6.7 | | 9.8 | 1990-1996; 2015 and continue | | A large amount of ice mass transferred from the reservoir zone to receiving zone during 1990-1996 and again in 2015 and continue. | | Present study |
| Kyagar | 163 | | G077193E35613N | Main | Karakoram | 35.613 | | | 77.193 | 18.0 | | 95.5 | 1994-1996; 2014-2016 | | Sudden advancement of terminus (by 0.3 km) during 1994-1996, ice pinnacles and deformed medial moraine. Recently again surged during 2014-2016. | | Present study |
| Tonga | 101 | | G075656E35824N | Main | Karakoram | 35.824 | | | 75.656 | 6.6 | | 11.1 | 1990-1994; 2014-2016 and possibly continue | | High surface velocity, crevasses and increase of terminus thickness and advance ((by 0.3 km) during 1990-1994. | | Present study |

Supplementary Table S5: Aspects of surge-modified behaviour, interactions and disturbances between surged and non-surged ice, with examples derived mainly from recent tributary surge activity.

Surge-modified developments refer to surface patterns, morphological and debris cover features, and a range of velocity disturbances *derived from active surges in ice that does not surge*, or not in the same time frame. Such disturbances are initiated or driven by active surges but occur in other ice after, or beyond actual surging (Fig.1 c-e, and g). They may mainly involve post-surge movements or responses that introduce lag effects. Examples were observed to continue from a few weeks to several decades, notably relating to surge-type tributaries. It is likely these impacts will be increasingly affected and eventually erased by ‘normal’ dynamics of flow, ablation, and debris cover. In the interim, however, the classes and diagnostics point to modifications that will block, distort and reconfigure ice features and movements typically attributable to climatic controls. They will frustrate or complicate efforts to track them. Many if not all forms of *‘surge diagnostic’* features (Supplementary Table S3) occur with surge-modified behaviour. The most important cases for tracking glacier change are modifications that continue and evolve for years or decades after the surge. It may be that the behaviour and features of some glaciers are permanently ‘surge-modified’, disturbances persisting throughout the quiescent period of adjacent surge-type ice (Belo et al 2008)^2^. Over time, they appear as lag effects which, it is likely, climate dependent accumulation and ablation of ice mass will eventually erase. Before this occurs, they can continue for much longer than active surges, notably through the quiescent phase, and affect large areas of ice that do not surge, or did not with the main event. While they last, they are surge-derived effects that can oppose, buffer or redirect climate-driven controls and mass balance.

1. Surge modified behaviour

a) Adjustments of non-surge ice compressed, displaced, thickened, reoriented, thrust or overridden by surge ice (Examples in all recent Panmah tributary surges) ^1, 9, 20, 24, 25, 27, 59, 60^.

b) Over-riding of non-surge ice by surge ice (Chiring, Maedan, Shingchukpi and E. Skamri tributaries of Panmah, over-riding Nobande Sobonde ice (Fig 1d-e)^24, 27^.

c) Post-surge adjustments in surge ice, continuing interactions with adjacent ice movement, ablation losses and supraglacial debris build-up (Panmah tributary surge ice, 1994 to present; Hispar tributary surge ice 1993 to present)^27, 55^ .

2. Main/tributary glacier relations

a) Surge-Modified Main glaciers due to active surges of surge-type tributaries. (Examples: **Panmah Glacier** 1994-present, 4 independent tributary surges into main Nobande Sobonde. **Hispar Glacier** 1992-present, three or four tributary surges)^24, 27, 55^.

b) Surge modified tributaries due to main glacier surges. Tributary glaciers sheared off, or temporarily blocked at junction, and/or later, post- surge adjustments (Fig 1e; Panmah-Chiring).

Supplementary Table S6 Satellite data used in present study.

| **S.No.** | **Date** | **Scene ID** | **Satellite/Sensor** | **Purpose** |
| --- | --- | --- | --- | --- |
| 1 | 9/4/1972 | LM11580361972248AAA02 | Landsat1/MSS | Inventory |
| 2 | 9/6/1972 | LM11600341972250FAK05 | Landsat1/MSS | Inventory |
| 3 | 9/6/1972 | LM11600351972250AAA02 | Landsat1/MSS | Inventory |
| 4 | 9/7/1972 | LM11610351972251AAA05 | Landsat1/MSS | Inventory |
| 5 | 9/7/1972 | LM11610341972251AAA05 | Landsat1/MSS | Inventory |
| 6 | 11/16/1972 | LM11590351972321FAK03 | Landsat1/MSS | Inventory |
| 7 | 11/16/1972 | LM11590361972321AAA05 | Landsat1/MSS | Inventory |
| 8 | 9/2/1973 | LM11610351973245AAA04 | Landsat1/MSS | Inventory |
| 9 | 9/2/1973 | LM11610341973245AAA04 | Landsat1/MSS | Inventory |
| 10 | 9/19/1973 | LM11600341973262AAA02 | Landsat1/MSS | Inventory |
| 11 | 9/19/1973 | LM11600351973262AAA02 | Landsat1/MSS | Inventory |
| 12 | 9/18/1975 | LM21600341975261AAA04 | Landsat2/MSS | Inventory |
| 13 | 8/7/1976 | LM21600351976220AAA02 | Landsat2/MSS | Inventory |
| 14 | 8/8/1976 | LM21610351976221AAA05 | Landsat2/MSS | Inventory |
| 15 | 8/8/1976 | LM21610341976221AAA02 | Landsat2/MSS | Inventory |
| 16 | 8/9/1976 | LM21620341976222AAA04 | Landsat2/MSS | Inventory |
| 17 | 8/25/1976 | LM21600341976238FAK03 | Landsat2/MSS | Inventory |
| 18 | 9/10/1976 | p158r36_2m19760910 | Landsat2/MSS | Inventory |
| 19 | 9/11/1976 | LM21590361976255FAK03 | Landsat2/MSS | Inventory |
| 20 | 10/18/1976 | LM21600351976292FAK05 | Landsat2/MSS | Inventory |
| 21 | 11/3/1976 | LM21580351976308AAA02 | Landsat2/MSS | Inventory |
| 22 | 11/3/1976 | LM21580361976308AAA02 | Landsat2/MSS | Inventory |
| 23 | 11/20/1976 | p157r36_2m19761120 | Landsat2/MSS | Inventory |
| 24 | 11/21/1976 | LM21580361976326AAA03 | Landsat2/MSS | Inventory |
| 25 | 6/28/1977 | LM21610351977179AAA02 | Landsat2/MSS | Inventory |
| 26 | 6/28/1977 | p161r34_2m19770628 | Landsat2/MSS | Inventory |
| 27 | 6/28/1977 | LM21610341977179AAA04 | Landsat2/MSS | Inventory |
| 28 | 7/13/1977 | LM21580361977194XXX03 | Landsat2/MSS | Inventory |
| 29 | 7/14/1977 | LM21590351977195TGS03 | Landsat2/MSS | Inventory |
| 30 | 7/16/1977 | LM21610341977197TGS03 | Landsat2/MSS | Inventory |
| 31 | 7/17/1977 | LM21620341977198XXX03 | Landsat2/MSS | Inventory |
| 32 | 8/1/1977 | LM21590351977213FAK04 | Landsat2/MSS | Inventory |
| 33 | 8/1/1977 | LM21590361977213FAK04 | Landsat2/MSS | Inventory |
| 34 | 8/2/1977 | LM21600341977214GMD05 | Landsat2/MSS | Inventory |
| 35 | 8/2/1977 | p160r35_2m19770802 | Landsat2/MSS | Inventory |
| 36 | 8/2/1977 | LM21600351977214AAA06 | Landsat2/MSS | Inventory |
| 37 | 8/3/1977 | LM21610341977215AAA03 | Landsat2/MSS | Inventory |
| 38 | 8/4/1977 | LM21620341977216AAA03 | Landsat2/MSS | Inventory |
| 39 | 8/18/1977 | LM21580351977230AAA03 | Landsat2/MSS | Inventory |
| 40 | 8/20/1977 | p160r34_2m19770820 | Landsat2/MSS | Inventory |
| 41 | 8/20/1977 | LM21600341977232AAA06 | Landsat2/MSS | Inventory |
| 42 | 8/20/1977 | LM21600351977232FAK05 | Landsat2/MSS | Inventory |
| 43 | 8/22/1977 | LM21620341977234AAA03 | Landsat2/MSS | Inventory |
| 44 | 9/26/1977 | LM21610351977269FAK04 | Landsat2/MSS | Inventory |
| 45 | 9/26/1977 | LM21610341977269AAA03 | Landsat2/MSS | Inventory |
| 46 | 7/1/1978 | LM31600351978182AAA02 | Landsat3/MSS | Inventory |
| 47 | 7/18/1978 | p159r35_3m19780718 | Landsat3/MSS | Inventory |
| 48 | 7/18/1978 | LM31590351978199AAA04 | Landsat3/MSS | Inventory |
| 49 | 7/18/1978 | LM31590361978199AAA02 | Landsat3/MSS | Inventory |
| 50 | 7/19/1978 | LM31600351978200AAA02 | Landsat3/MSS | Inventory |
| 51 | 7/20/1978 | LM31610351978201XXX03 | Landsat3/MSS | Inventory |
| 52 | 8/7/1978 | LM31610351978219AAA02 | Landsat3/MSS | Inventory |
| 53 | 7/15/1979 | p161r35_3m19790715 | Landsat3/MSS | Inventory |
| 54 | 7/15/1979 | LM31610351979196AAA10 | Landsat3/MSS | Inventory |
| 55 | 7/31/1979 | LM31590361979212AAA05 | Landsat3/MSS | Inventory |
| 56 | 8/19/1979 | LM31600351979231AAA06 | Landsat3/MSS | Inventory |
| 57 | 9/7/1979 | LM31610351979250AAA05 | Landsat3/MSS | Inventory |
| 58 | 10/13/1979 | LM31610341979286AAA06 | Landsat3/MSS | Inventory |
| 59 | 10/14/1979 | LM31620341979287XXX01 | Landsat3/MSS | Inventory |
| 60 | 11/9/1980 | LM31580361980314AAA04 | Landsat3/MSS | Inventory |
| 61 | 9/14/1981 | LM31610341981257AAA08 | Landsat3/MSS | Inventory |
| 62 | 2/9/1989 | LT51490351989040ISP00 | Landsat 5/TM | Inventory, Surface displacement |
| 63 | 7/26/1989 | p150r34_5t19890726 | Landsat 5/TM | Inventory |
| 64 | 7/26/1989 | p150r35_5t19890726 | Landsat 5/TM | Inventory |
| 65 | 8/6/1989 | LT51470361989218ISP00 | Landsat 5/TM | Inventory, Surface displacement |
| 66 | 10/9/1989 | p147r35_5t19891009 | Landsat 5/TM | Inventory, Surface displacement |
| 67 | 10/9/1989 | p147r36_5t19891009 | Landsat 5/TM | Inventory |
| 68 | 11/17/1989 | LT51480351989321ISP00 | Landsat 5/TM | Inventory, Surface displacement |
| 69 | 12/3/1989 | LT51480351989337ISP00 | Landsat 5/TM | Inventory, Surface displacement |
| 70 | 3/9/1990 | LT51480351990068ISP00 | Landsat 5/TM | Inventory, Surface displacement |
| 71 | 6/29/1990 | p148r35_5t19900629 | Landsat 5/TM | Inventory, Surface displacement |
| 72 | 6/29/1990 | p148r36_5t19900629 | Landsat 5/TM | Inventory |
| 73 | 8/7/1990 | LT51490351990219ISP00 | Landsat 5/TM | Inventory, Surface displacement |
| 74 | 7/2/1991 | LT51480351991183ISP00 | Landsat 5/TM | Inventory |
| 75 | 7/2/1991 | LT51480361991183ISP00 | Landsat 5/TM | Inventory |
| 76 | 8/19/1991 | LT51480351991231ISP00 | Landsat 5/TM | Inventory, Surface displacement |
| 77 | 8/28/1991 | LT51470351991240ISP00 | Landsat 5/TM | Inventory |
| 78 | 8/28/1991 | LT51470361991240ISP00 | Landsat 5/TM | Inventory, Surface displacement |
| 79 | 11/14/1991 | LT51490341991318ISP00 | Landsat 5/TM | Inventory |
| 80 | 11/14/1991 | LT51490351991318ISP00 | Landsat 5/TM | Inventory |
| 81 | 12/2/1991 | LT51470351991336ISP00 | Landsat 5/TM | Inventory |
| 82 | 6/2/1992 | LT51480351992154ISP00 | Landsat 5/TM | Inventory, Surface displacement |
| 83 | 8/14/1992 | LT51470351992227ISP00 | Landsat 5/TM | Inventory |
| 84 | 10/10/1992 | p146r36_5t19921010 | Landsat 5/TM | Inventory |
| 85 | 10/15/1992 | p149r34_5t19921015 | Landsat 5/TM | Inventory, Surface displacement |
| 86 | 10/15/1992 | p149r35_5t19921015 | Landsat 5/TM | Inventory |
| 87 | 7/29/1992 | LT51470361991240ISP00 | Landsat 5/TM | Inventory, Surface displacement |
| 88 | 11/25/1992 | LT51480361992330ISP00 | Landsat 5/TM | Inventory, Surface displacement |
| 89 | 12/20/1992 | LT51470351992355ISP00 | Landsat 5/TM | Inventory |
| 90 | 12/20/1992 | LT51470361992355ISP00 | Landsat 5/TM | Inventory |
| 91 | 4/27/1993 | LT51470361993117ISP00 | Landsat 5/TM | Inventory |
| 92 | 5/13/1993 | LT51470361993133ISP00 | Landsat 5/TM | Inventory |
| 93 | 6/12/1993 | LT51490351993163ISP00 | Landsat 5/TM | Inventory, Surface displacement |
| 94 | 6/19/1993 | LT51500351993170ISP00 | Landsat 5/TM | Inventory |
| 95 | 7/5/1993 | LT51500341993186ISP00 | Landsat 5/TM | Inventory |
| 96 | 7/5/1993 | LT51500351993186ISP00 | Landsat 5/TM | Inventory |
| 97 | 9/2/1993 | LT51470361993245ISP00 | Landsat 5/TM | Inventory, Surface displacement |
| 98 | 7/7/1993 | LT51480351993188ISP00 | Landsat 5/TM | Inventory, Surface displacement |
| 99 | 7/7/1993 | LT51480361993188ISP00 | Landsat 5/TM | Inventory |
| 100 | 7/21/1993 | LT51500351993202ISP00 | Landsat 5/TM | Inventory |
| 101 | 8/6/1993 | LT51500341993218ISP00 | Landsat 5/TM | Inventory |
| 102 | 12/14/1993 | LT51480351993348ISP00 | Landsat 5/TM | Inventory |
| 103 | 6/1/1994 | LT51470351994152ISP00 | Landsat 5/TM | Inventory |
| 104 | 6/1/1994 | LT51470361994152ISP00 | Landsat 5/TM | Inventory |
| 105 | 6/24/1994 | LT51480351994175ISP00 | Landsat 5/TM | Inventory, Surface displacement |
| 106 | 7/1/1994 | LT51490351994182ISP00 | Landsat 5/TM | Inventory, Surface displacement |
| 107 | 7/8/1994 | LT51500341994189ISP00 | Landsat 5/TM | Inventory |
| 108 | 7/8/1994 | LT51500351994189ISP00 | Landsat 5/TM | Inventory |
| 109 | 7/17/1994 | LT51490341994198ISP00 | Landsat 5/TM | Inventory |
| 110 | 7/17/1994 | LT51490351994198ISP00 | Landsat 5/TM | Inventory, Surface displacement |
| 111 | 9/3/1994 | LT51490341994246ISP00 | Landsat 5/TM | Inventory |
| 112 | 9/3/1994 | LT51490351994246ISP00 | Landsat 5/TM | Inventory |
| 113 | 10/23/1994 | LT51470351994296ISP00 | Landsat 5/TM | Inventory, Surface displacement |
| 114 | 10/23/1994 | LT51470361994296ISP00 | Landsat 5/TM | Inventory, Surface displacement |
| 115 | 11/15/1994 | LT51480351994319ISP00 | Landsat 5/TM | Inventory |
| 116 | 11/22/1994 | LT51490341994326ISP00 | Landsat 5/TM | Inventory |
| 117 | 3/16/1995 | LT51470351995075ISP00 | Landsat 5/TM | Inventory |
| 118 | 11/11/1995 | LT51470361995315ISP00 | Landsat 5/TM | Inventory, Surface displacement |
| 119 | 11/21/1995 | LT51480351995306ISP00 | Landsat 5/TM | Inventory, Surface displacement |
| 120 | 7/6/1996 | LT51490341996188ISP00 | Landsat 5/TM | Inventory, Surface displacement |
| 121 | 7/6/1996 | LT51490351996188ISP00 | Landsat 5/TM | Inventory, Surface displacement |
| 122 | 7/8/1996 | LT51470351996190ISP00 | Landsat 5/TM | Inventory |
| 123 | 7/8/1996 | LT51470361996190ISP00 | Landsat 5/TM | Inventory |
| 124 | 7/29/1996 | LT51500341996211ISP00 | Landsat 5/TM | Inventory |
| 125 | 7/29/1996 | LT51500351996211ISP00 | Landsat 5/TM | Inventory |
| 126 | 7/31/1996 | LT51480361996213ISP00 | Landsat 5/TM | Inventory, Surface displacement |
| 127 | 9/1/1996 | LT51480351996245ISP00 | Landsat 5/TM | Inventory, Surface displacement |
| 128 | 9/10/1996 | LT51470361996254ISP00 | Landsat 5/TM | Inventory, Surface displacement |
| 129 | 10/10/1996 | LT51490351996284ISP00 | Landsat 5/TM | Inventory |
| 130 | 11/13/1996 | LT51470351996318ISP00 | Landsat 5/TM | Inventory |
| 131 | 11/13/1996 | LT51470361996318ISP00 | Landsat 5/TM | Inventory |
| 132 | 7/18/1997 | LT51480351997199ISP00 | Landsat 5/TM | Inventory, Surface displacement |
| 133 | 8/10/1997 | LT51490351997222SGI00 | Landsat 5/TM | Inventory, Surface displacement |
| 134 | 9/4/1997 | LT51480351997247ISP00 | Landsat 5/TM | Inventory |
| 135 | 10/4/1997 | LT51500341997277ISP00 | Landsat 5/TM | Inventory |
| 136 | 10/4/1997 | LT51500351997277ISP00 | Landsat 5/TM | Inventory |
| 137 | 7/19/1998 | LT51500341998200XXX02 | Landsat 5/TM | Inventory |
| 138 | 7/28/1998 | LT51490351998209XXX01 | Landsat 5/TM | Inventory |
| 139 | 8/4/1998 | LT51500351998216XXX01 | Landsat 5/TM | Inventory |
| 140 | 8/13/1998 | LT51490341998225XXX01 | Landsat 5/TM | Inventory |
| 141 | 8/13/1998 | LT51490351998225XXX01 | Landsat 5/TM | Inventory, Surface displacement |
| 142 | 8/20/1998 | LT51500341998232BIK00 | Landsat 5/TM | Inventory |
| 143 | 8/29/1998 | LT51490351998241BIK03 | Landsat 5/TM | Inventory |
| 144 | 9/7/1998 | LT51480361998250XXX01 | Landsat 5/TM | Inventory, Surface displacement |
| 145 | 9/16/1998 | L5147036_03619980916 | Landsat 5TM | Inventory, Surface displacement |
| 146 | 10/9/1998 | LT51480351998282XXX02 | Landsat 5/TM | Inventory |
| 147 | 10/16/1998 | LT51490341998289XXX01 | Landsat 5/TM | Inventory |
| 148 | 7/7/1999 | LE71490341999188EDC00 | Landsat 7/ETM+ | Inventory |
| 149 | 7/7/1999 | LE71490351999188EDC00 | Landsat 7/ETM+ | Inventory |
| 150 | 7/9/1999 | LE71470361999190SGS00 | Landsat 7/ETM+ | Inventory |
| 151 | 7/16/1999 | LE71480351999197EDC00 | Landsat 7/ETM+ | Inventory, Surface displacement |
| 152 | 7/16/1999 | LE71480361999197EDC00 | Landsat 7/ETM+ | Inventory |
| 153 | 7/23/1999 | LE71490341999204AGS00 | Landsat 7/ETM+ | Inventory |
| 154 | 7/23/1999 | LE71490351999204AGS00 | Landsat 7/ETM+ | Inventory |
| 155 | 8/9/1999 | LT51480361999221XXX01 | Landsat 5/TM | Inventory |
| 156 | 8/15/1999 | LE71500341999227EDC01 | Landsat 7/ETM+ | Inventory |
| 157 | 8/16/1999 | LT51490341999228AAA02 | Landsat 5/TM | Inventory |
| 158 | 8/16/1999 | LT51490351999228AAA02 | Landsat 5/TM | Inventory, Surface displacement |
| 159 | 8/24/1999 | LE71490351999236EDC00 | Landsat 7/ETM+ | Inventory, Surface displacement |
| 160 | 9/16/1999 | LE71500351999259SGS00 | Landsat 7/ETM+ | Inventory |
| 161 | 10/29/1999 | L71147036_03619991029 | Landsat 7/ETM+ | Inventory, Surface displacement |
| 162 | 5/6/2000 | LE71490352000127SGS01 | Landsat 7/ETM+ | Inventory |
| 163 | 5/14/2000 | LT51490342000135AAA02 | Landsat 5/TM | Inventory |
| 164 | 5/14/2000 | LT51490352000135AAA02 | Landsat 5/TM | Inventory |
| 165 | 5/21/2000 | LT51500342000142XXX02 | Landsat 5/TM | Inventory |
| 166 | 5/21/2000 | LT51500352000142XXX02 | Landsat 5/TM | Inventory |
| 167 | 6/14/2000 | LE71500342000166SGS00 | Landsat 7/ETM+ | Inventory |
| 168 | 7/9/2000 | LE71490352000191SGS00 | Landsat 7/ETM+ | Inventory |
| 169 | 8/17/2000 | LE71500342000230SGS00 | Landsat 7/ETM+ | Inventory |
| 170 | 8/17/2000 | LE71500352000230SGS00 | Landsat 7/ETM+ | Inventory |
| 171 | 8/26/2000 | LE71490352000239SGS00 | Landsat 7/ETM+ | Inventory |
| 172 | 8/27/2000 | LT51480352000240XXX02 | Landsat 5/TM | Inventory |
| 173 | 8/27/2000 | LT51480362000240XXX02 | Landsat 5/TM | Inventory |
| 174 | 8/28/2000 | L71147036_03620000828 | Landsat 7/ETM+ | Inventory, Surface displacement |
| 175 | 9/2/2000 | LE71500342000246SGS01 | Landsat 7/ETM+ | Inventory |
| 176 | 9/2/2000 | LE71500352000246SGS01 | Landsat 7/ETM+ | Inventory |
| 177 | 9/4/2000 | LE71480352000248SGS00 | Landsat 7/ETM+ | Inventory |
| 178 | 9/4/2000 | LE71480362000248SGS00 | Landsat 7/ETM+ | Inventory |
| 179 | 9/11/2000 | LE71490342000255SGS00 | Landsat 7/ETM+ | Inventory |
| 180 | 9/11/2000 | LE71490352000255SGS00 | Landsat 7/ETM+ | Inventory, Surface displacement |
| 181 | 10/29/2000 | LE71490342000303SGS00 | Landsat 7/ETM+ | Inventory |
| 182 | 10/29/2000 | p149r035_7t20001029 | Landsat 7/ETM+ | Inventory |
| 183 | 6/1/2001 | LE71500352001152SGS00 | Landsat 7/ETM+ | Inventory |
| 184 | 6/10/2001 | LE71490352001161SGS00 | Landsat 7/ETM+ | Inventory |
| 185 | 6/19/2001 | LE71480362001170SGS00 | Landsat 7/ETM+ | Inventory, Surface displacement |
| 186 | 6/26/2001 | LE71490352001177SGS00 | Landsat 7/ETM+ | Inventory |
| 187 | 7/21/2001 | LE71480352001202SGS00 | Landsat 7/ETM+ | Inventory, Surface displacement |
| 188 | 8/29/2001 | LE71490352001241SGS00 | Landsat 7/ETM+ | Inventory, Surface displacement |
| 189 | 9/5/2001 | LE71500342001248SGS00 | Landsat 7/ETM+ | Inventory |
| 190 | 9/23/2001 | LE71480362001266EDC00 | Landsat 7/ETM+ | Inventory |
| 191 | 9/25/2001 | LE71460362001268EDC00 | Landsat 7/ETM+ | Inventory |
| 192 | 9/30/2001 | LE71490342001273EDC01 | Landsat 7/ETM+ | Inventory |
| 193 | 9/30/2001 | LE71490352001273EDC01 | Landsat 7/ETM+ | Inventory |
| 194 | 9/30/2001 | p149r036_7t20010930 | Landsat 7/ETM+ | Inventory |
| 195 | 10/7/2001 | LE71500342001280SGS00 | Landsat 7/ETM+ | Inventory |
| 196 | 10/7/2001 | LE71500352001280SGS00 | Landsat 7/ETM+ | Inventory |
| 197 | 7/15/2002 | LE71490352002196SGS00 | Landsat 7/ETM+ | Inventory, Surface displacement |
| 198 | 8/2/2002 | L71147036_03620020802 | Landsat 7/ETM+ | Inventory, Surface displacement |
| 199 | 8/7/2002 | LE71500342002219SGS00 | Landsat 7/ETM+ | Inventory |
| 200 | 8/9/2002 | LE71480352002221SGS00 | Landsat 7/ETM+ | Inventory |
| 201 | 8/16/2002 | LE71490342002228SGS00 | Landsat 7/ETM+ | Inventory |
| 202 | 8/16/2002 | LE71490352002228SGS00 | Landsat 7/ETM+ | Inventory, Surface displacement |
| 203 | 8/23/2002 | LE71500342002235SGS00 | Landsat 7/ETM+ | Inventory |
| 204 | 8/23/2002 | LE71500352002235SGS01 | Landsat 7/ETM+ | Inventory |
| 205 | 10/12/2002 | LE71480362002285SGS00 | Landsat 7/ETM+ | Inventory, Surface displacement |
| 206 | 5/31/2003 | LE71490342003151EDC00 | Landsat 7/ETM+ | Inventory |
| 207 | 5/31/2003 | LE71490352003151EDC00 | Landsat 7/ETM+ | Inventory, Surface displacement |
| 208 | 7/18/2003 | LE71490342003199ASN01 | Landsat 7/ETM+ | Inventory |
| 209 | 9/20/2003 | LE71490342003263ASN01 | Landsat 7/ETM+ | Inventory, Surface displacement |
| 210 | 9/22/2003 | L71147036_03620030922 | Landsat 7/ETM+ | Inventory |
| 211 | 10/15/2003 | LE71480352003288ASN03 | Landsat 7/ETM+ | Inventory, Surface displacement |
| 212 | 10/15/2003 | LE71480362003288ASN01 | Landsat 7/ETM+ | Inventory |
| 213 | 10/29/2003 | LE71500342003302ASN01 | Landsat 7/ETM+ | Inventory |
| 214 | 10/29/2003 | LE71500352003302ASN01 | Landsat 7/ETM+ | Inventory |
| 215 | 7/11/2004 | LE71500342004193ASN01 | Landsat 7/ETM+ | Inventory |
| 216 | 7/11/2004 | LE71500352004193ASN01 | Landsat 7/ETM+ | Inventory |
| 217 | 8/5/2004 | LE71490342004218PFS01 | Landsat 7/ETM+ | Inventory |
| 218 | 8/5/2004 | LE71490352004218PFS01 | Landsat 7/ETM+ | Inventory, Surface displacement |
| 219 | 8/14/2004 | LE71480352004227PFS01 | Landsat 7/ETM+ | Inventory, Surface displacement |
| 220 | 8/14/2004 | LE71480362004227PFS01 | Landsat 7/ETM+ | Inventory |
| 221 | 8/28/2004 | LE71500342004241SGS01 | Landsat 7/ETM+ | Inventory |
| 222 | 9/8/2004 | L71147036_03620040908 | Landsat 7/ETM+ | Inventory |
| 223 | 9/13/2004 | LE71500342004257PFS01 | Landsat 7/ETM+ | Inventory |
| 224 | 9/13/2004 | LE71500352004257PFS01 | Landsat 7/ETM+ | Inventory |
| 225 | 11/9/2004 | LE71490342004314PFS00 | Landsat 7/ETM+ | Inventory |
| 226 | 8/15/2005 | LE71500342005227PFS00 | Landsat 7/ETM+ | Inventory |
| 227 | 8/15/2005 | LE71500352005227PFS00 | Landsat 7/ETM+ | Inventory |
| 228 | 8/24/2005 | LE71490342005236PFS00 | Landsat 7/ETM+ | Inventory |
| 229 | 8/26/2005 | LE71470362005238PFS00 | Landsat 7/ETM+ | Inventory |
| 230 | 9/2/2005 | LE71480352005245ASN00 | Landsat 7/ETM+ | Inventory, Surface displacement |
| 231 | 9/2/2005 | LE71480362005245ASN00 | Landsat 7/ETM+ | Inventory |
| 232 | 9/16/2005 | LE71500342005259PFS00 | Landsat 7/ETM+ | Inventory |
| 233 | 9/16/2005 | LE71500352005259PFS00 | Landsat 7/ETM+ | Inventory |
| 234 | 5/23/2006 | LE71490342006143PFS00 | Landsat 7/ETM+ | Inventory |
| 235 | 6/21/2005 | LE71490352005172ASN00 | Landsat 7/ETM+ | Inventory, Surface displacement |
| 236 | 6/24/2006 | LE71490342006175ASN00 | Landsat 7/ETM+ | Inventory |
| 237 | 6/24/2006 | LE71490352006175ASN00 | Landsat 7/ETM+ | Inventory |
| 238 | 7/10/2006 | LE71490342006191PFS00 | Landsat 7/ETM+ | Inventory |
| 239 | 7/10/2006 | LE71490352006191ASN00 | Landsat 7/ETM+ | Inventory |
| 240 | 7/26/2006 | LE71490342006207PFS00 | Landsat 7/ETM+ | Inventory |
| 241 | 7/26/2006 | LE71490352006207PFS00 | Landsat 7/ETM+ | Inventory |
| 242 | 8/2/2006 | LE71500342006214ASN00 | Landsat 7/ETM+ | Inventory |
| 243 | 8/2/2006 | LE71500352006214ASN00 | Landsat 7/ETM+ | Inventory |
| 244 | 8/20/2006 | LE71480352006232PFS00 | Landsat 7/ETM+ | Inventory, Surface displacement |
| 245 | 8/27/2006 | LE71490352006239PFS00 | Landsat 7/ETM+ | Inventory, Surface displacement |
| 246 | 9/12/2006 | LE71490352006255PFS00 | Landsat 7/ETM+ | Inventory |
| 247 | 9/30/2006 | L71147036_03620060930 | Landsat 7/ETM+ | Inventory |
| 248 | 10/5/2006 | LE71500352006278PFS00 | Landsat 7/ETM+ | Inventory |
| 249 | 10/7/2006 | LE71480362006280PFS00 | Landsat 7/ETM+ | Inventory |
| 250 | 6/11/2007 | LE71490342007162ASN00 | Landsat 7/ETM+ | Inventory |
| 251 | 6/27/2007 | LE71490342007178ASN00 | Landsat 7/ETM+ | Inventory |
| 252 | 6/27/2007 | LE71490352007178ASN00 | Landsat 7/ETM+ | Inventory |
| 253 | 7/20/2007 | LE71500342007201ASN00 | Landsat 7/ETM+ | Inventory |
| 254 | 7/20/2007 | LE71500352007201ASN00 | Landsat 7/ETM+ | Inventory |
| 255 | 7/29/2007 | LE71490342007210ASN00 | Landsat 7/ETM+ | Inventory |
| 256 | 7/29/2007 | LE71490352007210ASN00 | Landsat 7/ETM+ | Inventory |
| 257 | 8/16/2007 | L71147036_03620070816 | Landsat 7/ETM+ | Inventory |
| 258 | 8/23/2007 | LE71480362007235PFS00 | Landsat 7/ETM+ | Inventory, Surface displacement |
| 259 | 8/30/2007 | LE71490352007242PFS00 | Landsat 7/ETM+ | Inventory |
| 260 | 9/15/2007 | LE71490352007258PFS00 | Landsat 7/ETM+ | Inventory, Surface displacement |
| 261 | 11/27/2007 | LE71480352007331SGS00 | Landsat 7/ETM+ | Inventory |
| 262 | 11/27/2007 | LE71480362007331SGS00 | Landsat 7/ETM+ | Inventory |
| 263 | 6/6/2008 | LE71480352008158PFS00 | Landsat 7/ETM+ | Inventory, Surface displacement |
| 264 | 6/20/2008 | LE71500342008172ASN00 | Landsat 7/ETM+ | Inventory |
| 265 | 6/20/2008 | LE71500352008172ASN00 | Landsat 7/ETM+ | Inventory |
| 266 | 7/15/2008 | LE71490342008197PFS00 | Landsat 7/ETM+ | Inventory |
| 267 | 7/31/2008 | LE71490342008213ASN00 | Landsat 7/ETM+ | Inventory |
| 268 | 7/31/2008 | LE71490352008213ASN00 | Landsat 7/ETM+ | Inventory, Surface displacement |
| 269 | 8/2/2008 | LE71470362008215ASN00 | Landsat 7/ETM+ | Inventory |
| 270 | 8/7/2008 | LE71500342008220SGS00 | Landsat 7/ETM+ | Inventory |
| 271 | 8/7/2008 | LE71500352008220SGS01 | Landsat 7/ETM+ | Inventory |
| 272 | 8/25/2008 | LE71480362008238SGS00 | Landsat 7/ETM+ | Inventory |
| 273 | 10/3/2008 | LE71490342008277PFS00 | Landsat 7/ETM+ | Inventory |
| 274 | 10/3/2008 | LE71490352008277PFS00 | Landsat 7/ETM+ | Inventory |
| 275 | 10/12/2008 | LE71480362008286PFS00 | Landsat 7/ETM+ | Inventory |
| 276 | 7/1/2009 | LT51500342009182KHC00 | Landsat 5/TM | Inventory |
| 277 | 7/18/2009 | LE71490342009199ASN00 | Landsat 7/ETM+ | Inventory |
| 278 | 7/26/2009 | LT51490342009207KHC00 | Landsat 5/TM | Inventory |
| 279 | 7/26/2009 | LT51490352009207KHC00 | Landsat 5/TM | Inventory |
| 280 | 8/2/2009 | LT51500352009214KHC00 | Landsat 5/TM | Inventory |
| 281 | 8/3/2009 | LE71490342009215ASN00 | Landsat 7/ETM+ | Inventory |
| 282 | 8/3/2009 | LE71490352009215ASN00 | Landsat 7/ETM+ | Inventory |
| 283 | 8/4/2009 | LT51480352009216KHC00 | Landsat 5/TM | Inventory, Surface displacement |
| 284 | 8/11/2009 | LT51490342009223KHC00 | Landsat 5/TM | Inventory |
| 285 | 8/11/2009 | LT51490352009223KHC00 | Landsat 5/TM | Inventory, Surface displacement |
| 286 | 8/12/2009 | LE71480352009224SGS00 | Landsat 7/ETM+ | Inventory |
| 287 | 8/12/2009 | LE71480362009224SGS00 | Landsat 7/ETM+ | Inventory |
| 288 | 8/27/2009 | LT51490352009239KHC00 | Landsat 5/TM | Inventory |
| 289 | 9/11/2009 | LE71500342009254SGS02 | Landsat 7/ETM+ | Inventory |
| 290 | 9/11/2009 | LE71500352009254SGS02 | Landsat 7/ETM+ | Inventory |
| 291 | 9/19/2009 | LT51500342009262KHC00 | Landsat 5/TM | Inventory |
| 292 | 9/19/2009 | LT51500352009262KHC00 | Landsat 5/TM | Inventory |
| 293 | 9/20/2009 | LE71490352009263SGS00 | Landsat 7/ETM+ | Inventory |
| 294 | 9/30/2009 | L5147036_03620090930 | Landsat 5 | Inventory |
| 295 | 10/7/2009 | LT51480362009280KHC00 | Landsat 5/TM | Inventory |
| 296 | 6/20/2010 | LT51480362010171KHC00 | Landsat 5/TM | Inventory |
| 297 | 8/21/2010 | LT51500342010233KHC02 | Landsat 5/TM | Inventory |
| 298 | 8/21/2010 | LT51500352010233KHC02 | Landsat 5/TM | Inventory |
| 299 | 8/22/2010 | LE71490342010234EDC00 | Landsat 7/ETM+ | Inventory |
| 300 | 8/22/2010 | LE71490352010234EDC00 | Landsat 7/ETM+ | Inventory |
| 301 | 8/23/2010 | LT51480352010235KHC00 | Landsat 5/TM | Inventory, Surface displacement |
| 302 | 8/29/2010 | LE71500352010241EDC00 | Landsat 7/ETM+ | Inventory |
| 303 | 8/31/2010 | LE71480362010243EDC00 | Landsat 7/ETM+ | Inventory |
| 304 | 9/17/2010 | L5147036_03620100917 | Landsat 5/TM | Inventory |
| 305 | 10/3/2010 | L5147036_03620101003 | Landsat 5/TM | Inventory |
| 306 | 10/9/2010 | LE71490352010282SGS00 | Landsat 7/ETM+ | Inventory |
| 307 | 10/16/2010 | LE71500342010289SGS00 | Landsat 7/ETM+ | Inventory |
| 308 | 10/16/2010 | LE71500352010289SGS00 | Landsat 7/ETM+ | Inventory |
| 309 | 10/17/2010 | LT51490342010290KHC00 | Landsat 5/TM | Inventory |
| 310 | 10/17/2010 | LT51490352010290KHC00 | Landsat 5/TM | Inventory, Surface displacement |
| 311 | 11/1/2010 | LE71500342010305ASN00 | Landsat 7/ETM+ | Inventory |
| 312 | 11/2/2010 | LT51490352010306KHC00 | Landsat 5/TM | Inventory, Surface displacement |
| 313 | 11/10/2010 | LE71490342010314ASN00 | Landsat 7/ETM+ | Inventory |
| 314 | 11/10/2010 | LE71490352010314ASN00 | Landsat 7/ETM+ | Inventory |
| 315 | 12/11/2010 | LT51500352010345KHC00 | Landsat 5/TM | Inventory |
| 316 | 5/29/2011 | LT51490342011149KHC00 | Landsat 5/TM | Inventory |
| 318 | 5/29/2011 | LT51490352011149KHC00 | Landsat 5/TM | Inventory, Surface displacement |
| 319 | 6/7/2011 | LT51480362011158KHC03 | Landsat 5/TM | Inventory |
| 320 | 6/13/2011 | LE71500342011164ASN00 | Landsat 7/ETM+ | Inventory |
| 321 | 6/14/2011 | LT51490342011165KHC03 | Landsat 5/TM | Inventory |
| 322 | 6/14/2011 | LT51490352011165KHC03 | Landsat 5/TM | Inventory |
| 323 | 6/15/2011 | LE71480362011166ASN00 | Landsat 7/ETM+ | Inventory |
| 324 | 6/22/2011 | LE71490352011173ASN00 | Landsat 7/ETM+ | Inventory, Surface displacement |
| 325 | 7/17/2011 | LE71480352011198ASN00 | Landsat 7/ETM+ | Inventory |
| 326 | 8/2/2011 | LE71480352011214PFS00 | Landsat 7/ETM+ | Inventory |
| 327 | 8/3/2011 | L5147036_03620110803 | Landsat 5/TM | Inventory |
| 328 | 8/8/2011 | LT51500352011220KHC00 | Landsat 5/TM | Inventory |
| 329 | 8/9/2011 | LE71490342011221SGS00 | Landsat 7/ETM+ | Inventory |
| 330 | 8/9/2011 | LE71490352011221SGS00 | Landsat 7/ETM+ | Inventory |
| 331 | 8/10/2011 | LT51480352011222KHC00 | Landsat 5/TM | Inventory, Surface displacement |
| 332 | 8/24/2011 | LT51500342011236KHC00 | Landsat 5/TM | Inventory |
| 333 | 8/24/2011 | LT51500352011236KHC00 | Landsat 5/TM | Inventory |
| 334 | 9/10/2011 | LE71490342011253PFS00 | Landsat 7/ETM+ | Inventory |
| 335 | 10/3/2011 | LE71500342011276PFS00 | Landsat 7/ETM+ | Inventory |
| 336 | 10/3/2011 | LE71500352011276PFS00 | Landsat 7/ETM+ | Inventory |
| 337 | 7/3/2012 | LE71480362012185PFS00 | Landsat 7/ETM+ | Inventory |
| 338 | 7/10/2012 | LE71490342012192PFS00 | Landsat 7/ETM+ | Inventory |
| 339 | 7/10/2012 | LE71490352012192PFS00 | Landsat 7/ETM+ | Inventory |
| 340 | 7/17/2012 | LE71500342012199PFS00 | Landsat 7/ETM+ | Inventory |
| 341 | 7/28/2012 | LE71470362012210PFS00 | Landsat 7/ETM+ | Inventory |
| 342 | 8/18/2012 | LE71500352012231PFS00 | Landsat 7/ETM+ | Inventory |
| 343 | 8/20/2012 | LE71480352012233PFS00 | Landsat 7/ETM+ | Inventory |
| 344 | 8/27/2012 | LE71490342012240PFS00 | Landsat 7/ETM+ | Inventory |
| 345 | 9/12/2012 | LE71490352012256PFS00 | Landsat 7/ETM+ | Inventory, Surface displacement |
| 346 | 9/28/2012 | LE71490352012272PFS00 | Landsat 7/ETM+ | Inventory |
| 347 | 10/30/2012 | LE71490352012304PFS00 | Landsat 7/ETM+ | Inventory |
| 348 | 7/14/2013 | LC81480352013195LGN00 | Landsat 8/OLI | Inventory, Surface displacement |
| 349 | 7/14/2013 | LC81480362013195LGN00 | Landsat 8/OLI | Inventory |
| 350 | 7/28/2013 | LC81500342013209LGN00 | Landsat 8/OLI | Inventory |
| 351 | 7/28/2013 | LC81500352013209LGN00 | Landsat 8/OLI | Inventory |
| 352 | 9/7/2013 | LC81490342013250LGN00 | Landsat 8/OLI | Inventory |
| 353 | 10/8/2013 | LE71500342013281SG100 | Landsat 7/ETM+ | Inventory |
| 354 | 10/9/2013 | LC81490352013282LGN00 | Landsat 8/OLI | Inventory, Surface displacement |
| 355 | 6/8/2014 | LC81470362014159LGN00 | Landsat 8/OLI | Inventory |
| 356 | 6/15/2014 | LC81480362014166LGN00 | Landsat 8/OLI | Inventory |
| 357 | 6/29/2014 | LC81500342014180LGN00 | Landsat 8/OLI | Inventory |
| 358 | 6/29/2014 | LC81500352014180LGN00 | Landsat 8/OLI | Inventory |
| 359 | 7/1/2014 | LC81480352014182LGN00 | Landsat 8/OLI | Inventory |
| 360 | 7/8/2014 | LC81490342014189LGN00 | Landsat 8/OLI | Inventory |
| 361 | 7/8/2014 | LC81490352014189LGN00 | Landsat 8/OLI | Inventory |
| 362 | 7/24/2014 | LC81490352014205LGN00 | Landsat 8/OLI | Inventory, Surface displacement |
| 363 | 8/25/2014 | LC81490352014237LGN00 | Landsat 8/OLI | Inventory, Surface displacement |
| 364 | 9/17/2014 | LC81500342014260LGN00 | Landsat 8/OLI | Inventory |
| 365 | 9/19/2014 | LC81480352014262LGN00 | Landsat 8/OLI | Inventory |
| 366 | 9/19/2014 | LC81480362014262LGN00 | Landsat 8/OLI | Inventory |
| 367 | 9/26/2014 | LC81490342014269LGN00 | Landsat 8/OLI | Inventory |
| 368 | 9/26/2014 | LC81490352014269LGN00 | Landsat 8/OLI | Inventory |
| 369 | 9/26/2014 | LC81490362014269LGN00 | Landsat 8/OLI | Inventory |
| 370 | 10/3/2014 | LC81500342014276LGN00 | Landsat 8/OLI | Inventory |
| 371 | 10/3/2014 | LC81500352014276LGN00 | Landsat 8/OLI | Inventory |
| 372 | 10/7/2014 | LC81460362014280LGN00 | Landsat 8/OLI | Inventory |
| 373 | 12/1/2014 | LC81470352014335LGN00 | Landsat 8/OLI | Inventory |
| 374 | 12/1/2014 | LC81470362014335LGN00 | Landsat 8/OLI | Inventory |
| 375 | 12/6/2014 | LC81500342014340LGN00 | Landsat 8/OLI | Inventory |
| 376 | 7/4/2015 | LC81480352015185LGN00 | Landsat 8/OLI | Inventory, Surface displacement |
| 377 | 8/19/2015 | LC81500352015231LGN00 | Landsat 8/OLI | Inventory |
| 378 | 8/21/2015 | LC81480352015233LGN00 | Landsat 8/OLI | Inventory |
| 379 | 8/21/2015 | LC81480362015233LGN00 | Landsat 8/OLI | Inventory |
| 380 | 9/13/2015 | LC81490352015256LGN00 | Landsat 8/OLI | Inventory, Surface displacement |
| 381 | 8/30/2015 | LC81470352015242LGN00 | Landsat 8/OLI | Inventory |
| 382 | 6/4/2016 | LC81480352016156LGN00 | Landsat 8/OLI | Inventory |
| 383 | 6/13/2016 | LC81470362016165LGN00 | Landsat 8/OLI | Inventory |
| 384 | 6/8/2016 | LC81500352016170LGN00 | Landsat 8/OLI | Inventory |
| 385 | 6/20/2016 | LC81480352016172LGN00 | Landsat 8/OLI | Inventory |
| 386 | 7/29/2016 | LC81490352016211LGN00 | Landsat 8/OLI | Inventory |
| 387 | 9/8/2016 | LC81480352016252LGN00 | Landsat 8/OLI | Inventory |
| 388 | 9/17/2016 | LC81470362016261LGN00 | Landsat 8/OLI | Inventory |
| 389 | 10/1/2016 | LC81490352016275LGN00 | Landsat 8/OLI | Inventory |
| 390 | 7/20/2016 | LC81500352016202LGN00 | Landsat 8/OLI | Inventory |
| 391 | 6/23/2000 | AST_L1A_00306232000060759_20140721080738_606 | ASTER | Inventory |
| 392 | 6/23/2000 | AST_L1A_00306232000060750_20140721080728_402 | ASTER | Inventory |
| 393 | 7/9/2000 | AST_L1A_00307092000060812_20140721080728_411 | ASTER | Inventory |
| 394 | 7/9/2000 | AST_L1A_00307092000060804_20140721080738_605 | ASTER | Inventory |
| 395 | 7/18/2000 | AST_L1A_00307182000060151_20140721080728_410 | ASTER | Inventory |
| 396 | 7/18/2000 | AST_L1A_00307182000060200_20140721080738_601 | ASTER | Inventory |
| 397 | 9/4/2000 | AST_L1A_00309042000060155_20140721080728_405 | ASTER | Inventory |
| 398 | 9/11/2000 | AST_L1A_00309112000060800_20140721080738_591 | ASTER | Inventory |
| 399 | 9/11/2000 | AST_L1A_00309112000060752_20140721080738_598 | ASTER | Inventory |
| 400 | 10/13/2000 | AST_L1A_00310132000060652_20140721080728_396 | ASTER | Inventory |
| 401 | 5/18/2001 | AST_L1A_00305182001055629_20140721080745_1067 | ASTER | Inventory |
| 402 | 5/18/2001 | AST_L1A_00305182001055620_20140721080735_745 | ASTER | Inventory |
| 403 | 5/18/2001 | AST_L1A_00305182001055612_20140721080735_743 | ASTER | Inventory |
| 404 | 8/29/2001 | AST_L1A_00308292001060003_20140721080735_727 | ASTER | Inventory |
| 405 | 8/29/2001 | AST_L1A_00308292001055954_20140721080735_736 | ASTER | Inventory |
| 406 | 9/30/2001 | AST_L1A_00309302001055842_20140721080745_1084 | ASTER | Inventory |
| 407 | 9/30/2001 | AST_L1A_00309302001055833_20140721080735_738 | ASTER | Inventory |
| 408 | 11/17/2001 | AST_L1A_00311172001055656_20140721080745_1082 | ASTER | Inventory |
| 409 | 11/17/2001 | AST_L1A_00311172001055705_20140721080745_1080 | ASTER | Inventory |
| 410 | 12/28/2001 | AST_L1A_00312282001054947_20140721080745_1071 | ASTER | Inventory |
| 411 | 10/3/2002 | AST_L1A_00310032002055356_20140721080708_32760 | ASTER | Inventory |
| 412 | 10/3/2002 | AST_L1A_00310032002055404_20140721080708_32761 | ASTER | Inventory |
| 413 | 10/3/2002 | AST_L1A_00310032002055413_20140721080708_32756 | ASTER | Inventory |
| 414 | 10/10/2002 | AST_L1A_00310102002055950_20140721080708_32743 | ASTER | Inventory |
| 415 | 10/10/2002 | AST_L1A_00310102002055941_20140721080708_32752 | ASTER | Inventory |
| 416 | 4/22/2003 | AST_L1A_00304222003054706_20140717023720_29739 | ASTER | Inventory |
| 417 | 4/22/2003 | AST_L1A_00304222003054715_20140717023710_29604 | ASTER | Inventory |
| 418 | 7/18/2003 | AST_L1A_00307182003055202_20140717013709_24004 | ASTER | Inventory |
| 419 | 7/18/2003 | AST_L1A_00307182003055154_20140717013709_24008 | ASTER | Inventory |
| 420 | 9/11/2003 | AST_L1A_00309112003055813_20140717013729_24235 | ASTER | Inventory |
| 421 | 9/11/2003 | AST_L1A_00309112003055804_20140717013709_23997 | ASTER | Inventory |
| 422 | 9/20/2003 | AST_L1A_00309202003055218_20140717013729_24233 | ASTER | Inventory |
| 423 | 9/20/2003 | AST_L1A_00309202003055218_20140717013729_24233 | ASTER | Inventory |
| 424 | 10/6/2003 | AST_L1A_00310062003055246_20140717013719_24063 | ASTER | Inventory |
| 425 | 10/22/2003 | AST_L1A_00310222003055254_20140717013719_24053 | ASTER | Inventory |
| 426 | 10/22/2003 | AST_L1A_00310222003055245_20140717013719_24058 | ASTER | Inventory |
| 427 | 10/22/2003 | AST_L1A_00310222003055303_20140717013719_24048 | ASTER | Inventory |
| 428 | 10/29/2003 | AST_L1A_00310292003055903_20140717013709_23994 | ASTER | Inventory |
| 429 | 10/29/2003 | AST_L1A_00310292003055854_20140717013709_24010 | ASTER | Inventory |
| 430 | 6/11/2004 | AST_L1A_00306112004054646_20140717023710_29599 | ASTER | Inventory |
| 431 | 6/11/2004 | AST_L1A_00306112004054637_20140717023710_29594 | ASTER | Inventory |
| 432 | 7/13/2004 | AST_L1A_00307132004054628_20140717023710_29602 | ASTER | Inventory |
| 433 | 8/14/2004 | AST_L1A_00308142004054606_20140717013749_17050 | ASTER | Inventory |
| 434 | 8/14/2004 | AST_L1A_00308142004054614_20140717013749_17045 | ASTER | Inventory |
| 435 | 8/14/2004 | AST_L1A_00308142004054623_20140717013749_17040 | ASTER | Inventory |
| 436 | 9/13/2004 | AST_L1A_00309132004055809_20140717013809_17161 | ASTER | Inventory |
| 437 | 9/13/2004 | AST_L1A_00309132004055817_20140717013809_17159 | ASTER | Inventory |
| 438 | 9/13/2004 | AST_L1A_00309132004055826_20140717013759_17112 | ASTER | Inventory |
| 439 | 9/15/2004 | AST_L1A_00309152004054556_20140717013759_17111 | ASTER | Inventory |
| 440 | 9/15/2004 | AST_L1A_00309152004054604_20140717013759_17106 | ASTER | Inventory |
| 441 | 11/2/2004 | AST_L1A_00311022004054527_20140717013759_17095 | ASTER | Inventory |
| 442 | 11/2/2004 | AST_L1A_00311022004054536_20140717013749_17052 | ASTER | Inventory |
| 443 | 11/2/2004 | AST_L1A_00311022004054545_20140717013749_17035 | ASTER | Inventory |
| 444 | 10/20/2005 | AST_L1A_00310202005054549_20140717023730_29947 | ASTER | Inventory |
| 445 | 10/20/2005 | AST_L1A_00310202005054558_20140717023730_29945 | ASTER | Inventory |
| 446 | 11/5/2005 | AST_L1A_00311052005054545_20140717014700_19021 | ASTER | Inventory |
| 447 | 11/5/2005 | AST_L1A_00311052005054554_20140717014700_19020 | ASTER | Inventory |
| 448 | 11/5/2005 | AST_L1A_00311052005054603_20140717014700_19015 | ASTER | Inventory |
| 449 | 11/14/2005 | AST_L1A_00311142005053953_20140717014700_19010 | ASTER | Inventory |
| 450 | 6/17/2006 | AST_L1A_00306172006054627_20140717014719_27442 | ASTER | Inventory |
| 451 | 6/17/2006 | AST_L1A_00306172006054609_20140717014720_27457 | ASTER | Inventory |
| 452 | 6/17/2006 | AST_L1A_00306172006054618_20140717014719_27449 | ASTER | Inventory |
| 453 | 6/24/2006 | AST_L1A_00306242006055224_20140717014709_27404 | ASTER | Inventory |
| 454 | 6/24/2006 | AST_L1A_00306242006055232_20140717014710_27396 | ASTER | Inventory |
| 455 | 7/26/2006 | AST_L1A_00307262006055231_20140717014709_27398 | ASTER | Inventory |
| 456 | 7/26/2006 | AST_L1A_00307262006055240_20140717014709_27403 | ASTER | Inventory |
| 457 | 8/4/2006 | AST_L1A_00308042006054629_20140717014739_27932 | ASTER | Inventory |
| 458 | 8/11/2006 | AST_L1A_00308112006055231_20140717014730_27644 | ASTER | Inventory |
| 459 | 9/12/2006 | AST_L1A_00309122006055230_20140717014730_27639 | ASTER | Inventory |
| 460 | 9/12/2006 | AST_L1A_00309122006055221_20140717014730_27636 | ASTER | Inventory |
| 461 | 9/12/2006 | AST_L1A_00309122006055212_20140717014730_27633 | ASTER | Inventory |
| 462 | 9/28/2006 | AST_L1A_00309282006055211_20140717014730_27637 | ASTER | Inventory |
| 463 | 11/8/2006 | AST_L1A_00311082006054615_20140717014709_27382 | ASTER | Inventory |
| 464 | 11/8/2006 | AST_L1A_00311082006054558_20140717014720_27459 | ASTER | Inventory |
| 465 | 11/8/2006 | AST_L1A_00311082006054607_20140717014720_27455 | ASTER | Inventory |
| 466 | 5/1/2007 | AST_L1A_00305012007055902_20140717015700_22161 | ASTER | Inventory |
| 467 | 6/11/2007 | AST_L1A_00306112007055254_20140717015700_22222 | ASTER | Inventory |
| 468 | 6/11/2007 | AST_L1A_00306112007055245_20140717015700_22213 | ASTER | Inventory |
| 469 | 6/27/2007 | AST_L1A_00306272007055301_20140717015710_22370 | ASTER | Inventory |
| 470 | 11/9/2007 | AST_L1A_00311092007055830_20140717015710_22379 | ASTER | Inventory |
| 471 | 11/9/2007 | AST_L1A_00311092007055821_20140717015710_22369 | ASTER | Inventory |
| 472 | 11/18/2007 | AST_L1A_00311182007055227_20140717015720_22414 | ASTER | Inventory |
| 473 | 11/18/2007 | AST_L1A_00311182007055218_20140717015710_22378 | ASTER | Inventory |
| 474 | 11/27/2007 | AST_L1A_00311272007054632_20140717015700_22207 | ASTER | Inventory |
| 475 | 11/27/2007 | AST_L1A_00311272007054623_20140717015700_22199 | ASTER | Inventory |
| 476 | 12/22/2007 | AST_L1A_00312222007054035_20140717023730_29939 | ASTER | Inventory |
| 477 | 12/22/2007 | AST_L1A_00312222007054044_20140717023730_29934 | ASTER | Inventory |
| 478 | 7/31/2008 | AST_L1A_00307312008055311_20140717015710_29466 | ASTER | Inventory |
| 479 | 7/31/2008 | AST_L1A_00307312008055302_20140717015720_29509 | ASTER | Inventory |
| 480 | 9/26/2008 | AST_L1A_00309262008054713_20140717015720_29519 | ASTER | Inventory |
| 481 | 9/26/2008 | AST_L1A_00309262008054655_20140717015720_29514 | ASTER | Inventory |
| 482 | 9/26/2008 | AST_L1A_00309262008054704_20140717015720_29506 | ASTER | Inventory |
| 483 | 10/3/2008 | AST_L1A_00310032008055305_20140717015720_29521 | ASTER | Inventory |
| 484 | 10/3/2008 | AST_L1A_00310032008055314_20140717015730_29680 | ASTER | Inventory |
| 485 | 10/21/2008 | AST_L1A_00310212008054055_20140717023720_29754 | ASTER | Inventory |
| 486 | 10/21/2008 | AST_L1A_00310212008054046_20140717023720_29756 | ASTER | Inventory |
| 487 | 10/26/2008 | AST_L1A_00310262008055907_20140717015710_29459 | ASTER | Inventory |
| 488 | 10/26/2008 | AST_L1A_00310262008055859_20140717015710_29454 | ASTER | Inventory |
| 489 | 11/4/2008 | AST_L1A_00311042008055252_20140717015710_29449 | ASTER | Inventory |
| 490 | 11/4/2008 | AST_L1A_00311042008055301_20140717015710_29464 | ASTER | Inventory |
| 491 | 9/20/2009 | AST_L1A_00309202009055301_20140717020710_23105 | ASTER | Inventory |
| 492 | 9/20/2009 | AST_L1A_00309202009055252_20140717020710_23108 | ASTER | Inventory |
| 493 | 9/29/2009 | AST_L1A_00309292009054637_20140717020710_23102 | ASTER | Inventory |
| 494 | 9/29/2009 | AST_L1A_00309292009054646_20140717020710_23095 | ASTER | Inventory |
| 495 | 10/31/2009 | AST_L1A_00310312009054700_20140717023720_29745 | ASTER | Inventory |
| 496 | 10/31/2009 | AST_L1A_00310312009054651_20140717023720_29748 | ASTER | Inventory |
| 497 | 6/28/2010 | AST_L1A_00306282010054648_20140717020710_27008 | ASTER | Inventory |
| 498 | 6/28/2010 | AST_L1A_00306282010054639_20140717020700_26731 | ASTER | Inventory |
| 499 | 8/22/2010 | AST_L1A_00308222010055231_20140717020710_27003 | ASTER | Inventory |
| 500 | 8/31/2010 | AST_L1A_00308312010054630_20140717020710_27004 | ASTER | Inventory |
| 501 | 10/2/2010 | AST_L1A_00310022010054618_20140717020710_27001 | ASTER | Inventory |
| 502 | 10/9/2010 | AST_L1A_00310092010055235_20140717020700_26746 | ASTER | Inventory |
| 503 | 10/9/2010 | AST_L1A_00310092010055227_20140717020700_26748 | ASTER | Inventory |
| 504 | 10/16/2010 | AST_L1A_00310162010055843_20140717020730_27209 | ASTER | Inventory |
| 505 | 10/16/2010 | AST_L1A_00310162010055825_20140717020700_26742 | ASTER | Inventory |
| 506 | 10/16/2010 | AST_L1A_00310162010055834_20140717020700_26736 | ASTER | Inventory |
| 507 | 10/18/2010 | AST_L1A_00310182010054611_20140717020720_27069 | ASTER | Inventory |
| 508 | 10/18/2010 | AST_L1A_00310182010054620_20140717020720_27067 | ASTER | Inventory |
| 509 | 10/25/2010 | AST_L1A_00310252010055220_20140717020720_27060 | ASTER | Inventory |
| 510 | 10/25/2010 | AST_L1A_00310252010055211_20140717020720_27062 | ASTER | Inventory |
| 511 | 11/3/2010 | AST_L1A_00311032010054630_20140717023710_29589 | ASTER | Inventory |
| 512 | 11/19/2010 | AST_L1A_00311192010054627_20140717020720_27052 | ASTER | Inventory |
| 513 | 11/19/2010 | AST_L1A_00311192010054636_20140717020710_27009 | ASTER | Inventory |
| 514 | 5/5/2011 | AST_L1A_00305052011055239_20140721081755_6258 | ASTER | Inventory |
| 515 | 5/5/2011 | AST_L1A_00305052011055248_20140721081755_6251 | ASTER | Inventory |
| 516 | 5/28/2011 | AST_L1A_00305282011055843_20140721081735_5666 | ASTER | Inventory |
| 517 | 9/10/2011 | AST_L1A_00309102011055206_20140721081735_5681 | ASTER | Inventory |
| 518 | 9/10/2011 | AST_L1A_00309102011055215_20140721081735_5683 | ASTER | Inventory |
| 519 | 10/5/2011 | AST_L1A_00310052011054602_20140721081735_5678 | ASTER | Inventory |
| 520 | 10/12/2011 | AST_L1A_00310122011055225_20140721081805_6372 | ASTER | Inventory |
| 521 | 10/12/2011 | AST_L1A_00310122011055216_20140721081735_5671 | ASTER | Inventory |
| 522 | 11/6/2011 | AST_L1A_00311062011054606_20140721081805_6370 | ASTER | Inventory |
| 523 | 11/6/2011 | AST_L1A_00311062011054615_20140721081805_6371 | ASTER | Inventory |
| 524 | 11/20/2011 | AST_L1A_00311202011055813_20140721081805_6360 | ASTER | Inventory |
| 525 | 11/29/2011 | AST_L1A_00311292011055214_20140721081755_6261 | ASTER | Inventory |
| 526 | 11/29/2011 | AST_L1A_00311292011055223_20140721081755_6263 | ASTER | Inventory |
| 527 | 6/15/2012 | AST_L1A_00306152012055840_20140721081745_6028 | ASTER | Inventory |
| 528 | 8/20/2012 | AST_L1A_00308202012054630_20140721081755_6246 | ASTER | Inventory |
| 529 | 10/7/2012 | AST_L1A_00310072012054613_20140721081745_6043 | ASTER | Inventory |
| 530 | 10/7/2012 | AST_L1A_00310072012054604_20140721081745_6046 | ASTER | Inventory |
| 531 | 10/30/2012 | AST_L1A_00310302012055221_20140721081745_6042 | ASTER | Inventory |
| 532 | 10/30/2012 | AST_L1A_00310302012055230_20140721081745_6037 | ASTER | Inventory |
| 533 | 6/13/2013 | AST_L1A_00306132013054058_20140717024710_6745 | ASTER | Inventory |
| 534 | 6/13/2013 | AST_L1A_00306132013054107_20140717024721_6814 | ASTER | Inventory |
| 535 | 9/6/2013 | AST_L1A_00309062013055913_20140721082654_22887 | ASTER | Inventory |
| 536 | 9/6/2013 | AST_L1A_00309062013055922_20140721082654_22884 | ASTER | Inventory |
| 537 | 9/6/2013 | AST_L1A_00309062013055904_20140721082654_22885 | ASTER | Inventory |
| 538 | 9/8/2013 | AST_L1A_00309082013054659_20140721082714_23067 | ASTER | Inventory |
| 539 | 9/8/2013 | AST_L1A_00309082013054708_20140721082714_23065 | ASTER | Inventory |
| 540 | 9/8/2013 | AST_L1A_00309082013054651_20140721082654_22877 | ASTER | Inventory |
| 541 | 9/17/2013 | AST_L1A_00309172013054103_20140429014124_3777 | ASTER | Inventory |
| 542 | 9/17/2013 | AST_L1A_00309172013054054_20140717024721_6815 | ASTER | Inventory |
| 543 | 9/17/2013 | AST_L1A_00309172013054103_20140717024720_6811 | ASTER | Inventory |
| 544 | 9/26/2013 | AST_L1A_00309262013053455_20140717024710_6750 | ASTER | Inventory |
| 545 | 9/26/2013 | AST_L1A_00309262013053447_20140717024710_6751 | ASTER | Inventory |
| 546 | 9/26/2013 | AST_L1A_00309262013053447_20140429014124_3779 | ASTER | Inventory |
| 547 | 9/26/2013 | AST_L1A_00309262013053455_20140429014124_3771 | ASTER | Inventory |
| 548 | 10/10/2013 | AST_L1A_00310102013054628_20140721082714_23060 | ASTER | Inventory |
| 549 | 10/10/2013 | AST_L1A_00310102013054636_20140721082704_23022 | ASTER | Inventory |
| 550 | 10/10/2013 | AST_L1A_00310102013054645_20140721082704_23021 | ASTER | Inventory |
| 551 | 10/19/2013 | AST_L1A_00310192013054025_20140721082704_23010 | ASTER | Inventory |
| 552 | 10/19/2013 | AST_L1A_00310192013054017_20140717024720_6803 | ASTER | Inventory |
| 553 | 10/19/2013 | AST_L1A_00310192013054017_20140721082704_23014 | ASTER | Inventory |
| 554 | 10/19/2013 | AST_L1A_00310192013054025_20140717024720_6798 | ASTER | Inventory |
| 555 | 10/28/2013 | AST_L1A_00310282013053426_20140429014124_3774 | ASTER | Inventory |
| 556 | 10/28/2013 | AST_L1A_00310282013053426_20140717024710_6748 | ASTER | Inventory |
| 557 | 10/28/2013 | AST_L1A_00310282013053435_20140429014124_3761 | ASTER | Inventory |
| 558 | 10/28/2013 | AST_L1A_00310282013053435_20140717024710_6753 | ASTER | Inventory |
| 559 | 11/18/2013 | AST_L1A_00311182013055240_20140721082704_23005 | ASTER | Inventory |
| 560 | 11/18/2013 | AST_L1A_00311182013055249_20140721082654_22876 | ASTER | Inventory |

Supplementary Table S7 Criteria for identifying surge-type glaciers adopted by studies in the Karakoram and global level.

|  | Study | Region | Criteria |
| --- | --- | --- | --- |
| 1. | Barrand and Murray (2006)^1^ | Karakoram | 1. Direct evidence for surge (s) exist in the literature or where present:   (2) Surface morphological features such as looped or contorted moraines said to be diagnostic of surge behavior.  (3) Other surface features present that are potentially diagnostic of surging, e.g. potholes or surface lakes.  (4) Significant areas of transverse crevassing present, possibly caused by longitudinal extension stresses during an active surge phase.  (5) Pro-glacial features possibly diagnostic of surging, e.g. ‘‘Usherbreen-type’’ concentric moraine circles, mud, or landslides. |
| 2. | Copland et al. (2011)^9^ | Karakoram | 1. Looped/folded medial moraines and surface foliation.  2. Terminus advance in relation to surrounding glaciers.  3. Terminus steepening and thickening.  4. Surface velocities that increase by an order of magnitude or more during a surge, compared to motion during the quiescent phase.  5. Strandlines on valley side-walls in the accumulation area or remnants of glacier surface are left as a rim of ice on. |
| 3. | Bhambri et al. (2013)^3^ | Karakoram | Surge-type glaciers based on folded moraines and heavily crevassed surfaces at the glacier front and/or rapid frontal advances in a short period, visually identified on multi-temporal satellite images. |
| 4. | Hewitt (2014)^24^ | Karakoram | Morphological criteria include ‘bulging moraine loops’, ‘convoluted medial and lateral moraines’, ‘looped’ or ‘teardrop’ medial moraines.  Trim lines in the ablation area, on valley side-walls can contain distinctive lines of polished bedrock and/or disturbed sediment left by rapid reduction in glacier surface elevation after surging. |
| 5. | Rankl et al. (2014)^46^ | Karakoram | Surge glaciers identified by investigating annual termini-position changes using Landsat time series between 1976 and 2012. Also surface velocities, surface features like crevasses, and/or terminus thickening used. |
| 6. | Paul (2015)^65^ | Karakoram | Surging glacier identified based on easily identifiable strong and partly rapid advance or on the basis of its classification in previous studies (Copland et al., 2011^9^; Gardelle et al., 2013^66^; Rankl et al., 2014^46^). Study reported that surging glaciers in the Karakoram are often small (10 km^2^ or less), in contrast to other regions. They can be steep, debris-free, and advance for years-to-decades at relatively low annual rates (about 100 m a^–1^). |
| 7. | Sevestre and Benn (2015)^67^ | Global | 1. Large increase in glacier flow velocities, followed by a period of abnormally slow flow. Velocities during the active phase typically reach at least an order of magnitude higher than during the passive phase, but also surges observed with velocities as low as 150 m a^–1^.  2. Advance of the glacier terminus, sometimes sudden and dramatic, out of synchrony with the behavior of neighboring glaciers.  3. Surge-type glaciers display a combination of features indicative of oscillations in flow: looped moraines, heavy surface crevassing, push moraines, eskers, etc. |

Supplementary Table S8 Details for satellite image pairs used for automated surge glacier surface feature tracking with the estimated uncertainty.

| **S.No**. | **Glacier** | **Satellite image pairs dates matched** | **Temporal separation (days)** | **Sensors** | **Pixel resolution (m)** | **Estimated uncertainty (m/yr)** |
| --- | --- | --- | --- | --- | --- | --- |
| 1 | Central Rimo | 1992/07/29-1993/09/02 | 400 | TM-TM | 30 | 14 |
| 2 |  | 1993/09/02-1994/10/23 | 416 | TM-TM | 30 | 13 |
| 3 |  | 1994/10/23-1996/09/10 | 688 | TM-TM | 30 | 8 |
| 4 |  | 1996/09/10-1997/07/18 | 311 | TM-TM | 30 | 18 |
| 5 |  | 1997/07/18-1998/09/07 | 416 | TM-TM | 30 | 13 |
| 6 |  | 1998/09/07-1999/07/16 | 312 | TM-ETM+ | 30 | 18 |
| 7 |  | 1999/07/16-2000/08/28 | 409 | ETM+-ETM+ | 30 | 13 |
| 8 | Braldu | 2009/08/04-2010/08/23 | 384 | TM-TM | 30 | 14 |
| 9 |  | 2010/08/23-2011/08/10 | 352 | TM-TM | 30 | 16 |
| 10 |  | 2013/07/30-2013/10/09 | 71 | OLI-OLI | 15 | 39 |
| 11 |  | 2013/09/07-2014/07/24 | 320 | OLI-OLI | 15 | 9 |
| 12 |  | 2014/07/24-2014/08/25 | 32 | OLI-OLI | 15 | 86 |
| 13 |  | 2014/08/25-2015/07/04 | 313 | OLI-OLI | 15 | 9 |
| 14 |  | 2015/07/04-2015/09/13 | 71 | OLI-OLI | 15 | 39 |
| 15 | Kyagar | 1993/07/07-1994/06/24 | 352 | TM-TM | 30 | 16 |
| 16 |  | 1994/06/24-1996/09/01 | 800 | TM-TM | 30 | 7 |
| 17 |  | 1996/09/01-1997/07/18 | 320 | TM-TM | 30 | 17 |
| 18 |  | 1997/07/18-1998/09/07 | 416 | TM-TM | 30 | 13 |
| 19 | North Gasherbrum | 2003/10/15-2004/08/14 | 304 | ETM+-ETM+ | 30 | 18 |
| 20 |  | 2004/08/14-2005/09/02 | 384 | ETM+-ETM+ | 30 | 14 |
| 21 |  | 2005/09/02-2006/08/20 | 352 | ETM+-ETM+ | 30 | 16 |
| 22 |  | 2006/08/20-2007/08/23 | 368 | ETM+-ETM+ | 30 | 15 |
| 23 |  | 2007/08/23-2008/06/06 | 288 | ETM+-ETM+ | 30 | 19 |
| 24 |  | 2008/06/06-2009/08/04 | 424 | ETM+-TM | 30 | 13 |
| 25 |  | 2009/08/04-2010/08/23 | 384 | TM-TM | 30 | 14 |
| 26 | North Terong | 1989/10/09-1990/06/29 | 263 | TM-TM | 30 | 21 |
| 27 |  | 1990/06/29-1991/08/28 | 425 | TM-TM | 30 | 13 |
| 28 |  | 1991/08/28-1992/07/29 | 336 | TM-TM | 30 | 16 |
| 29 |  | 1992/07/29-1993/09/02 | 400 | TM-TM | 30 | 14 |
| 30 |  | 1993/09/02-1994/10/23 | 416 | TM-TM | 30 | 13 |
| 31 |  | 1994/10/23-1996/09/10 | 688 | TM-TM | 30 | 8 |
| 32 |  | 1996/09/10-1997/07/18 | 311 | TM-TM | 30 | 18 |
| 33 |  | 1997/07/18-1998/09/16 | 425 | TM-TM | 30 | 13 |
| 34 |  | 1998/09/16-1999/07/16 | 303 | TM-ETM+ | 30 | 18 |
| 35 |  | 1999/07/16-2000/08/28 | 409 | ETM+-ETM+ | 30 | 13 |
| 36 |  | 2000/08/28-2001/07/21 | 327 | ETM+-ETM+ | 30 | 17 |
| 37 |  | 2001/07/21-2002/08/02 | 377 | ETM+-ETM+ | 30 | 15 |
| 38 | Siachen | 1989/08/06-1990/06/29 | 328 | TM-TM | 30 | 17 |
| 39 |  | 1990/06/29-1991/08/28 | 426 | TM-TM | 30 | 13 |
| 40 |  | 1991/08/28-1992/11/25 | 456 | TM-TM | 30 | 12 |
| 41 |  | 1992/11/25-1993/07/07 | 225 | TM-TM | 30 | 24 |
| 42 |  | 1993/07/07-1994/06/24 | 353 | TM-TM | 30 | 16 |
| 43 |  | 1994/06/24-1995/11/11 | 506 | TM-TM | 30 | 11 |
| 44 |  | 1995/11/11-1996/07/31 | 264 | TM-TM | 30 | 21 |
| 45 |  | 1996/07/31-1997/07/18 | 353 | TM-TM | 30 | 16 |
| 46 |  | 1997/07/18-1998/09/07 | 447 | TM-TM | 30 | 12 |
| 47 |  | 1998/09/07-1999/10/29 | 418 | TM-ETM+ | 30 | 13 |
| 48 |  | 1999/10/29-2000/08/28 | 305 | ETM+-ETM+ | 30 | 18 |
| 49 |  | 2000/08/28-2001/06/19 | 296 | ETM+-ETM+ | 30 | 18 |
| 50 |  | 2001/06/19-2002/10/12 | 481 | ETM+-ETM+ | 30 | 11 |
| 51 | South Rimo | 1989/10/09-1991/08/28 | 688 | TM-TM | 30 | 8 |
| 52 |  | 1991/08/28-1992/07/29 | 336 | TM-TM | 30 | 16 |
| 53 |  | 1992/07/29-1993/09/02 | 400 | TM-TM | 30 | 14 |
| 54 |  | 1993/09/02-1994/10/23 | 416 | TM-TM | 30 | 13 |
| 55 | Staghar | 1989/11/17-1989/12/03 | 17 | TM-TM | 30 | 322 |
| 56 |  | 1989/12/03-1990/03/09 | 96 | TM-TM | 30 | 57 |
| 57 |  | 1991/08/19-1992/06/02 | 288 | TM-TM | 30 | 19 |
| 58 |  | 1993/07/07-1994/06/24 | 352 | TM-TM | 30 | 16 |
| 59 |  | 2008/06/14-2009/08/04 | 416 | TM-TM | 30 | 13 |
| 60 |  | 2009/08/04-2010/08/23 | 384 | TM-TM | 30 | 14 |
| 61 |  | 2010/08/23-2011/08/10 | 352 | TM-TM | 30 | 16 |
| 62 |  | 2011/08/10-2013/07/14 | 704 | TM-TM | 30 | 8 |
| 63 | Urdok | 1991/08/19-1992/06/02 | 288 | TM-TM | 30 | 19 |
| 64 |  | 1992/06/02-1993/07/07 | 400 | TM-TM | 30 | 14 |
| 65 |  | 1993/07/07-1994/06/24 | 352 | TM-TM | 30 | 16 |
| 66 |  | 1994/06/24-1995/11/21 | 515 | TM-TM | 30 | 11 |
| 67 |  | 1996/09/01-1997/07/18 | 320 | TM-TM | 30 | 17 |
| 68 |  | 1997/07/18-1998/09/07 | 416 | TM-TM | 30 | 13 |
| 69 | Yazghil | 1989/02/09-1990/08/07 | 554 | TM-TM | 30 | 10 |
| 70 |  | 1990/08/07-1992/10/15 | 800 | TM-TM | 30 | 7 |
| 71 |  | 1992/11/16-1993/06/12 | 208 | TM-TM | 30 | 26 |
| 72 |  | 1993/06/12-1994/07/01 | 384 | TM-TM | 30 | 14 |
| 73 |  | 1994/07/01-1996/07/06 | 736 | TM-TM | 30 | 7 |
| 74 |  | 1996/07/06-1997/08/10 | 400 | TM-TM | 30 | 14 |
| 75 |  | 1997/08/10-1998/08/13 | 368 | TM-TM | 30 | 15 |
| 76 |  | 1998/08/13-1999/08/16 | 368 | TM-TM | 30 | 15 |
| 77 |  | 1999/08/16-2000/09/11 | 392 | TM-ETM+ | 30 | 14 |
| 78 |  | 2000/09/11-2001/08/29 | 352 | ETM+-ETM+ | 30 | 16 |
| 79 |  | 2001/08/29-2002/08/16 | 352 | ETM+-ETM+ | 30 | 16 |
| 80 |  | 2002/08/16-2003/09/20 | 400 | ETM+-ETM+ | 30 | 14 |
| 81 |  | 2003/09/20-2004/08/05 | 320 | ETM+-ETM+ | 30 | 17 |
| 82 |  | 2004/08/05-2005/06/21 | 320 | ETM+-ETM+ | 30 | 17 |
| 83 |  | 2005/06/21-2006/08/27 | 432 | ETM+-ETM+ | 30 | 13 |
| 84 |  | 2006/08/27-2007/09/15 | 384 | ETM+-ETM+ | 30 | 14 |
| 85 |  | 2007/09/15-2008/07/31 | 320 | ETM+-ETM+ | 30 | 17 |
| 86 |  | 2008/07/31-2009/08/11 | 376 | ETM+-TM | 30 | 15 |
| 87 |  | 2009/08/11-2010/10/17 | 432 | TM-TM | 30 | 13 |
| 88 |  | 2010/10/17-2011/06/22 | 248 | TM-ETM+ | 30 | 22 |
| 89 |  | 2011/06/22-2012/09/12 | 448 | ETM+-ETM+ | 30 | 12 |
| 90 |  | 2012/09/12-2013/10/09 | 392 | ETM+-OLI | 30 | 14 |
| 91 |  | 2013/10/09-2014/08/25 | 320 | OLI-OLI | 15 | 9 |
| 92 |  | 2014/08/25-2015/09/13 | 384 | OLI-OLI | 15 | 7 |
| 93 | Batura | 1990/08/07-1992/10/15 | 800 | TM-TM | 30 | 7 |
| 94 |  | 1992/10/15-1993/06/12 | 240 | TM-TM | 30 | 23 |
| 95 |  | 1993/06/12-1994/07/17 | 400 | TM-TM | 30 | 14 |
| 96 |  | 1994/09/03-1996/07/06 | 672 | TM-TM | 30 | 8 |
| 97 |  | 1996/07/06-1997/08/10 | 400 | TM-TM | 30 | 14 |
| 98 |  | 1997/08/10-1998/08/13 | 368 | TM-TM | 30 | 15 |
| 99 |  | 1998/08/29-1999/09/16 | 383 | TM-ETM+ | 30 | 14 |
| 100 |  | 1999/09/16-2000/09/11 | 361 | ETM+-ETM+ | 30 | 15 |
| 101 |  | 2000/09/11-2001/08/29 | 352 | ETM+-ETM+ | 30 | 16 |
| 102 |  | 2001/08/29-2002/07/15 | 320 | ETM+-ETM+ | 30 | 17 |
| 103 |  | 2002/07/15-2003/05/31 | 320 | ETM+-ETM+ | 30 | 17 |
| 104 |  | 2009/08/27-2010/11/02 | 432 | TM-TM | 30 | 13 |
| 105 |  | 2010/11/02-2011/05/29 | 208 | TM-TM | 30 | 26 |
| 106 |  | 2011/05/29-2013/10/09 | 864 | TM-OLI | 30 | 6 |
| 107 | Ghulkin | 1990/08/07-1992/10/15 | 800 | TM-TM | 30 | 7 |
| 108 |  | 1992/10/15-1993/06/12 | 240 | TM-TM | 30 | 23 |
| 109 |  | 1993/06/12-1994/07/17 | 400 | TM-TM | 30 | 14 |
| 110 |  | 1994/09/03-1996/07/06 | 672 | TM-TM | 30 | 8 |
| 111 |  | 1996/07/06-1997/08/10 | 400 | TM-TM | 30 | 14 |
| 112 |  | 1997/08/10-1998/08/13 | 368 | TM-TM | 30 | 15 |
| 113 |  | 1998/08/29-1999/09/16 | 383 | TM-ETM+ | 30 | 14 |
| 114 |  | 1999/09/16-2000/09/11 | 361 | ETM+-ETM+ | 30 | 15 |
| 115 |  | 2000/09/11-2001/08/29 | 352 | ETM+-ETM+ | 30 | 16 |
| 116 |  | 2001/08/29-2002/07/15 | 320 | ETM+-ETM+ | 30 | 17 |
| 117 |  | 2002/07/15-2003/05/31 | 320 | ETM+-ETM+ | 30 | 17 |
| 118 |  | 2009/08/27-2010/11/02 | 432 | TM-TM | 30 | 13 |
| 119 |  | 2010/11/02-2011/05/29 | 208 | TM-TM | 30 | 26 |
| 120 |  | 2011/05/29-2013/10/09 | 864 | TM-OLI | 30 | 6 |

**3. Supplementary Figures**


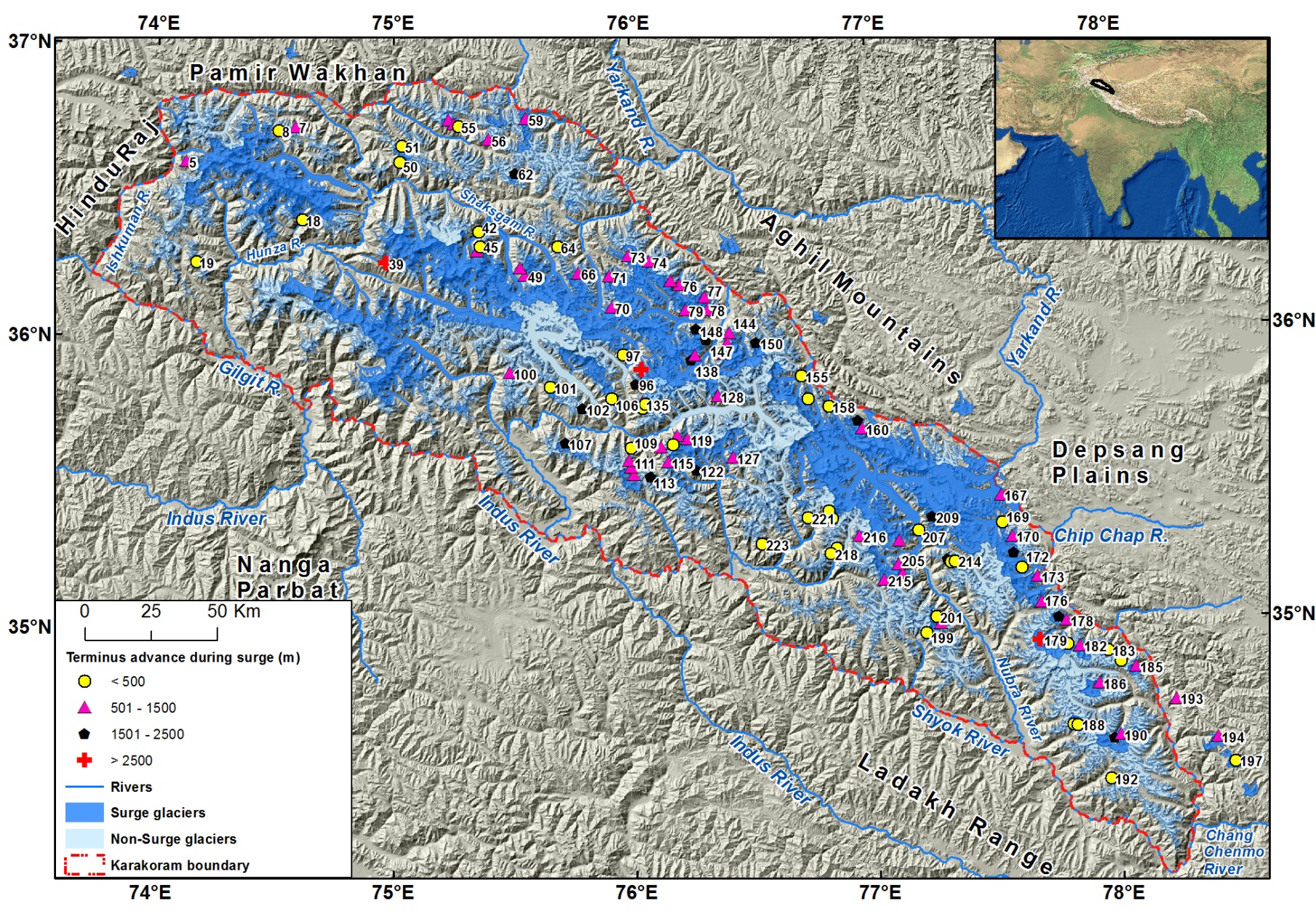


Supplementary Fig. S1 Glacier terminus advancement during surge for 111 glaciers in the Karakoram and Chang Chenmo region. The map is generated by the open-source datasets using ArcGIS version 10.0 (http://www.esri.com/software/arcgis/arcgis-for-desktop). The hill-shaded background in this figure have been generated by using the Shuttle Radar Topography Mission (SRTM) data set (90 × 90 m) provided by the CGIAR Consortium for Spatial Information ([http://srtm.csi.cgiar.org/)](http://srtm.csi.cgiar.org/%29). The glacier outlines were modified (details has given in methods) from the Randolph Glacier Inventory (RGI 5.0) (Pfeffer et al. 2014)^69^ (http://www.glims.org/RGI/00_rgi50_TechnicalNote.pdf). Terminus advancement of surge-type glacier is presented in Supplementary Table S1.





Supplementary Fig. S2 Surface flow displacement of four Karakoram surge glaciers [(a) Urdok (ID 157); (b) Kyagar (ID 163); (c) Central Rimo (ID 167), and (d) South Rimo (ID 169)] based on Image correlation software (CIAS) (Kääb and Vollmer, 2000)^68^ and Heid and Kääb (2012)^70^ (<http://www.mn.uio.no/geo/english/research/projects/icemass/cias/cias.sav>). CIAS was originally written by M. Vollmer and A. Kääb (Kääb and Vollmer, 2000)^68^ and further developed by A. Kääb. Satellite image pairs used for automated feature tracking including the estimated uncertainty presented in Supplementary Table S8. Location of surface displacement of surge glaciers in the Karakoram is presented in Supplementary Fig. S5.





Supplementary Fig. S3 Surface flow displacement of four Karakoram surge glaciers [(a), (b): Siachen (ID 207); (c), (d): Ghulkin (ID 13); (e), (f) Batura (ID 11), and (g), (h) North Terong Glacier (ID 211)] based on Image correlation software (CIAS) (Kääb and Vollmer, 2000)^68^ and Heid and Kääb (2012)^70^ (<http://www.mn.uio.no/geo/english/research/projects/icemass/cias/cias.sav>). CIAS was originally written by M. Vollmer and A. Kääb (Kääb and Vollmer, 2000)^68^ and further developed by A. Kääb. Satellite image pairs used for automated feature tracking including the estimated uncertainty presented in Supplementary Table S8. Location of surface displacement of surge glaciers in the Karakoram is presented in Supplementary Fig. S5.





Supplementary Fig. S4 Manually measured glacier surface displacement: (a) very slow glacier surface flow (< 100 m/y), (b) medium glacier surface flow (100 -500 m/y), and (c) fast glacier surface flow (> 500 m/y).


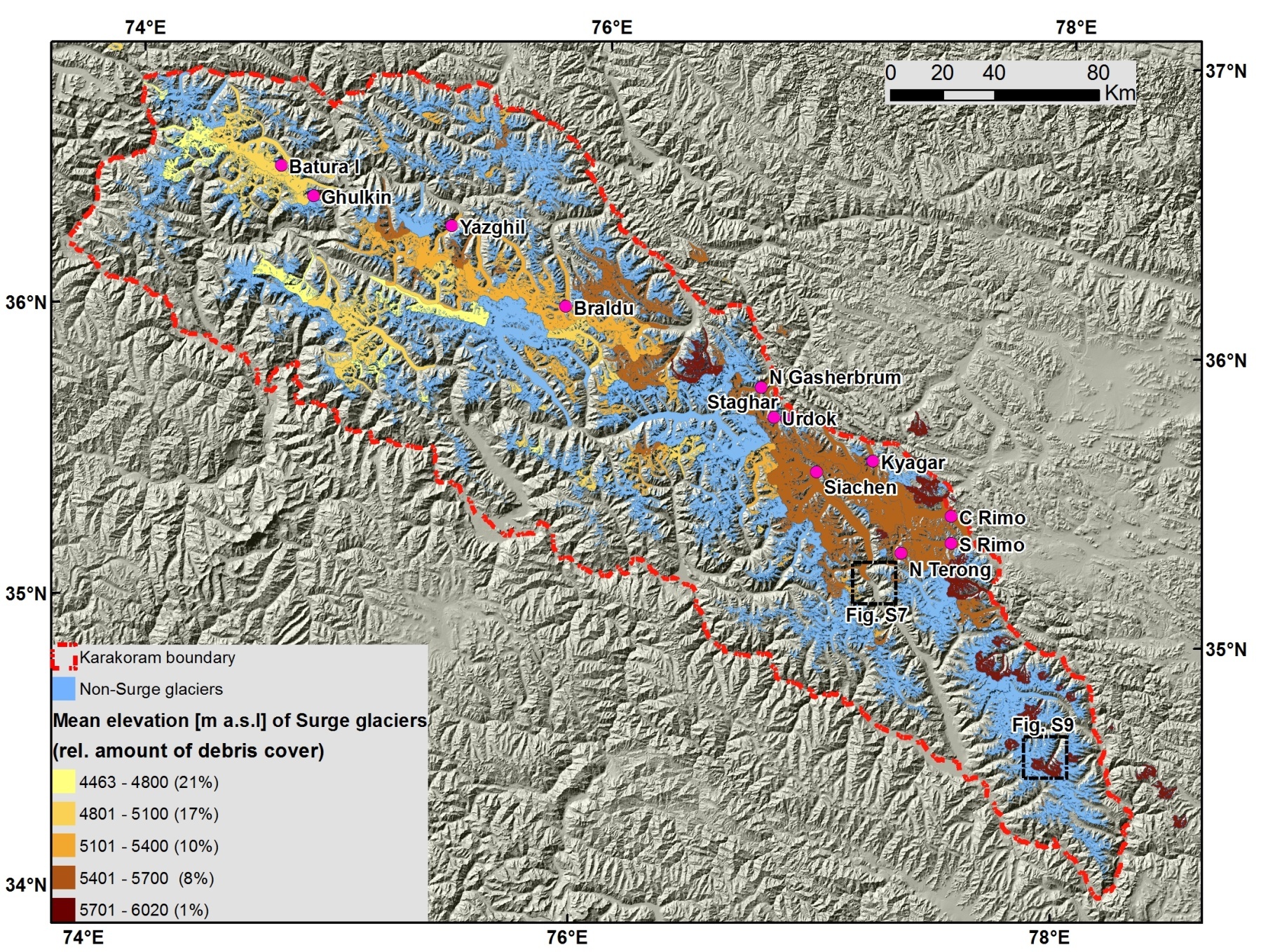


Supplementary Fig. S5 Mean glacier elevation (m a.s.l) and relative amount of debris cover (%) is spatially correlated: Mean elevation is increasing from south to northeast, while the debris cover (presented by the percentage in brackets) is decreasing along this gradient. The map is generated by the open-source datasets using ArcGIS version 10.0 (http://www.esri.com/software/arcgis/arcgis-for-desktop). The hill-shaded background in this figure have been generated by using the Shuttle Radar Topography Mission (SRTM) data set (90 × 90 m) provided by the CGIAR Consortium for Spatial Information ([http://srtm.csi.cgiar.org/)](http://srtm.csi.cgiar.org/%29). The glacier outlines were modified (details has given in methods) from the Randolph Glacier Inventory (RGI 5.0) (Pfeffer et al. 2014)^69^ (http://www.glims.org/RGI/00_rgi50_TechnicalNote.pdf). Pink circles shows locations of surge-type glaciers which are selected for surface displacement using Image correlation software (CIAS) (Kääb and Vollmer, 2000)^68^ and Heid and Kääb (2012)^70^ (<http://www.mn.uio.no/geo/english/research/projects/icemass/cias/cias.sav>). CIAS was originally written by M. Vollmer and A. Kääb (Kääb and Vollmer, 2000)^68^ and further developed by A. Kääb. Surface displacement of these glaciers is presented in Supplementary Fig. S2, S3 and main text Fig. 3.





Supplementary Fig. S6 Cumulative length change of surge-type glaciers during the repeated surge. Glacier ID and other details (e.g. latitude and longitude) are presented in Supplementary Table S1. Blue line present the initial position of the glaciers measured from the remote sensing data.


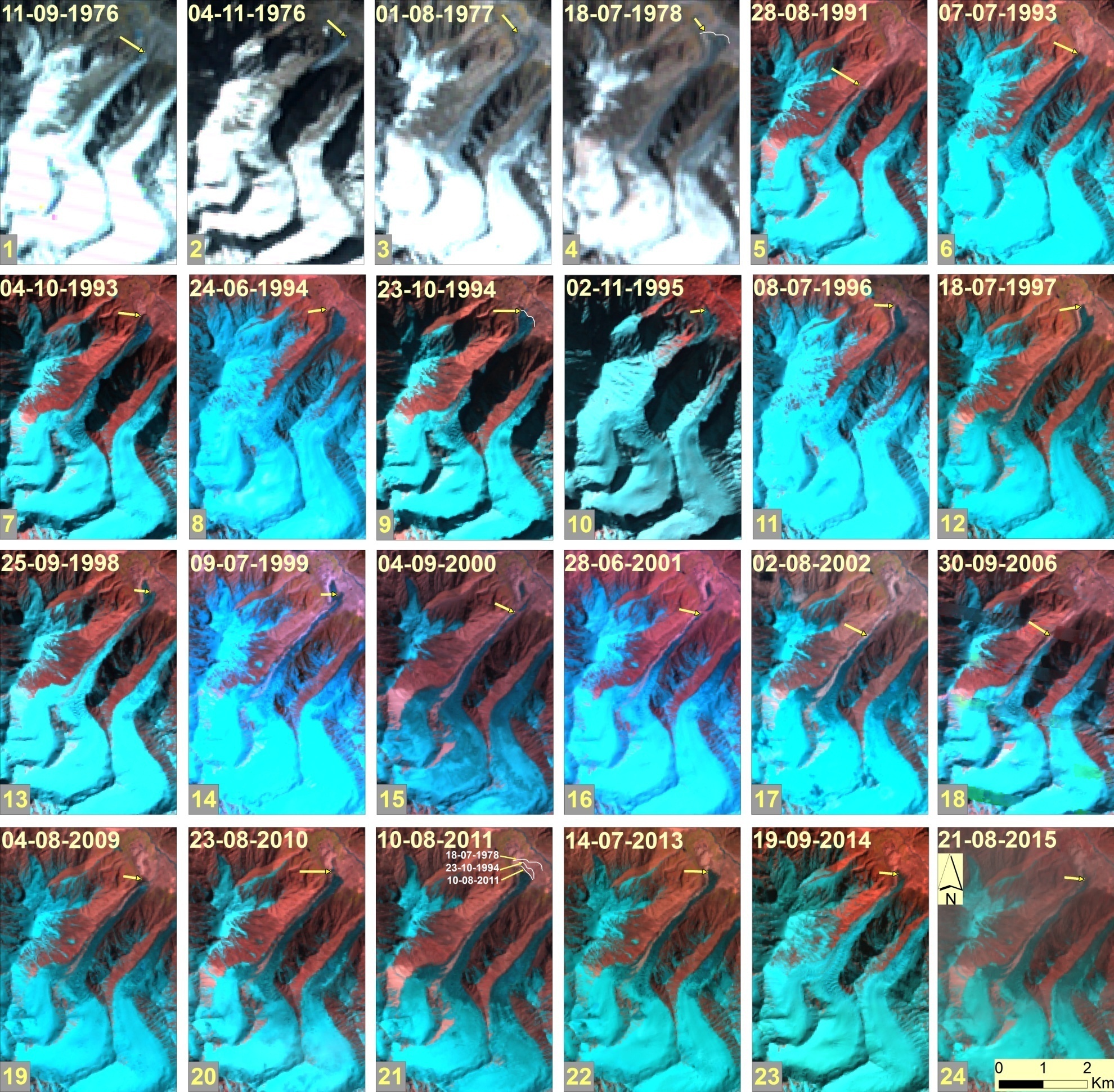


Supplementary Fig. S7 One unnamed glacier (ID 212 in Supplementary Table S1) near the North Terong Glacier (ID 211) surged three times from 1976 to 2015 (1976-1978; 1991-1994; 2006-2011). Maximum extent of three surges during 1978, 1994 and 2011 is presented in sub figure 21. Location of terminus is presented by yellow arrows in the sub figures. Sub figures 1-4 presented by Landsat MSS (cell size 79 m) and Sub figures 5-24 presented by Landsat TM, ETM+ and OLI (cell size 30 m). Location of unnamed glacier (ID 212) in the Karakoram is presented in Supplementary Fig. S5. Landsat TM, ETM+ and OLI data are courtesy of the U.S. Geological Survey (<http://earthexplorer.usgs.gov>).





Supplementary Fig. S8 Cumulative length change of selected surge-type glaciers in the Karakoram since 1880 based on Mason (1930)^35^, Goudie et al. (1984)^17^ and present study. Location of glaciers (ID) in the Karakoram is presented in Fig. 1 of main text.


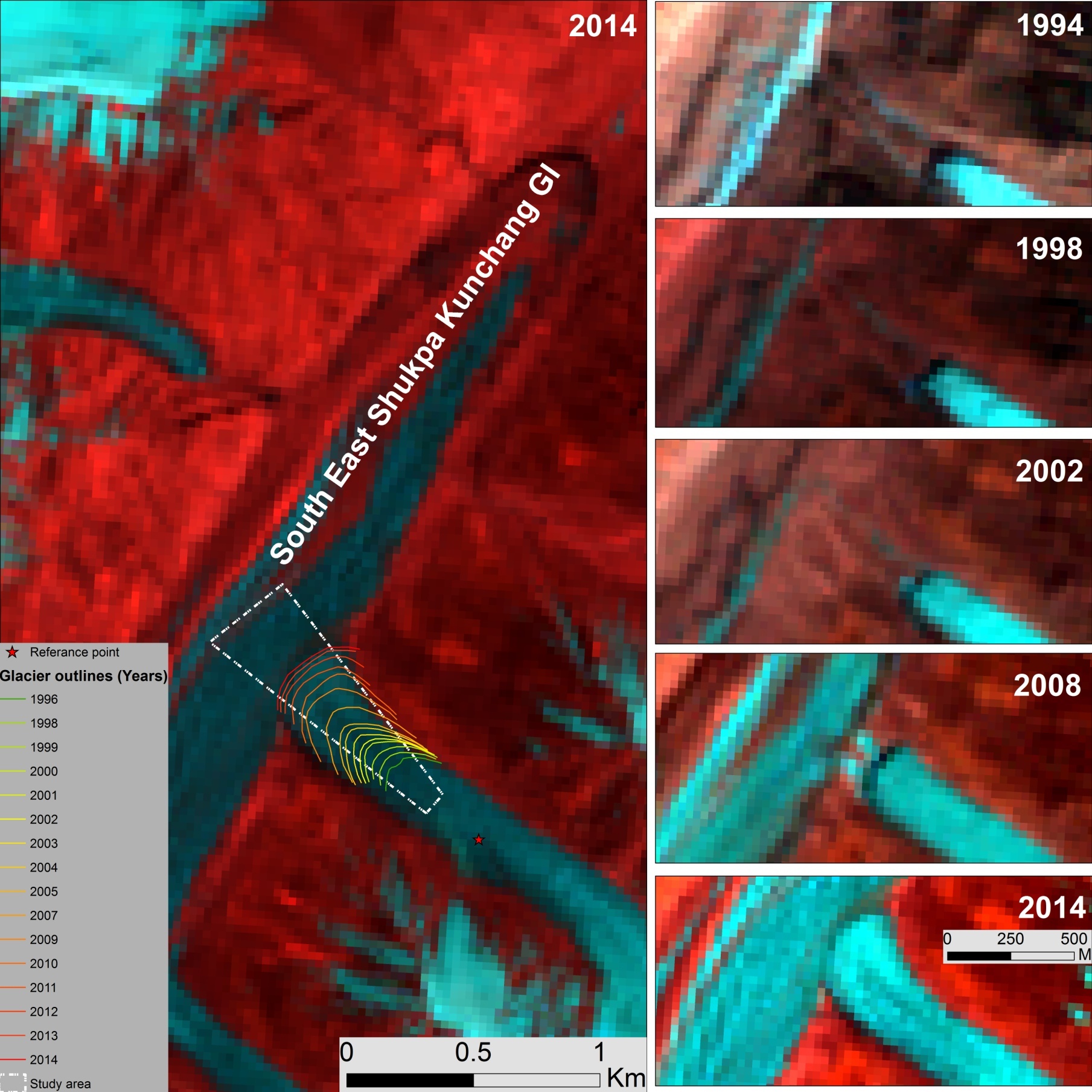


Supplementary Fig. S9 Left part of figure is showing frontal length measurement of unnamed tributary (ID 190 Supplementary Table S1) of South East Shukpa Kunchang Glacier (ID 189 Supplementary Table S1) using a glacier length tool developed by previous study (Bjørk et al. 2012)^6^ based on ArcGIS 10.0 software (http://www.esri.com/software/arcgis/arcgis-for-desktop). This tool divides the glacier front into points spaced (i.e. 15m apart) and calculates the mean distance to a reference point (red star) on the glacier. Study area (white colour box) limits the glacier outlines to calculate the mean distance to a reference point (red star). Using the same reference point for all years enables a direct comparison of changes in front position. Right part of figure is showing progress of glacier surge by temporal Landsat TM, ETM+ and OLI satellite images (cell size 30 m) (years 1994, 1998, 2002, 2008 and 2014). Landsat TM, ETM+ and OLI data are courtesy of the U.S. Geological Survey (<http://earthexplorer.usgs.gov>). See Supplementary Fig. S5 for location of unnamed tributary of South East Shukpa Kunchang Glacier in the Karakoram.

**References**

1. Barrand, N. & Murray, T. Multivariate controls on the incidence of glacier surging in the Karakoram Himalaya. *Arct. Antarct. Alp. Res.* **38**, 489-498 (2006).
2. Belo`, M., Mayer, C., Smiraglia, C. & Tamburini, A. The recent evolution of Liligo Glacier, Karakoram, Pakistan, and its present quiescent phase. *Ann. Glaciol.* **48,** 171–176 (2008).
3. Bhambri, R., Bolch, T., Kawishwar, P., Dobhal, D. P., Srivastava, D. & Pratap, B. Heterogeneity in glacier response in the upper Shyok valley, northeast Karakoram. *Cryosphere* **7**, 1385-1398 (2013).
4. Bhutiyani, M. R. Kale, V.S. & Pawar, N. J. Climate change and the precipitation variations in the northwestern Himalaya: 1866-2006. *Int. J. Climatol.* **30**, 535-548 (2010).
5. Bhutiyani, M. R. Kale, V.S. & Pawar, N. J. Long-term trends in maximum, minimum and mean annual air temperatures across the Northwestern Himalaya during the twentieth century. *Clim. Chang.* **85**, 159-177 (2007).
6. Bjørk, A. A. *et al.* An aerial view of 80 years of climate-related glacier fluctuations in southeast Greenland. *Nat. Geosci.* **5**, 427-432 (2012).
7. Bocchiola, D. & Diolaiuti, G. 2013. Recent (1980–2009) evidence of climate change in the upper Karakoram, Pakistan. *Theor. Appl. Climatol.* **113**, 611-641 (2013).
8. Conway, W. M. Climbing and Exploration in the Karakoram Himalaya. London: Unwin, 3 vols (1894).
9. Copland, L., Sharp, M.J. & Dowdeswell, J.A. The distribution and flow characteristics of surge-type glaciers in the Canadian High Arctic. *Ann. Glaciol.* **36**, 73-81 (2003).
10. Desio, A. An exceptional glacier advance in the Karakoram-Ladakh region. *J. Glaciol*. **2**, 383-385 (1954).
11. Desio, A. Geological work of the Italian Expedition to the Karakorum. *Geogr. J.* **75**, 402-411 (1930).
12. Desio, A., Marussi, A. & Caputo, M. Glaciological research of the Italian Karakorum Expedition 1953–1955. *IASH Publication*. **52**, 224-232 (1961).
13. Dimri, A.P. & Dash, S.K. Winter temperature and precipitation trends in the Siachen Glacier. *Curr. Sci.* **98**, 1620-1624 (2010).
14. Diolaiuti, G., Pecci, M. & Smiraglia, C. Liligo Glacier, Karakoram, Pakistan: a reconstruction of the recent history of a surge-type glacier. *Ann. Glaciol.* **36**, 168-172 (2003).
15. Fowler, H. J. & Archer, D.R. Conflicting signals of climatic change in the Upper Indus Basin. *J. Clim*. **19**, 4276-4293 (2006).
16. Gardner, J.S. & Hewitt, K. Surge of the Bualtar Glacier, Karakoram Ranges, Pakistan: a possible landslide trigger. *J. Glaciol*. **36**, 159-162 (1990).
17. Goudie, A.S., Jones, D.K.C. & Brunsden, D. Recent fluctuations in some glaciers of the Western Karakoram mountains, Hunza, Pakistan. *The international Karakoram project*, **2**, 411-455 (1984).
18. Godwin-Austen, H. H. The glaciers of the Muztagh Range. *Proceedings of the Royal Geographic Society*. **34**, 19-56 (1864).
19. Hasson, S., Böhner, J. & Lucarini, V. Prevailing climatic trends and runoff response from Hindukush–Karakoram–Himalaya, upper Indus basin. *Earth Syst. Dynam. Discuss.* **6**, 579-653 (2015).
20. Hayden, H. H. Notes on certain glaciers in Northwest Kashmir. *Records of the Geological Survey of India*. **35**, 127-137 (1907).
21. Hayward, G. W. Journey from Leh to Yarkand and Kashgar, and Exploration of the Sources of the Yarkand River. *The Journal of the Royal Geographical Society of London*. **40**, 33-166 (1870).
22. Hewitt, K. & Liu, J. Ice-dammed lakes and outburst floods, Karakoram Himalaya: Historical perspectives and emerging threats. *Phys. Geogr.* **31**, 528–551 (2010).
23. Hewitt, K. Glacier surges in the Karakoram Himalaya, Central Asia. *Can. J. Earth Sci.* **6**, 1009-1018 (1969).
24. Hewitt, K. Glaciers of the Karakoram Himalaya: Glacial Environments, Processes, Hazards and Resources. Springer Science & Business Media (2014).
25. Hewitt, K. Glaciers receive a surge of attention in the Karakoram Himalaya. *EOS, Trans. Am. Geophys. Union* **79**, 104-105 (1998).
26. Hewitt, K. The Karakoram anomaly? Glacier expansion and the “elevation effect”, Karakoram Himalaya. *Mt. Res. Dev.* **25**, 332–340 (2005).
27. Hewitt, K. Tributary glacier surges: an exceptional concentration at Panmah Glacier, Karakoram Himalaya. *J. Glaciol*. **53**, 181-188 (2007).
28. Iturrizaga, L. New observations on present and prehistorical glacier-dammed lakes in the Shimshal valley (Karakoram Mountains). *J. Asian Earth Sci*. **25**, 545–555 (2005).
29. Kick, W. Exceptional glacier advances in the Karakoram. *J. Glaciol.* **3**, 229 (1958).
30. Kreutzmann, H. Habitat conditions and settlement processes in the Hindukush-Karakoram. *Petermanns Geographische Mitteilungen.* **138**, 337-356 (1994).
31. Longstaff, T. G. Glacier exploration in the Eastern Karakoram. *Geogr. J.* **35**, 622-658 (1910).
32. Lyall-Grant, I. H. & Mason, K. The Upper Shyok Glaciers in 1939. *The Himalayan Journal*. **12**, 52–63 (1940).
33. Mason, K. Discussion, p. 426, of Karakoram 1939 by E. Shipton. *Geogr. J.* **95**, 409-427 (1940).
34. Mason, K. Expedition notes: tours of the Gilgit Agency. *The Himalayan Journal*. **3**, 110-115 (1931).
35. Mason, K. The glaciers of the Karakoram and neighbourhood. *Records of Geological Survey of India* **63**, 214–278, (1930).
36. Mason. K. The study of the threatening glaciers. *Geogr. J.* **85**, 24-41 (1935).
37. Mayer, C., Fowler, A.C., Lambrecht, A. & Scharrer, K. A surge of North Gasherbrum Glacier, Karakoram, China. *J. Glaciol.* **57**, 904-916 (2011).
38. Mayewski, P. A. & Jeschke, P. A. Himalayan and trans-Himalayan glacier fluctuations since A.D. 1812. *Arct. Alp. Res.* **11**, 267-287 (1979).
39. Meiners, S. The glacial history of landscape in the Batura Muztagh, NW Karakoram. *GeoJournal* **63**, 49-90 (2005).
40. Neve, A. Rapid glacial advance in the Hindu Kush. *Alp. J*. **23**, 400-401 (1907).
41. Paffen, K. H., Pillewizer, W. & Schneider, H. J. Forschungen im Hunza-Karakorum: Vorla¨ufiger Bericht u¨ ber die Wissenschaftlichen Arbeiten der Deutsch-O¨ sterreichischen Himalaya-Karakorum-Expedition, 1954. *Erdkunde* **10**, 15-28 (1956).
42. Pecci, M. & Smiraglia, C. Advance and retreat phases of the Karakorum glaciers during the 20th century: case studies in Braldo Valley (Pakistan). *Geogr. Fis. Dinam. Quat.* **23**, 73-85 (2000).
43. Quincey, D. & Luckman, A. Brief Communication: On the magnitude and frequency of Khurdopin glacier surge events. *Cryosphere* **8**, 571-574 (2014).
44. Quincey, D. J., Glasser, N. F., Cook, S. J. & Luckman A. Heterogeneity in Karakoram glacier surges. *J. Geophys. Res. Earth Surf.* **120**, 1288–1300 (2015).
45. Quincey, D.J., Braun, M., Glasser, N.F., Bishop, M.P., Hewitt, K. & Luckman, A. Karakoram glacier surge dynamics. *Geophys. Res. Lett.* **38** (2011).
46. Rankl, M., Kienholz, C. & Braun, M. Glacier changes in the Karakoram region mapped by multimission satellite imagery. *Cryosphere* **8**, 977-989, (2014).
47. Richardson, S. D. & Quincey, D. J. Glacier outburst floods from Ghulkin Glacier, Upper Hunza Valley, Pakistan. In EGU General Assembly Conference Abstracts **11**, 12871 (2009).
48. Schlagintweit, H., Schlagintweit, A. & Schlagintweit, R. Results of a Scientific Mission to India and High Asia Undertaken between the Years 1854 and 1858. London: Trubner, 4 vols (1861-1866).
49. Schomberg, R. C. F. The Bar and Daintar Glaciers. *Alp. J.* **46,** 131-142 (1933).
50. Shaw, R. B. Visits to High Tartary, Yaˆrkand, and Kaˆshgar (formerly Chinese Tartary), and Return Journey over the Karakoram Pass. London: John Murray (1871).
51. Shipton, E., Spender, M. & Auden, J.B. The Shaksgam Expedition, 1937. *Geogr. J.* **91**, 313-336 (1938).
52. Shroder, J. & Bishop, M. Glaciers of Pakistan. in *Satellite image atlas of glaciers: Asia*. (ed. Williams, R. S. & Ferrigno, J. G. Jr) 201-252 (United States Geological Survey 2010).
53. Sinclair, M. C. The glaciers of the Upper Shyok in 1928. *Geogr. J.* **74**, 383-387 (1929).
54. Visser, Ph. C. & Visser-Hooft, J. Wissenschaftliche Ergebnisse der niederländischen Expeditionen in den Karakorum und die angrenzenden Gebiete in den Jahren 1922, 1925 und 1929–30. E.J. Brill, Leiden (1935–1938).
55. Wake, C. P. & Searle, M. P. Correspondence. Rapid advance of Pumarikish Glacier, Hispar Glacier basin, Karakoram Himalaya. *J. Glaciol.* **39,** 204–206 (1993).
56. Wang, W., Maohuan, H. & Jinming, C. A surging advance of Balt Bare glacier, Karakoram Mountains. The International Karakoram Project **1**, 76-83 (1984).
57. Workman, W. H. The tongue of the Hassanabad Glacier in 1908. *Geogr. J.* **36**, 194-196 (1910).
58. Workman, W.H. & Workman, F.B. The call of the snowy Hispar: a narrative of exploration and mountaineering on the northern frontier of India. Constable and Company, Ltd. (1911).
59. Youghusband, F. E. "Journeys in the Pamirs and Adjacent countries." Proceedings of the Royal Geographical Society and Monthly Record of Geography. **14**, 205-234 (1892).
60. Younghusband, F. E. The Heart of a Continent. London: John Murray (1896).
61. Zhang, X. Recent variations of some glaciers in the Karakoram Mountains. *The international Karakoram project* **1**, 38-50 (1984).
62. Jiskoot, H. Glacier surging, in Encyclopaedia of Snow, Ice and Glaciers. (ed. Singh, V. P., Singh, P. & Haritashya, U. K.) 415–428 (Springer Heidelberg, 2011).
63. BIG (Batura Investigations Group) (1979) The Batura Glacier in the Karakoram mountains and its variations. Sci Sin 22(8):958–974.
64. Deline, P., Hewitt, K., Reznichenko, N. & Shugar, D. in *Landslide Hazards, Risks, and Disasters*. (ed. Davies, T.) 263–320 (Elsevier, 2014).
65. Paul, F. Revealing glacier flow and surge dynamics from animated satellite image sequences: examples from the Karakoram. Cryosphere 9, 2201-2214 (2015).
66. Gardelle, J., Berthier, E., Arnaud, Y. & Kääb, A. Region-wide glacier mass balances over the Pamir-Karakoram-Himalaya during 1999–2011. Cryosphere 7, 1263-1286 (2013).
67. Sevestre, H. & Benn, D.I. Climatic and geometric controls on the global distribution of surge-type glaciers: implications for a unifying model of surging. *J. Glaciol.* **61**(228), 646-662 (2015).
68. Kääb A. & Vollmer M. Surface geometry, thickness changes and flow fields on creeping mountain permafrost: automatic extraction by digital image analysis*. Permafrost Periglacial Process.***11**, 315-332 (2000).
69. Pfeffer, W.T. et al. The Randolph Glacier Inventory: a globally complete inventory of glaciers, *J. Glaciol*. **60** (221), 522-537 (2014).
70. Heid, T. & Kääb, A. Evaluation of existing image matching methods for deriving glacier surface displacements globally from optical satellite imagery, *Remote Sens. Environ*, **118**, 339-355 (2012).
